# DASC-LOT Framework and S^3^ Metric: A Novel Evaluation and Benchmarking Method to Assess Initiation, Duration, and Spectrum of Antimicrobial Usage at Inpatient Hospital Settings

**DOI:** 10.1093/cid/ciag389

**Published:** 2026-06-25

**Authors:** Michihiko Goto, Shungo Imai, Hyunkeun Cho, James A Merchant, Matthew Bidwell Goetz, Eli N Perencevich, Bruce Alexander, Qianyi Shi, Andrea Holcombe, Daniel J Livorsi

**Affiliations:** Center for Access & Delivery Research and Evaluation (CADRE), Iowa City VA Health Care System, Iowa City, Iowa, USA; Department of Internal Medicine, University of Iowa Carver College of Medicine, Iowa City, Iowa, USA; Department of Internal Medicine, University of Iowa Carver College of Medicine, Iowa City, Iowa, USA; Department of Drug Informatics, Keio University Faculty of Pharmacy, Tokyo, Japan; Center for Access & Delivery Research and Evaluation (CADRE), Iowa City VA Health Care System, Iowa City, Iowa, USA; Division of Biostatistics and Bioinformatics, Herbert Wertheim School of Public Health and Human Longevity Science, University of California San Diego, La Jolla, California, USA; Center for Access & Delivery Research and Evaluation (CADRE), Iowa City VA Health Care System, Iowa City, Iowa, USA; Infectious Diseases Section, VA Greater Los Angeles Health Care System, Los Angeles, California, USA; Department of Internal Medicine, David Geffen School of Medicine at University of California Los Angeles, Los Angeles, California, USA; Center for Access & Delivery Research and Evaluation (CADRE), Iowa City VA Health Care System, Iowa City, Iowa, USA; Department of Internal Medicine, University of Iowa Carver College of Medicine, Iowa City, Iowa, USA; Center for Access & Delivery Research and Evaluation (CADRE), Iowa City VA Health Care System, Iowa City, Iowa, USA; Center for Access & Delivery Research and Evaluation (CADRE), Iowa City VA Health Care System, Iowa City, Iowa, USA; Department of Internal Medicine, University of Iowa Carver College of Medicine, Iowa City, Iowa, USA; Department of Internal Medicine, University of Iowa Carver College of Medicine, Iowa City, Iowa, USA; Center for Access & Delivery Research and Evaluation (CADRE), Iowa City VA Health Care System, Iowa City, Iowa, USA; Department of Internal Medicine, University of Iowa Carver College of Medicine, Iowa City, Iowa, USA

**Keywords:** antimicrobial stewardship, benchmarking, inpatient antimicrobial use, risk-adjustment, mathematical framework

## Abstract

**Background:**

Inpatient antimicrobial stewardship programs (ASPs) promote avoiding unnecessary initiation, excessively long duration, and overly broad-spectrum selection of antimicrobials, but currently available metrics do not adequately reflect or distinguish these 3 components. We aimed to develop a novel framework that captures them while providing information specific to each, based on days of antimicrobial spectrum coverage (DASC) and length of therapy (LOT).

**Methods:**

We developed a 3-level conditional modeling framework to extract hospital-level variability, risk-adjusted for 3 components (initiation, duration, and spectrum). This was applied to data from 118 Veterans Health Administration (VHA) hospitals, with models built on 2022–2023 data and validated with 2024 data. Patient demographics, intensive care status, admitting specialty, 86 comorbidities, and 224 procedure categories were considered candidate variables for risk adjustment. Overall hospital performance was evaluated using a composite metric that integrated 3 components (“S^3^ [start, stop, select] Metric”) and was visualized in a radar chart for each hospital.

**Results:**

The cohort included 727 958 unique patients with 9 363 922 days present. Hospital-level usage ranged widely (DASC per 1000 DP: 1311–5275 [interquartile range (IQR): 2738–3563]; LOT per 1000 DP: 132.2–517.6 [IQR: 301.0–367.5]). Risk-adjustment models included 116 variables for initiation, 77 for duration, and 91 for spectrum components. S^3^ metrics ranged from 0.703 to 1.572 (IQR: 0.906–1.086). A 3-component evaluation could provide specific information on each hospital's usage patterns.

**Conclusions:**

We propose a DASC-LOT framework and S^3^ metric to assess ASP practices in initiation, duration, and spectrum separately while providing overall composite benchmarking.

## Teaser

We introduce DASC-LOT framework and S^3^ metric, a novel antimicrobial usage evaluation method that disaggregates initiation, duration, and spectrum components. Using hierarchical models, we estimated hospital-specific risk-adjusted usage, visualized performance, and enabled actionable antimicrobial stewardship benchmarking across diverse hospitals.

**(See the Editorial Commentary by Hojat and Suchindran on pages e484–6.)**

Antimicrobial stewardship programs (ASPs) are now required or strongly encouraged in many healthcare systems and have reduced inappropriate antimicrobial exposure and improved patient outcomes [1–4]. As ASPs mature, however, the limitations of conventional metrics for evaluating the progress and performance of antimicrobial use through benchmarking or longitudinal tracking are increasingly recognized.

Widely used hospital-level metrics, such as days of therapy (DOT) per 1000 days present (DP) and the Standardized Antimicrobial Administration Ratio (SAAR) of the National Healthcare Safety Network (NHSN), focus on aggregate volume, with or without risk adjustments [5–9]. These benchmarks collapse multiple prescribing behaviors into a single number. However, in practice, at least three distinct decisions determine a patient's antimicrobial exposure that ASPs aim to improve: (1) whether antimicrobials are started, (2) how long they are continued, and (3) how broad the selected spectrum is [10]. Available metrics cannot disentangle these components, limiting their utility for designing targeted interventions. In addition, DOT per DP and SAAR do not directly assess ASPs’ efforts to avoid the use of excessively broad-spectrum therapy [7, 11]. They also count concurrently used multiple antimicrobials separately and can penalize appropriate combination therapies [12].

Spectrum-informed metrics have attempted to refine this picture. Spectrum scoring systems and measures, such as Days of Antimicrobial Spectrum Coverage (DASC), weight antimicrobial days according to expected breadth of activity, providing a more nuanced and comprehensive view of exposure, particularly for broad-spectrum agents associated with resistance and *Clostridioides difficile* infection [12–14]. Yet DASC is typically analyzed as a single composite outcome. It quantifies the extent of spectrum-weighted antimicrobial use but does not determine whether excess exposure reflects high rates of initiation, prolonged courses, or a preference for broad-spectrum agents [12]. For stewardship teams and hospital leaders, this distinction is crucial: the optimal intervention will differ for a hospital that “over-starts” antimicrobials, one that treats for too long, or one that primarily uses overly broad-spectrum antimicrobials [15].

Benchmarking adds further complexity. Hospitals differ in case mix, acuity, surgical volume, and the prevalence of complex comorbidities. Standardized Antimicrobial Administration Ratio, currently the only risk-adjusted benchmarking metric operationalized at a nationwide scale, attempted to perform risk adjustment using hospital- and ward-level factors [8]. However, its content validity as a benchmarking metric has been questioned because it does not incorporate patient-level factors [11]. There is no widely accepted framework that simultaneously captures initiation, duration, and spectrum, accounts for both patient- and hospital-level heterogeneity, and summarizes results in a way that clinicians and administrators can readily interpret with specific feedback on each component.

To address these limitations, we developed the DASC–Length of Therapy (DASC-LOT) framework and the S^3^ (Start, Stop, Select) metric, a joint, three-component approach that separately evaluates hospital-level patterns of antimicrobial initiation, therapy duration, and spectrum, while also yielding an overall composite benchmarking metric. Using patient-level data from all acute-care hospitals within the Veterans Health Administration (VHA), we constructed risk-adjusted models to estimate expected DASC and LOT for each hospital's case mix, and derived hospital-specific parameters that quantify the likelihood of antimicrobial initiation, the duration of therapy once started, and the breadth of the selected spectrum. In this study, we describe this novel approach and apply it to contemporary VHA data to quantify variation in spectrum-weighted antimicrobial use across hospitals, disaggregated into initiation, duration, and spectrum components, and illustrate how these component-specific profiles can potentially guide local and national stewardship efforts.

## METHODS

### Mathematical Framework

For each hospitalization, LOT was defined as the number of inpatient calendar days during which at least 1 systemic antibacterial was administered, regardless of the number of agents administered on a given day [16, 17]. Unlike DOT, LOT does not count concurrently used multiple antimicrobials separately and would not be inflated by combination therapy. Days present were defined as the number of calendar days a patient spent in a specific location for any part of that day [18]. For each hospital day with antimicrobial use, we calculated the spectrum score for all systemic antibacterials administered that day and summed these scores across the hospitalization to yield DASC [12].

We conceptualized hospital antimicrobial use as arising from three interrelated but separate decisions during an inpatient stay (Figure 1 and Supplementary Figure 1): (1) whether to initiate systemic antimicrobials, (2) how long to continue therapy once initiated with the given duration of hospital stay, and (3) how broad a spectrum of activity to employ with the given duration of therapy. For each hospitalization *i* in the hospital *j*, we define a binary indicator of antimicrobial initiation, a count outcome for LOT among treated patients (with DP as the denominator), and a spectrum-weighted outcome based on DASC (with LOT as the denominator).

**Figure 1.**
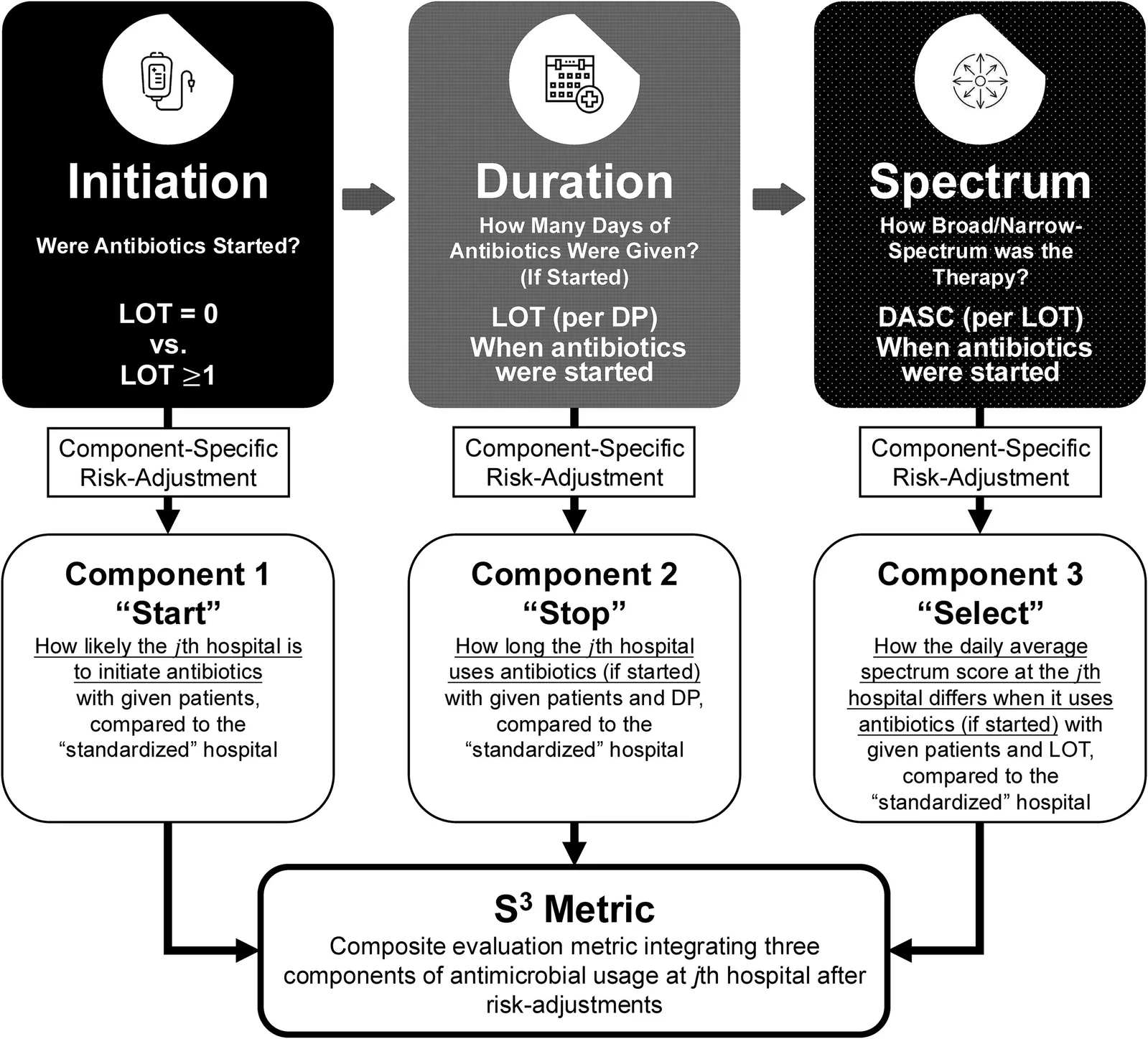
A graphical representation of the DASC-LOT framework to extract hospital-level variability with risk adjustment for 3 components. Refer to Supplementary Figure 1 for the detailed mathematical framework. Abbreviations: DASC, days of antimicrobial spectrum coverage; DP, days present; LOT, length of therapy.

DASC-LOT framework treats these as conditional stages along a patient's treatment pathway. Initiation is defined for all hospitalizations; LOT and DASC are defined only among those with antimicrobial usage. Each stage is modeled with a mixed-effects regression that adjusts for patient-level characteristics and incorporates hospital-level random intercepts. These random intercepts provide interpretable summaries of hospital tendencies to start antimicrobials, prolong courses, and select broader regimens. They are subsequently combined into component-specific and composite benchmarking metrics (“S^3^ metric”) while accounting for patient-level factors. Details of the modeling approach are described in the Supplementary Technical Supplement.

### Study Design and Data Source

To demonstrate how the DASC-LOT framework can be applied to real-world data, we conducted a retrospective cohort study of adult acute-care hospitalizations within the VHA. All VHA acute-care hospitals with complete electronic medication administration data during the study period were eligible. We included hospitalizations for adults aged ≥18 years in all acute-care beds and excluded stays confined to long-term care, mental health, or residential rehabilitation units, as per the NHSN protocol. Patients could contribute multiple hospitalizations; each qualifying stay was treated as a separate observation.

Data were obtained from the Corporate Data Warehouse, which integrates administrative, pharmacy, laboratory, and clinical information across all VHA facilities. Antimicrobial exposures were ascertained from bar-code medication administration and pharmacy dispensing records. Demographics, comorbid conditions, admitting service, intensive care unit (ICU) utilization, and procedures performed were obtained from standardized encounter-level tables. Denominators for hospital-level metrics (ie, DP) were derived from bed-section and admission/discharge records.

### Variable Definitions

Systemic antibacterial agents were defined as agents administered via systemic routes (ie, injection or oral administration) [18] and were measured using DASC and LOT, as described above. Covariates included age, sex, admitting specialty (surgical specialties or otherwise), ICU status, month of hospital stay, comorbidities, and procedures performed within 48 hours before or during the hospital stay. Comorbidities were obtained as International Classification of Diseases, Tenth Revision (ICD-10) codes and classified into 86 categories using Hierarchical Condition Categories, version 24 [19]. Procedures were obtained from inpatient and outpatient procedure records recorded as ICD-10 procedure codes or Current Procedural Terminology codes. Those codes were classified into 224 categories based on the Clinical Classifications Software [20]. To ensure data availability, reproducibility, and generalizability beyond the VHA system, we limited patient-level variables to those commonly available in private-sector billing claims. Comorbidities and procedures were considered candidate variables if at least 0.5% of included patients had them and showed significant univariate associations with the dependent variable in each component. Hospital characteristics (eg, facility complexity level, teaching status) were used in descriptive analyses but were excluded from the primary case-mix adjustment because our prior analysis found them not to be significant after patient-level risk adjustments [21].

### Statistical Analysis

We implemented the DASC-LOT framework using a sequential, component-wise mixed-modeling strategy aligned with the conditional structure, as illustrated in Figure 1, because currently no frequentist statistical software package supports a fully joint multivariate mixed-effects model that combines a binary outcome and zero-truncated count outcomes with random effects. All analyses were conducted in R version 4.5.0 (R Foundation for Statistical Computing, Vienna, Austria). Formal mathematical notation and model equations for all three components, as well as end-to-end R implementation scripts, are provided in the Supplementary Technical Supplement.

To define parsimonious and stable covariate sets for each component, we first performed variable selection by least absolute shrinkage and selection operator separately for initiation, LOT, and DASC [22, 23]. For each component, we fit penalized regression models on 10 randomly drawn subsets of the cohort. Within each subset, we selected the tuning parameter (lambda) by 5-fold cross-validation. Variables with non-zero coefficients in at least 8 of 10 subsets were retained and included as fixed effects in the final mixed-effects models described below.

For antimicrobial initiation, we fit a logistic mixed-effects model with receipt of any systemic antimicrobial as the outcome [21, 24–26] and a hospital-specific random intercept capturing each facility's baseline propensity to start antimicrobials after adjustment.

Among hospitalizations with initiation (ie, any antimicrobial administration), we modeled LOT using a zero-truncated negative binomial mixed-effects model with a hospital-level random intercept to account for overdispersion and the restriction that LOT ≥ 1, with a hospital-level random intercept capturing relative tendencies to treat for longer or shorter durations than expected, conditional on therapy initiation. Days present was included as an offset variable with a logarithmic transformation because the number of inpatient antimicrobial days is constrained by the duration of hospitalization. Therefore, this model estimates hospital-level differences in LOT relative to inpatient time at risk, conditional on antimicrobial initiation, rather than simply comparing unadjusted LOT across hospitals.

Among hospitalizations with LOT ≥ 1 (ie, any antimicrobial administration), we modeled total DASC using a zero-truncated negative binomial mixed-effects model with log(*LOT*) as an offset, and a hospital-level random intercept to account for relative tendencies to use broader or narrower than expected, conditional on therapy duration. We fitted models using the 2022–2023 data and applied fixed effects to the 2024 data to estimate 2024 hospital-specific random intercepts.

Outputs from the three models were combined to generate hospital-level benchmarks. For each hospitalization, we computed (1) the case-mix-adjusted predicted probability of antimicrobial initiation, (2) the predicted LOT given initiation probability, and (3) the predicted DASC given initiation probability and expected duration, all with hospital-specific random intercepts. We also computed expected values under a reference hospital by setting the hospital random intercept to zero. Patient-level expected values were aggregated within hospitals to yield hospital-specific expected totals for initiation, LOT, and DASC.

We summarized the overall spectrum-weighted use using predicted-to-expected ratios by integrating all three components (“S^3^ metric”; Figure 1 and Supplementary Figure 1) [21, 27, 28]. The numerator is the sum of hospital-specific model-based predictions that incorporate random intercepts; the denominator is the sum of reference-hospital expectations. Ratios >1 indicate higher-than-expected use; ratios <1 indicate lower-than-expected use after case-mix adjustment.

We used a 2-stage cluster bootstrap (resampling hospitals, then patients) with 1000 replicates to derive 95% CIs for S^3^ metrics and random effects in all components [29]. For visualization, we exponentiated the random intercepts for initiation, LOT, and DASC (odds ratios for initiation and rate ratios for LOT and DASC) and displayed them as triangular radar plots (Figure 2), highlighting each hospital's relative profile across the three components. Although the three-component models were fit sequentially, they are structurally linked by the conditional pathway; the combined S^3^ metric thus approximates a joint estimation framework for hospital-level antimicrobial use. All *P* values are 2-sided, and a significance level of 0.05 was used.

**Figure 2.**
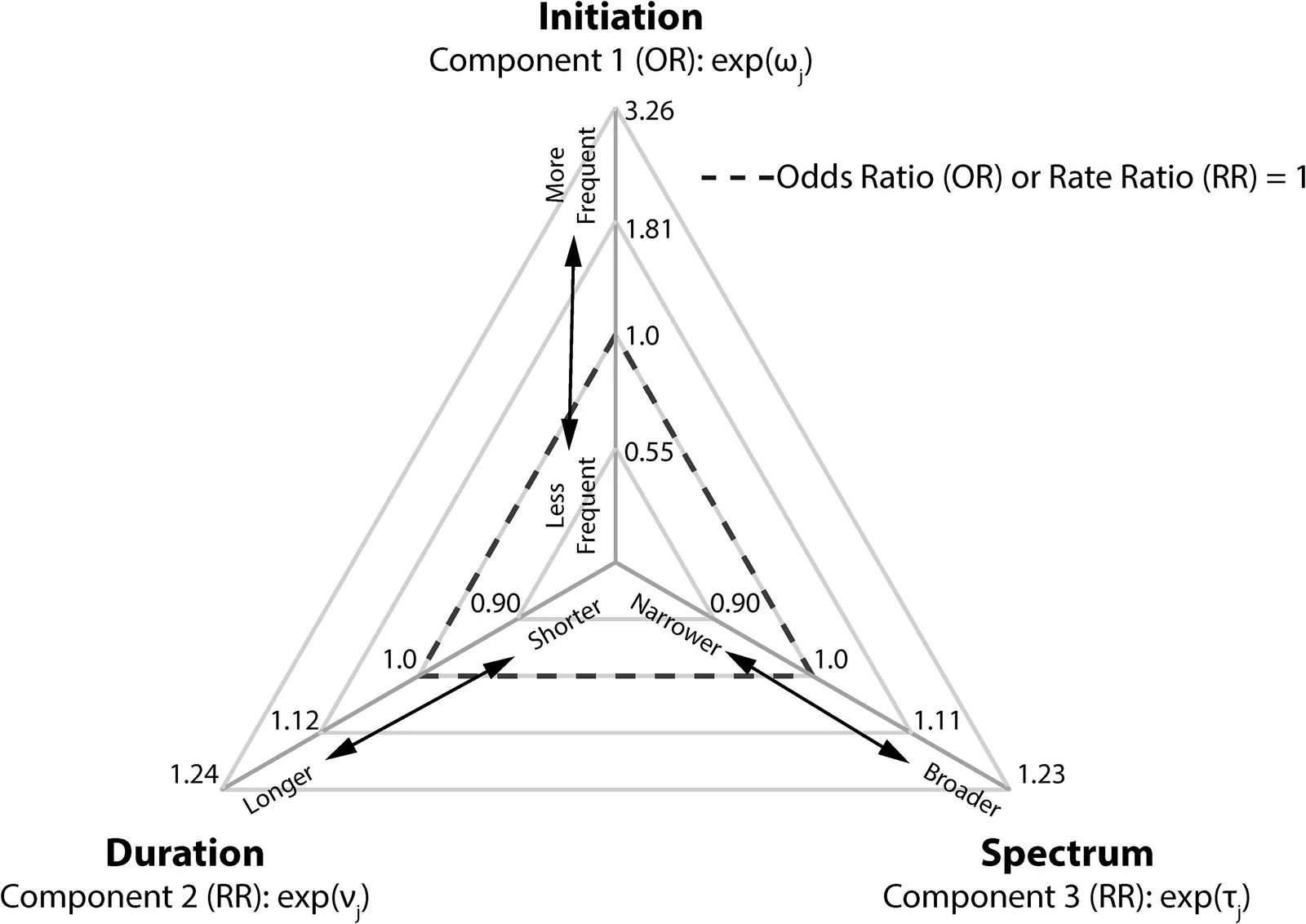
Triangular radar plot used to visualize hospital-level performance across the 3 components of the DASC–LOT framework. Reference grid values were logarithmically spaced around 1.0 to symmetrically represent proportional increases and decreases. Abbreviations: DASC, days of antimicrobial spectrum coverage; LOT, length of therapy.

### Ethics

This study was conducted within the VHA under approval from the institutional review board at the University of Iowa and the research and development committee at the Iowa City VA Health Care System. The analysis used existing electronic health records and administrative data and posed minimal risk to participants; informed consent was therefore waived.

## RESULTS

### Characteristics of Facilities and Patients

A total of 118 VHA acute-care hospitals were included, and the cohort included 727 958 unique patients with 9 363 922 DP. Hospital-level usage ranged widely (DASC per 1000 DP: 1311–5275 [interquartile range (IQR): 2738–3563]; LOT per 1000 DP: 132.2–517.6 [IQR: 301.0–367.5]). Details of hospital and patient characteristics, stratified by development (2022–2023) and validation (2024) cohorts, are shown in Table 1 and Supplementary Table 1. There were no observations with missing data on antimicrobial administration or covariates used in the risk adjustments.

**Table 1.** Characteristics of Facilities and Selected Patient Characteristics

	2022–2023	2024
Facilities	118	118
VA Complexity Level [30]		
1a (Facility width high volume, high-risk patients, most complex clinical programs, and large research and teaching programs)	38 (32.2%)	38 (32.2%)
1b (Facilities with medium-to-high volume, high-risk patients, many complex clinical programs, and medium-large research and teaching programs)	28 (23.7%)	28 (23.7%)
1c (Facilities with medium-to-high volume, medium risk patients, some complex clinical programs, and medium sized research and teaching programs)	25 (21.2%)	25 (21.2%)
2 (Facilities with medium volume, low risk patients, few complex clinical programs, and small or no research and teaching programs)	13 (11.0%)	13 (11.0%)
3 (Facilities with low volume, low risk patients, few or no complex clinical programs, and small or no research and teaching programs)	14 (11.9%)	14 (11.9%)
No. of wards, median (IQR)	6 (4–8)	6 (4–8)
Total DP per Facility, median (IQR)	49 211 (21 134–74 887)	24 504 (9988–36 316)
Antimicrobial Initiation at Each Facility		
Frequency of Antimicrobial Initiation, median (IQR)	39.7% (37.0%–43.7%)	40.3% (37.7%–44.4%)
LOT per Facility		
Total, median (IQR)	15 932 (6878–23 658)	8254 (3920–12 559)
Per 1000 DP, median (IQR)	324.4 (295.9–363.0)	340.3 (309.5–373.8)
DASC per Facility		
Total, median (IQR)	156 083 (72 015–223 227)	79 050 (36 628–111 179)
Per 1000 DP, median (IQR)	3110 (2688–3533)	3140 (2853–3640)
Patients	**2022–2023**	**2024**
Patients (n)	559 991	332 788
Number of Hospital Admissions	931 948	484 107
Length of Stay per Hospital Admission in Days, median (IQR)	4 (2–7)	4 (2–7)
DP (Total)	6 257 368	3 106 554
Antimicrobial Initiation	39.7%	40.3%
LOT (Total)	2 029 960	1 030 447
DASC (Total)	19 462 412	9 890 372
Age, median (IQR)	70 (61–76)	71 (62–77)
Male Gender	517 522 (92.4%)	306 657 (92.1%)

Abbreviations: DASC, days of antimicrobial spectrum coverage; DP, days present; ICU, intensive care unit; IQR, interquartile range; LOT, length of therapy; VA, Department of Veterans Affairs.

### Hospital-Level Benchmarking of Antimicrobial Usage Based on the DASC–LOT Framework

Risk-adjustment models included 116 variables for initiation, 77 for duration, and 91 for spectrum components. Details of the models in each component are provided in Supplementary Table 2. Figure 3 displays hospital-level S^3^ metrics in the 2024 validation cohort, estimated by applying fixed-effect coefficients derived from the 2022–2023 development cohort and re-estimating hospital-specific random intercepts. Across hospitals, S^3^ metrics ranged from 0.703 to 1.572 (IQR: 0.906–1.086).

**Figure 3.**
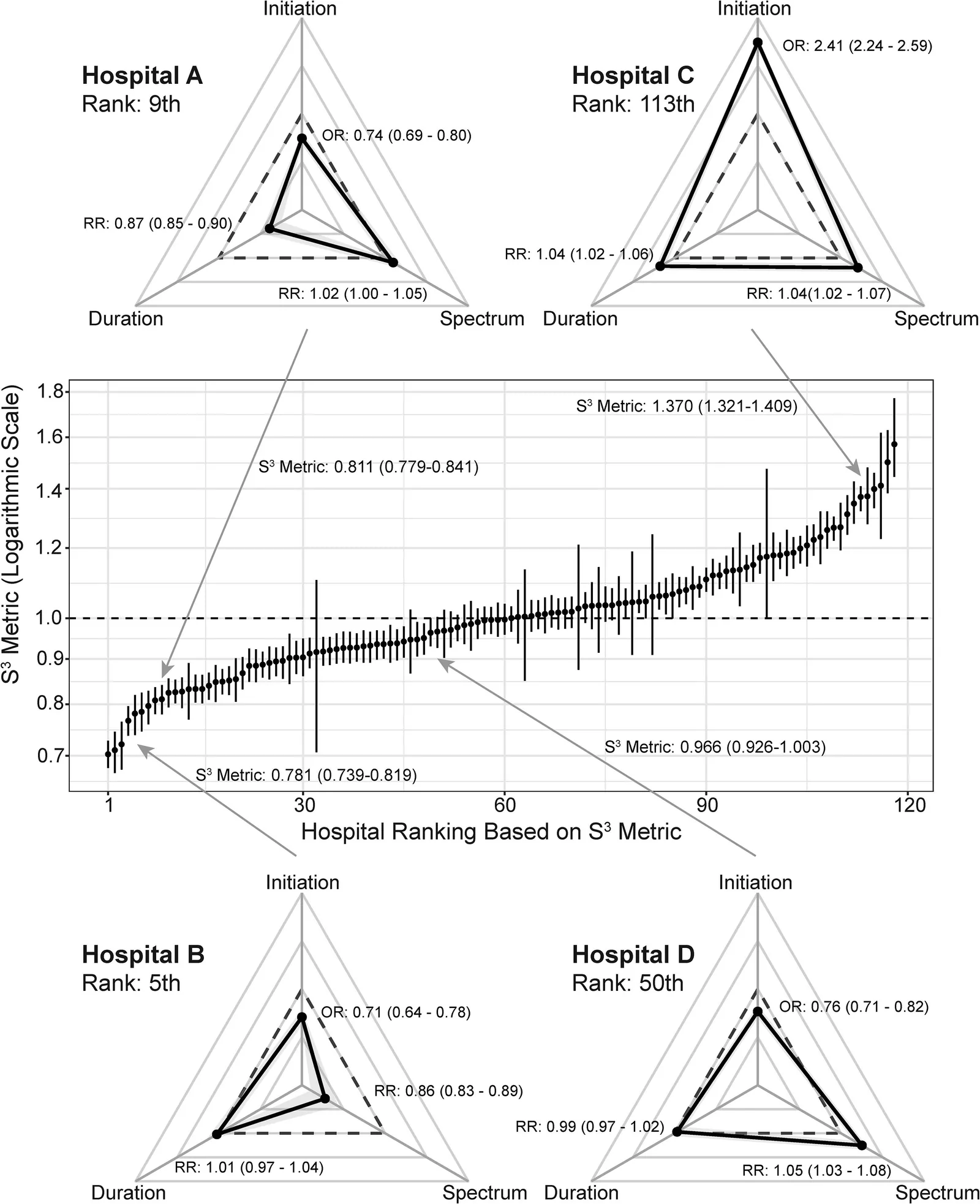
Hospital-level benchmarking of antimicrobial spectrum by using S^3^ metrics and component-level radar plots for representative hospitals. Hospitals are ranked from lowest to highest S^3^ metrics along the *x*-axis. Points represent hospital-specific S^3^ metric estimates derived from the DASC–LOT framework, and vertical lines indicate 95% CIs obtained using a hierarchical cluster bootstrap. In component-level radar plots (ie, hospitals A to D), each triangular plot depicts the hospital-specific initiation, duration (LOT), and spectrum (DASC) components, estimated using exponentiated random intercepts from mixed-effects models. The dashed reference triangle represents expected performance (S^3^ metric = 1) after risk adjustment. The gray-shaded band indicates the 95% CIs for the component-specific estimates. Abbreviations: CI, confidence interval; DASC, days of antimicrobial spectrum coverage; LOT, length of therapy; OR, odds ratio; RR, relative ratio.

### Illustrative Examples of Facility Evaluations Using the DASC–LOT Framework

Four representative hospitals (Hospitals A–D) with differing S^3^ metrics and component-level profiles are shown in Figure 3. Hospitals A and B both demonstrated low S^3^ metrics (Hospital A: 0.811 [95% CI, 0.779–0.841]; Hospital B: 0.781 [95% CI, 0.739–0.819]), suggesting similarly low antimicrobial use when considering 3 components at the aggregate level. However, their underlying prescribing patterns differed substantially. Hospital A tended to initiate less frequently, with shorter durations and a moderate spectrum of antimicrobial selection, whereas Hospital B showed relatively longer durations and a narrower spectrum of antimicrobial selection. In contrast, Hospital C demonstrated a high S^3^ metric (1.370 [95% CI, 1.321–1.409]), driven by all three components, but particularly with a tendency to initiate antimicrobial therapy more frequently. Hospital D had an S^3^ metric closer to a hypothetical “standardized” hospital (0.966 [95% CI, 0.926–1.003]); however, a closer three-axis evaluation showed a tendency to initiate antimicrobials less frequently, with broader-spectrum agent selection.

## DISCUSSION

In this study, we propose the DASC-LOT framework as a three-component benchmarking approach that explicitly separates antimicrobial initiation, duration (LOT), and spectrum intensity (DASC) with comprehensive risk adjustments separately for each component, while yielding a single composite metric for overall benchmarking (S^3^ Metric). When this approach was applied to 118 VHA acute care hospitals, hospital-level S^3^ metrics ranged from 0.703 to 1.572 (IQR, 0.906–1.086), indicating that substantial differences in antimicrobial usage persisted beyond case-mix differences captured by administrative covariates. Importantly, the framework also supports interpretability: hospitals with similar overall S^3^ metrics may exhibit distinct “phenotypes” when disaggregated into initiation, duration, and spectrum components, illustrating why a single-number benchmark alone can obscure actionable stewardship targets.

Although other metrics have been proposed and adopted for benchmarking antimicrobial use, they capture important but incomplete aspects of it. Days of therapy is the most widely used patient-level basic metric for quantifying antimicrobial administration in the United States, including within the CDC's NHSN [5, 6]. However, DOT does not account for antimicrobial spectrum and may inadvertently penalize appropriate combination therapy [7]. Length of therapy, defined as the number of unique days on which a patient receives any antimicrobial [16, 17], avoids double-counting in the context of combination regimens but similarly lacks the ability to reflect the breadth of antimicrobial activity. Standardized Antimicrobial Administration Ratio is based on DOT and applies risk adjustment using hospital- and unit-level variables by single-level negative binomial regression models [8]. However, concerns have been raised about SAAR's content validity and robustness for benchmarking because it does not incorporate patient-level case mix or antimicrobial spectrum [11]. Overall, these metrics share 2 common limitations: a lack of assessment of antimicrobial spectrum and component-specific feedback. To address these issues, spectrum-based metrics such as the Antimicrobial Spectrum Index or DASC have been developed [12, 31]. Previous studies suggest limited correlations between DOT and spectrum-based measures (eg, DOT per 1000 DP and DASC per DOT), underscoring that reductions in antimicrobial volume do not necessarily correspond to narrower-spectrum prescribing [13, 14, 32–34]. These reports suggest that duration and spectrum capture different aspects of antimicrobial use and may warrant separate consideration.

The central advantage of the DASC-LOT framework is that it aligns benchmarking with the way stewardship decisions are made in practice. Clinicians and ASPs make (at least) 3 separable decisions: whether to start antimicrobials, how long to continue, and how broad to treat on treated days. For example, hospitals with high initiation rates may benefit most from diagnostic stewardship and syndrome-based pathways that reduce unnecessary initiation. Hospitals with prolonged durations may be best served by default-duration order sets and audit-and-feedback that focus on stop dates and reassessment of ongoing indications. Hospitals with higher spectrum intensity may benefit from empiric guideline refinement, restriction/prior authorization of selected broad agents, rapid diagnostic integration, and structured de-escalation prompts.

While our current study focused on benchmarking at the hospital level, the DASC-LOT framework can also be used for internal benchmarking and chronological assessment at an individual hospital by including unit- or provider-specific random intercepts. In addition, future expansion of the DASC-LOT framework could include more detailed hierarchical structures (eg, unit-specific random intercepts nested within hospitals) to allow more granular, stratified reporting (eg, ICU-specific reporting). Such applications could help ASPs determine whether changes in antimicrobial use are driven primarily by initiation, duration, or spectrum selection.

Our approach also complements, but is distinct from, recent machine learning (ML) works on antimicrobial-use prediction and risk adjustment [35]. For example, Moehring et al [36] showed that ML models incorporating a large number of patient-level variables and nonlinear relationships can accurately identify antimicrobial initiation and estimate DOT, suggesting that such approaches may improve measurement of antimicrobial use. Subsequent multicenter studies further demonstrated that ML-based risk adjustment using encounter-level diagnosis and procedure data was feasible across heterogeneous hospital systems and achieved higher model accuracy than conventional approaches relying on facility-level variables [37]. However, these ML models offer limited interpretability for inter-hospital comparisons and provide little insight into which specific components of antimicrobial use contribute to observed differences between hospitals. The DASC-LOT framework addresses gaps in existing metrics and prior work, providing a structured approach to examining how different components contribute to overall antimicrobial use, which is difficult to assess with conventional single-number benchmarks.

This study has several limitations. First, although the DASC-LOT framework arguably has stronger content validity (ie, it conceptually captures key components of antimicrobial use), its construct validity (ie, the extent to which these components relate to stewardship activities or clinical outcomes) has yet to be examined. Therefore, future studies are needed to evaluate how hospital-level evaluations from the DASC-LOT framework relate to ASP activities and their associated outcomes. Second, to ensure data availability, reproducibility, and generalizability, we limited the set of patient-level covariates used for risk adjustment to those commonly available in private-sector administrative claims. Although this design enhances the feasibility of application across diverse healthcare systems, other clinically relevant information not routinely available in administrative data (eg, microbiology reports) may have important implications for risk adjustment. Third, post-discharge antimicrobial use was not captured in this analysis, which may represent a substantial proportion of hospital-related antimicrobial exposure [38, 39]. Fourth, our implementation employed a sequential, component-wise modeling strategy that aligns with the conditional structure. While this preserves the conceptual factorization of antimicrobial use into conditional stages, future extensions could explore joint multivariate mixed-effects modeling (including correlations among hospital effects across components) to better characterize whether hospitals that initiate more also tend to treat longer or broader after adjustment. Lastly, we used data from VHA acute-care hospitals, in which older and male patients are overrepresented. However, VHA is the only integrated healthcare system in the United States with a presence in all states and territories and encompasses all hospital levels and varying levels of complexity, providing an excellent example of how this framework can work in multi-hospital systems or for nationwide benchmarking. When applying this framework to non-VHA settings, we speculate that retraining or recalibrating the model will be necessary; however, we also believe it is feasible to harmonize data from VHA and non-VHA sources while maintaining data security and privacy through a federated learning approach [40].

## CONCLUSIONS

In conclusion, we propose a novel ASP evaluation method, the DASC-LOT framework, and the S^3^ metric, which provides a risk-adjusted approach to benchmarking antimicrobial use, with specific evaluations of 3 components (initiation, duration, and spectrum). By integrating spectrum-weighted measures with patient-level adjustment, it provides interpretable, component-specific feedback that conventional metrics cannot provide. This framework can potentially help support more targeted antimicrobial stewardship interventions and enable fairer comparisons across hospitals in diverse healthcare settings.

## Supplementary Material

ciag389_Supplementary_Data

## Supplementary Data

Supplementary materials are available at *Clinical Infectious Diseases* online. Consisting of data provided by the authors to benefit the reader, the posted materials are not copyedited and are the sole responsibility of the authors, so questions or comments should be addressed to the corresponding author.

## References

[1] Karanika S, Paudel S, Grigoras C, Kalbasi A, Mylonakis E. Systematic review and meta-analysis of clinical and economic outcomes from the implementation of hospital-based antimicrobial stewardship programs. Antimicrob Agents Chemother. 2016;60:4840–52. 10.1128/AAC.00825-16 PMC4958232 27246783

[2] Ohl CA, Dodds Ashley ES. Antimicrobial stewardship programs in community hospitals: the evidence base and case studies. Clin Infect Dis. 2011;53:S23–8; quiz S29–30. 10.1093/cid/cir365 21795725

[3] Schuts EC, Hulscher MEJL, Mouton JW, et al Current evidence on hospital antimicrobial stewardship objectives: a systematic review and meta-analysis. Lancet Infect Dis. 2016;16:847–56. 10.1016/S1473-3099(16)00065-7 26947617

[4] Kelly AA, Jones MM, Echevarria KL, et al A report of the efforts of the veterans health administration national antimicrobial stewardship initiative. Infect Control Hosp Epidemiol. 2017;38:513–20. 10.1017/ice.2016.328 28118861

[5] Bumpass JB, McDaneld PM, DePestel DD, et al Outcomes and metrics for antimicrobial stewardship: survey of physicians and pharmacists. Clin Infect Dis. 2014;59:S108–11. 10.1093/cid/ciu545 25261537

[6] Moehring RW, Anderson DJ, Cochran RL, Hicks LA, Srinivasan A, Dodds Ashley ES. Expert consensus on metrics to assess the impact of patient-level antimicrobial stewardship interventions in acute-care settings. Clin Infect Dis 2017;64:377–83. 10.1093/cid/ciw787 PMC5241782 27927866

[7] Yarrington ME, Moehring RW. Basic, advanced, and novel metrics to guide antibiotic use assessments. Curr Treat Options Infect Dis. 2019;11:145–60.

[8] O'Leary EN, Edwards JR, Srinivasan A, et al National healthcare safety network standardized antimicrobial administration ratios (SAARs): a progress report and risk modeling update using 2017 data. Clin Infect Dis 2020;71:e702–9. 10.1093/cid/ciaa326 PMC7879497 32215655

[9] van Santen KL, Edwards JR, Webb AK, et al The standardized antimicrobial administration ratio: a new metric for measuring and comparing antibiotic use. Clin Infect Dis. 2018;67:179–85. 10.1093/cid/ciy075 29409000

[10] Tamma PD, Miller MA, Cosgrove SE. Rethinking how antibiotics are prescribed: incorporating the 4 moments of antibiotic decision making into clinical practice. JAMA. 2019;321:139–40. 10.1001/jama.2018.19509 30589917

[11] Shively NR, Morgan DJ. The CDC antimicrobial use measure is not ready for public reporting or value-based programs. Antimicrob Steward Healthc Epidemiol. 2023;3:. 10.1017/ash.2023.143 PMC10127230 37113208

[12] Kakiuchi S, Livorsi DJ, Perencevich EN, et al Days of antibiotic spectrum coverage: a novel metric for inpatient antibiotic consumption. Clin Infect Dis. 2022;75:567–76. 10.1093/cid/ciab1034 34910130

[13] Suzuki H, Heintz BH, Livorsi DJ, Perencevich EN, Goto M. Tracking antimicrobial stewardship activities beyond days of therapy (DOT): comparison of days of antibiotic spectrum coverage (DASC) and DOT at a single center. Infect Control Hosp Epidemiol. 2023;44:934–7. 10.1017/ice.2022.312 PMC10262162 36625069

[14] Murakami S, Akazawa M, Honda H. Difference between days of therapy and days of antibiotic spectrum coverage in an inpatient antimicrobial stewardship program: vector autoregressive models for time-series analysis. Infect Control Hosp Epidemiol. 2024;45:459–66. 10.1017/ice.2023.197 37937440

[15] Tinker NJ, Foster RA, Webb BJ, Haydoura S, Buckel WR, Stenehjem EA. Interventions to optimize antimicrobial stewardship. Antimicrob Steward Healthc Epidemiol. 2021;1:e46. 10.1017/ash.2021.210 PMC9495515 36168471

[16] Berrington A . Antimicrobial prescribing in hospitals: be careful what you measure. J Antimicrob Chemother. 2010;65:163–8. 10.1093/jac/dkp399 19884120

[17] Polk RE, Hohmann SF, Medvedev S, Ibrahim O. Benchmarking risk-adjusted adult antibacterial drug use in 70 US academic medical center hospitals. Clin Infect Dis. 2011;53:1100–10. 10.1093/cid/cir672 21998281

[18] National healthcare safety network (NHSN) surveillance for antimicrobial use and antimicrobial resistance options. Centers for Disease Control and Prevention. Available at: https://www.cdc.gov/nhsn/acute-care-hospital/aur/index.html. Accessed 17 February 2026.

[19] Report to congress: risk adjustment in medicare advantage. Centers for Medicare & Medicaid Services. Available at: https://www.cms.gov/medicare/health-plans/medicareadvtgspecratestats/risk-adjustors-items/reporttocongress. Accessed 17 February 2026.

[20] Clinical classifications software for services and procedures. Agency for Healthcare Research and Quality. Available at: https://hcup-us.ahrq.gov/toolssoftware/ccs_svcsproc/ccssvcproc.jsp. Accessed 17 February 2026.

[21] Goto M, Cho H, Merchant JA, et al Metrics selection and risk adjustment methods to benchmark inpatient antibiotic use. JAMA Network Open. 2025;8:e2514989. 10.1001/jamanetworkopen.2025.14989 PMC12159770 40498481

[22] Tibshirani R . Regression shrinkage and selection via the lasso. J R Stat Soc Series B Stat Methodol. 1996;58:267–88.

[23] Meinshausen N, Bühlmann P. Stability selection. J R Stat Soc Series B Stat Methodol. 2010;72:417–73.

[24] Austin PC, Alter DA, Tu JV. The use of fixed- and random-effects models for classifying hospitals as mortality outliers: a Monte Carlo assessment. Medical Decision Making 2003;23:526–39. 10.1177/0272989X03258443 14672113

[25] Ohl ME, Richardson KK, Goto M, Vaughan-Sarrazin M, Schweizer ML, Perencevich EN. HIV quality report cards: impact of case-mix adjustment and statistical methods. Clin Infect Dis. 2014;59:1160–7. 10.1093/cid/ciu551 25034427

[26] Pavlou M, Ambler G, Omar RZ, et al Outlier identification and monitoring of institutional or clinician performance: an overview of statistical methods and application to national audit data. BMC Health Serv Res. 2023;23:23. 10.1186/s12913-022-08995-z PMC9832645 36627627

[27] Risk-adjustment models for AMI and HF 30-day mortality methodology. Centers for Medicare & Medicaid Services. Available at: https://qualitynet.cms.gov/inpatient/measures/mortality/methodology. Accessed 17 February 2026.

[28] Daniels MJ, Gatsonis C. Hierarchical generalized linear models in the analysis of variations in health care utilization. J Am Stat Assoc. 1999;94:29–42.

[29] Field CA, Welsh AH. Bootstrapping clustered data. J R Stat Soc Series B Stat Methodol. 2007;69:369–90.

[30] Facilities staffing requirements for the veterans health administration-resource planning and methodology for the future. National Academies Press; 2019. 32293829

[31] Gerber JS, Hersh AL, Kronman MP, Newland JG, Ross RK, Metjian TA. Development and application of an antibiotic spectrum index for benchmarking antibiotic selection patterns across hospitals. Infect Control Hosp Epidemiol. 2017;38:993–7. 10.1017/ice.2017.94 28560946

[32] Kanda N, Ohbe H, Jo T, et al Trends in inpatient antimicrobial consumption using days of therapy and days of antibiotic spectrum coverage: a nationwide claims database study in Japan. J Infect Chemother. Mar 2024;30:228–35. 10.1016/j.jiac.2023.10.012 37852373

[33] Sullivan BA, Panda A, Wallman-Stokes A, et al Antibiotic spectrum index: a new tool comparing antibiotic use in three NICUs. Infect Control Hosp Epidemiol. 2022;43:1553–7. 10.1017/ice.2021.467 PMC9124723 34812135

[34] Zombori L, Paulus S, Shah MA, McGarrity O, Hatcher J. Antibiotic spectrum index as an antimicrobial stewardship tool in paediatric intensive care settings. Int J Antimicrob Agents 2023;61:106710. 10.1016/j.ijantimicag.2023.106710 36642234

[35] Pennisi F, Pinto A, Ricciardi GE, Signorelli C, Gianfredi V. Artificial intelligence in antimicrobial stewardship: a systematic review and meta-analysis of predictive performance and diagnostic accuracy. Eur J Clin Microbiol Infect Dis 2025;44:463–513. 10.1007/s10096-024-05027-y 39757287

[36] Moehring RW, Phelan M, Lofgren E, et al Development of a machine learning model using electronic health record data to identify antibiotic use among hospitalized patients. JAMA Network Open 2021;4:e213460. 10.1001/jamanetworkopen.2021.3460 PMC8008288 33779743

[37] Moehring RW, Yarrington ME, Ashley ED, et al Using encounter-level data for risk-adjustment of antimicrobial use comparisons: feasibility and variable selection. Clin Infect Dis 2026; 82:934–43. 10.1093/cid/ciaf682 41398639

[38] Livorsi DJ, Goetz MB, Alexander B, et al A comprehensive assessment of post-discharge antibiotic use across an integrated healthcare system. Infect Control Hosp Epidemiol. 2025;46:1–6. 10.1017/ice.2025.10230 PMC12620061 40958608

[39] Livorsi DJ, Merchant JA, Cho H, et al A novel risk-adjusted metric to compare hospitals on their antibiotic prescribing at hospital discharge. Clin Infect Dis. 2024;79:588–95. 10.1093/cid/ciae224 PMC11426263 38658348

[40] Li W, Tong J, Anjum MM, Mohammed N, Chen Y, Jiang X. Federated learning algorithms for generalized mixed-effects model (GLMM) on horizontally partitioned data from distributed sources. BMC Med Inform Decis Mak 2022;22:269. 10.1186/s12911-022-02014-1 PMC9569919 36244993

