# Supplementary material for: DASC-LOT Framework and S3 Metric: A Novel Evaluation and Benchmarking Method to Assess Initiation, Duration, and Spectrum of Antimicrobial Usage at Inpatient Hospital Settings
Source: Clin Infect Dis. 2026 Jun 25;83(3):e475–83. doi: 10.1093/cid/ciag389 (PMC13644869; doi:10.1093/cid/ciag389)
Supplement: Supplementary file 1 [file ciag389_supplementary_data.zip › Supplementary Figure 1 20260426.pdf]

Component 1: Probability of Antibiotic Initiation

Let Y_{1ij} be a binary variable indicating whether patient i in hospital j received any antibiotics: Y_{1ij}=1 if yes, Y_{1ij}=0 otherwise). The probability of initiation is modeled using logistic regression:

$$logit\left(P(Y_{1ij}=1)\right) = X_{ij}\beta + \omega_j$$

X_{ij} is a vector for patient-level predictors for the ith patient in the jth hospital
β is a vector of fixed-effect coefficients for patient-level predictors
$\omega_j \sim N(0, \sigma_\omega^2)$ is the hospital-specific random intercept for the jth hospital

Component 2: Length of Therapy (LOT) per Days Present (DP); Conditional Model

Given that a patient received antibiotics (Y_{1ij}=1), we assume the LOT (Y_{2ij}) follows a zero-truncated negative binomial distribution (because $Y_{2ij} \geq 1$ when $Y_{1ij} = 1$ by definition):

$$Y_{2ij} \sim ZTNB\left(\mu_{Y_{2ij}}, \theta_{Y_2}\right), Y_{2ij} = 1, 2, 3, \ldots$$

with the log-link function:

$$\log(E[Y_{2ij}|Y_{1ij}=1]) = X'_{ij}\beta' + \log(D_{ij}) + \upsilon_j$$

X'_{ij} is a vector for patient-level predictors for the ith patient in the jth hospital
β' is a vector of fixed-effect coefficients for patient-level predictors
D_{ij} is days present (offset variable)
$\upsilon_j \sim N(0, \sigma_\upsilon^2)$ is the hospital-specific random intercept for the jth hospital

Component 3: Days of Antimicrobial Spectrum Coverage (DASC) per LOT; Conditional Model

Given that a patient received antibiotics for Y_{2ij} days ($Y_{2ij} \geq 1$), we assume DASC (Y_{3ij}) follows a zero-truncated negative binomial distribution (because $Y_{3ij} \geq 1$ when $Y_{2ij} \geq 1$ by definition):

$$Y_{3ij} \sim ZTNB\left(\mu_{Y_{3ij}}, \theta_{Y_3}\right), Y_{3ij} = 1, 2, 3, \ldots$$

with the log-link function:

$$\log(E[Y_{3ij}|Y_{2ij} \geq 1]) = X''_{ij}\beta'' + \log(Y_{2ij}) + \tau_j$$

X''_{ij} is a vector for patient-level predictors for the ith patient in the jth hospital
β'' is a vector of fixed-effect coefficients for patient-level predictors
Y_{2ij} is Length of Therapy (LOT: offset variable)
$\tau_j \sim N(0, \sigma_\tau^2)$ is the hospital-specific random intercept for the jth hospital

Interpretations of Exponentiated Values for Hospital-Specific Random Intercepts

Component 1 (Initiation): $\exp(\omega_j)$is a facility-specific odds ratio to indicate how likely the jth hospital is to initiate antibiotics with given patients, compared to the "standardized" hospital
Component 2 (Duration): $\exp(\upsilon_j)$is a facility-specific rate ratio to indicate how long the jth hospital uses antibiotics (if started) with given patients and DP, compared to the "standardized" hospital
Component 3 (Spectrum): $\exp(\tau_j)$is a facility-specific rate ratio to indicate how the daily average spectrum score at the jth hospital differs when it uses antibiotics (if started) with given patients and LOT, compared to the "standardized" hospital

Composite Evaluation Integrating Three Components (S^3 Metric for jth Hospital)

$$\text{S}^3\ \text{Metric}_j = \frac{\text{Predicted DASC Specific to Hospital}_\text{j}\ (\text{with } \omega_j, \upsilon_j, \tau_j) \text{ with Given Patients, LOT, and Antibiotic Initiation Frequency}}{\text{Predicted DASC if the "Standardized" Hospital Manage the Same Patients as Hospital}_\text{j}}$$

$$= \frac{\text{Probability of Initiation with } \omega_j \times \text{Predicted DASC with Given Predicted LOT with } \upsilon_j \text{ and } \tau_j}{\text{Probability of Initiation without } \omega_j \times \text{Predicted DASC with Given Predicted LOT without } \upsilon_j \text{ and } \tau_j}$$

$$= \frac{logit^{-1}(X_{ij}\beta + \omega_j) \times \exp(X'_{ij}\beta' + X''_{ij}\beta'' + \log(D_{ij}) + \upsilon_j + \tau_j)}{logit^{-1}(X_{ij}\beta) \times exp(X'_{ij}\beta' + X''_{ij}\beta'' + log(D_{ij}))}$$
