# Supplementary material for: DASC-LOT Framework and S3 Metric: A Novel Evaluation and Benchmarking Method to Assess Initiation, Duration, and Spectrum of Antimicrobial Usage at Inpatient Hospital Settings
Source: Clin Infect Dis. 2026 Jun 25;83(3):e475–83. doi: 10.1093/cid/ciag389 (PMC13644869; doi:10.1093/cid/ciag389)
Supplement: Supplementary file 1 [file ciag389_supplementary_data.zip › Supplementary Table 1 20260304.pdf]

**Supplementary Table 1. Detailed Descriptions of Patient-Level Variables**

| Variable | Description | 2022-2023 | | 2024 | |
|---|---|---|---|---|---|
| **n** | Number of Unique Patients During Study Period | 559,991 | | 332,788 | |
| **Median Age (IQR)** | Median (Interquartile Range) | 70 (61-76) | | 71 (62-77) | |
| **Admission to ICU (n, %)** | | 66,435 | 11.90% | 39,218 | 11.80% |
| **Admission to Surgical Specialties (n, %)** | | 143,139 | 25.60% | 80,550 | 24.20% |
| **Male Gender (n, %)** | | 517,522 | 92.40% | 306,657 | 92.10% |
| **HCC Category 1 (n, %)** | HIV/AIDS | 6,172 | 1.10% | 3,813 | 1.10% |
| **HCC Category 2 (n, %)** | Septicemia, Sepsis, Systemic Inflammatory Response Syndrome/Shock | 51,939 | 9.30% | 36,305 | 10.90% |
| **HCC Category 6 (n, %)** | Opportunistic Infections | 4,227 | 0.80% | 3,069 | 0.90% |
| **HCC Category 8 (n, %)** | Metastatic Cancer and Acute Leukemia | 28,334 | 5.10% | 18,422 | 5.50% |
| **HCC Category 9 (n, %)** | Lung and Other Severe Cancers | 36,396 | 6.50% | 23,653 | 7.10% |
| **HCC Category 10 (n, %)** | Lymphoma and Other Cancers | 19,470 | 3.50% | 12,570 | 3.80% |
| **HCC Category 11 (n, %)** | Colorectal, Bladder, and Other Cancers | 37,840 | 6.80% | 24,832 | 7.50% |
| **HCC Category 12 (n, %)** | Breast, Prostate, and Other Cancers and Tumors | 65,602 | 11.70% | 43,455 | 13.10% |
| **HCC Category 17 (n, %)** | Diabetes with Acute Complications | 7,969 | 1.40% | 5,147 | 1.50% |
| **HCC Category 18 (n, %)** | Diabetes with Chronic Complications | 180,852 | 32.30% | 114,432 | 34.40% |
| **HCC Category 19 (n, %)** | Diabetes without Complication | 210,806 | 37.60% | 130,027 | 39.10% |
| **HCC Category 21 (n, %)** | Protein-Calorie Malnutrition | 38,716 | 6.90% | 28,228 | 8.50% |
| **HCC Category 22 (n, %)** | Morbid Obesity | 45,667 | 8.20% | 28,116 | 8.40% |
| **HCC Category 23 (n, %)** | Other Significant Endocrine and Metabolic Disorders | 40,851 | 7.30% | 29,851 | 9.00% |
| **HCC Category 27 (n, %)** | End-Stage Liver Disease | 12,152 | 2.20% | 8,117 | 2.40% |
| **HCC Category 28 (n, %)** | Cirrhosis of Liver | 29,159 | 5.20% | 19,280 | 5.80% |
| **HCC Category 29 (n, %)** | Chronic Hepatitis | 24,718 | 4.40% | 14,255 | 4.30% |
| **HCC Category 33 (n, %)** | Intestinal Obstruction/Perforation | 22,525 | 4.00% | 15,602 | 4.70% |
| **HCC Category 34 (n, %)** | Chronic Pancreatitis | 6,701 | 1.20% | 4,464 | 1.30% |
| **HCC Category 35 (n, %)** | Inflammatory Bowel Disease | 10,900 | 1.90% | 6,930 | 2.10% |
| **HCC Category 39 (n, %)** | Bone/Joint/Muscle Infections/Necrosis | 26,280 | 4.70% | 18,322 | 5.50% |
| **HCC Category 40 (n, %)** | Rheumatoid Arthritis and Inflammatory Connective Tissue Disease | 28,129 | 5.00% | 18,613 | 5.60% |
| **HCC Category 46 (n, %)** | Severe Hematological Disorders | 4,744 | 0.80% | 3,014 | 0.90% |
| **HCC Category 47 (n, %)** | Disorders of Immunity | 18,039 | 3.20% | 12,847 | 3.90% |
| **HCC Category 48 (n, %)** | Coagulation Defects and Other Specified Hematological Disorders | 48,403 | 8.60% | 32,052 | 9.60% |
| **HCC Category 51 (n, %)** | Dementia With Complications | 9,840 | 1.80% | 13,719 | 4.10% |
| **HCC Category 52 (n, %)** | Dementia Without Complication | 47,244 | 8.40% | 30,559 | 9.20% |

| Variable | Description | 2022-2023 | | 2024 | |
|---|---|---|---|---|---|
| **HCC Category 54 (n, %)** | Drug/Alcohol Psychosis | 8,804 | 1.60% | 5,725 | 1.70% |
| **HCC Category 55 (n, %)** | Drug/Alcohol Dependence | 100,462 | 17.90% | 62,292 | 18.70% |
| **HCC Category 56 (n, %)** | Major Depressive, Bipolar, and Paranoid Disorders | 27,419 | 4.90% | 16,788 | 5.00% |
| **HCC Category 57 (n, %)** | Schizophrenia | 17,727 | 3.20% | 10,571 | 3.20% |
| **HCC Category 58 (n, %)** | Reactive and Unspecified Psychosis | 7,878 | 1.40% | 4,895 | 1.50% |
| **HCC Category 59 (n, %)** | Personality Disorders | 157,506 | 28.10% | 98,736 | 29.70% |
| **HCC Category 60 (n, %)** | Eating Disorders | 12,481 | 2.20% | 8,163 | 2.50% |
| **HCC Category 70 (n, %)** | Quadriplegia | 4,871 | 0.90% | 3,176 | 1.00% |
| **HCC Category 71 (n, %)** | Paraplegia | 4,465 | 0.80% | 3,015 | 0.90% |
| **HCC Category 72 (n, %)** | Spinal Cord Disorders/Injuries | 10,292 | 1.80% | 7,411 | 2.20% |
| **HCC Category 73 (n, %)** | Amyotrophic Lateral Sclerosis and Other Motor Neuron Disease | 1,195 | 0.20% | 746 | 0.20% |
| **HCC Category 74 (n, %)** | Cerebral Palsy | 78 | 0.00% | 56 | 0.00% |
| **HCC Category 75 (n, %)** | Myasthenia Gravis/Myoneural Disorders, Inflammatory and Toxic Neuropathy | 7,625 | 1.40% | 5,411 | 1.60% |
| **HCC Category 76 (n, %)** | Muscular Dystrophy | 306 | 0.10% | 190 | 0.10% |
| **HCC Category 77 (n, %)** | Multiple Sclerosis | 3,454 | 0.60% | 2,043 | 0.60% |
| **HCC Category 78 (n, %)** | Parkinson's and Huntington's Diseases | 14,061 | 2.50% | 9,787 | 2.90% |
| **HCC Category 79 (n, %)** | Seizure Disorders and Convulsions | 25,982 | 4.60% | 16,677 | 5.00% |
| **HCC Category 80 (n, %)** | Coma, Brain Compression/Anoxic Damage | 2,081 | 0.40% | 1,251 | 0.40% |
| **HCC Category 82 (n, %)** | Respirator Dependence/Tracheostomy Status | 3,822 | 0.70% | 2,505 | 0.80% |
| **HCC Category 83 (n, %)** | Respiratory Arrest | 181 | 0.00% | 86 | 0.00% |
| **HCC Category 84 (n, %)** | Cardio-Respiratory Failure and Shock | 81,469 | 14.50% | 52,170 | 15.70% |
| **HCC Category 85 (n, %)** | Congestive Heart Failure | 144,068 | 25.70% | 96,211 | 28.90% |
| **HCC Category 86 (n, %)** | Acute Myocardial Infarction | 37,830 | 6.80% | 24,963 | 7.50% |
| **HCC Category 87 (n, %)** | Unstable Angina and Other Acute Ischemic Heart Disease | 22,734 | 4.10% | 13,579 | 4.10% |
| **HCC Category 88 (n, %)** | Angina Pectoris | 47,039 | 8.40% | 29,949 | 9.00% |
| **HCC Category 96 (n, %)** | Specified Heart Arrhythmias | 137,660 | 24.60% | 93,003 | 27.90% |
| **HCC Category 99 (n, %)** | Cerebral Hemorrhage | 5,012 | 0.90% | 3,437 | 1.00% |
| **HCC Category 100 (n, %)** | Ischemic or Unspecified Stroke | 43,907 | 7.80% | 29,184 | 8.80% |
| **HCC Category 103 (n, %)** | Hemiplegia/Hemiparesis | 18,635 | 3.30% | 12,417 | 3.70% |
| **HCC Category 104 (n, %)** | Monoplegia, Other Paralytic Syndromes | 3,098 | 0.60% | 2,030 | 0.60% |
| **HCC Category 106 (n, %)** | Atherosclerosis of the Extremities with Ulceration or Gangrene | 11,530 | 2.10% | 7,648 | 2.30% |
| **HCC Category 107 (n, %)** | Vascular Disease with Complications | 33,206 | 5.90% | 22,385 | 6.70% |
| **HCC Category 108 (n, %)** | Vascular Disease | 115,377 | 20.60% | 81,912 | 24.60% |

| Variable | Description | 2022-2023 | | 2024 | |
|---|---|---|---|---|---|
| **HCC Category 110 (n, %)** | Cystic Fibrosis | 55 | 0.00% | 24 | 0.00% |
| **HCC Category 111 (n, %)** | Chronic Obstructive Pulmonary Disease | 149,990 | 26.80% | 94,383 | 28.40% |
| **HCC Category 112 (n, %)** | Fibrosis of Lung and Other Chronic Lung Disorders | 22,068 | 3.90% | 15,974 | 4.80% |
| **HCC Category 114 (n, %)** | Aspiration and Specified Bacterial Pneumonias | 16,969 | 3.00% | 12,323 | 3.70% |
| **HCC Category 115 (n, %)** | Pneumococcal Pneumonia, Empyema, Lung Abscess | 10,339 | 1.80% | 7,503 | 2.30% |
| **HCC Category 122 (n, %)** | Proliferative Diabetic Retinopathy and Vitreous Hemorrhage | 6,824 | 1.20% | 4,671 | 1.40% |
| **HCC Category 124 (n, %)** | Exudative Macular Degeneration | 6,364 | 1.10% | 4,179 | 1.30% |
| **HCC Category 134 (n, %)** | Dialysis Status | 12,153 | 2.20% | 8,468 | 2.50% |
| **HCC Category 135 (n, %)** | Acute Renal Failure | 119,667 | 21.40% | 76,833 | 23.10% |
| **HCC Category 136 (n, %)** | Chronic Kidney Disease, Stage 5 | 18,319 | 3.30% | 12,427 | 3.70% |
| **HCC Category 137 (n, %)** | Chronic Kidney Disease, Severe (Stage 4) | 22,704 | 4.10% | 15,912 | 4.80% |
| **HCC Category 138 (n, %)** | Chronic Kidney Disease, Moderate (Stage 3) | 87,302 | 15.60% | 60,632 | 18.20% |
| **HCC Category 157 (n, %)** | Pressure Ulcer of Skin with Necrosis Through to Muscle, Tendon, or Bone | 3,319 | 0.60% | 2,297 | 0.70% |
| **HCC Category 158 (n, %)** | Pressure Ulcer of Skin with Full Thickness Skin Loss | 8,857 | 1.60% | 6,453 | 1.90% |
| **HCC Category 159 (n, %)** | Major Skin Burn or Condition | 9,531 | 1.70% | 6,922 | 2.10% |
| **HCC Category 161 (n, %)** | Chronic Ulcer of Skin, Except Pressure | 34,731 | 6.20% | 23,726 | 7.10% |
| **HCC Category 162 (n, %)** | Severe Skin Burn or Condition | 152 | 0.00% | 117 | 0.00% |
| **HCC Category 166 (n, %)** | Severe Head Injury | 174 | 0.00% | 101 | 0.00% |
| **HCC Category 167 (n, %)** | Major Head Injury | 7,006 | 1.30% | 4,809 | 1.40% |
| **HCC Category 169 (n, %)** | Vertebral Fractures without Spinal Cord Injury | 7,642 | 1.40% | 5,511 | 1.70% |
| **HCC Category 170 (n, %)** | Hip Fracture/Dislocation | 6,066 | 1.10% | 4,143 | 1.20% |
| **HCC Category 173 (n, %)** | Traumatic Amputations and Complications | 2,010 | 0.40% | 1,299 | 0.40% |
| **HCC Category 176 (n, %)** | Complications of Specified Implanted Device or Graft | 21,935 | 3.90% | 16,124 | 4.80% |
| **HCC Category 186 (n, %)** | Major Organ Transplant or Replacement Status | 3,322 | 0.60% | 2,291 | 0.70% |
| **HCC Category 188 (n, %)** | Artificial Openings for Feeding or Elimination | 13,782 | 2.50% | 9,862 | 3.00% |
| **HCC Category 189 (n, %)** | Amputation Status, Lower Limb/Amputation Complications | 16,239 | 2.90% | 11,372 | 3.40% |
| **CCS Category 1 (n, %)** | Incision and excision of CNS | 426 | 0.10% | 206 | 0.10% |
| **CCS Category 2 (n, %)** | Insertion, replacement, or removal of extracranial ventricular shunt | 103 | 0.00% | 57 | 0.00% |
| **CCS Category 3 (n, %)** | Laminectomy, excision intervertebral disc | 5,332 | 1.00% | 2,759 | 0.80% |
| **CCS Category 4 (n, %)** | Diagnostic spinal tap | 1,741 | 0.30% | 936 | 0.30% |
| **CCS Category 5 (n, %)** | Insertion of catheter or spinal stimulator and injection into spinal canal | 4,552 | 0.80% | 2,503 | 0.80% |
| **CCS Category 6 (n, %)** | Decompression peripheral nerve | 249 | 0.00% | 185 | 0.10% |
| **CCS Category 7 (n, %)** | Other diagnostic nervous system procedures | 1,413 | 0.30% | 865 | 0.30% |

| Variable | Description | 2022-2023 | | 2024 | |
|---|---|---|---|---|---|
| **CCS Category 8 (n, %)** | Other non-OR or closed therapeutic nervous system procedures | 14,235 | 2.50% | 9,569 | 2.90% |
| **CCS Category 9 (n, %)** | Other OR therapeutic nervous system procedures | 1,615 | 0.30% | 1,016 | 0.30% |
| **CCS Category 10 (n, %)** | Thyroidectomy, partial or complete | 1,720 | 0.30% | 985 | 0.30% |
| **CCS Category 11 (n, %)** | Diagnostic endocrine procedures | 355 | 0.10% | 286 | 0.10% |
| **CCS Category 12 (n, %)** | Other therapeutic endocrine procedures | 1,298 | 0.20% | 718 | 0.20% |
| **CCS Category 14 (n, %)** | Glaucoma procedures | 147 | 0.00% | 94 | 0.00% |
| **CCS Category 15 (n, %)** | Lens and cataract procedures | 821 | 0.10% | 621 | 0.20% |
| **CCS Category 16 (n, %)** | Repair of retinal tear, detachment | 119 | 0.00% | 64 | 0.00% |
| **CCS Category 18 (n, %)** | Diagnostic procedures on eye | 414 | 0.10% | 150 | 0.00% |
| **CCS Category 19 (n, %)** | Other therapeutic procedures on eyelids, conjunctiva, cornea | 348 | 0.10% | 244 | 0.10% |
| **CCS Category 20 (n, %)** | Other intraocular therapeutic procedures | 366 | 0.10% | 225 | 0.10% |
| **CCS Category 25 (n, %)** | Diagnostic procedures on ear | 161 | 0.00% | 94 | 0.00% |
| **CCS Category 26 (n, %)** | Other therapeutic ear procedures | 816 | 0.10% | 508 | 0.20% |
| **CCS Category 27 (n, %)** | Control of epistaxis | 195 | 0.00% | 99 | 0.00% |
| **CCS Category 28 (n, %)** | Plastic procedures on nose | 747 | 0.10% | 476 | 0.10% |
| **CCS Category 29 (n, %)** | Oral and Dental Services | 509 | 0.10% | 328 | 0.10% |
| **CCS Category 30 (n, %)** | Tonsillectomy and/or adenoidectomy | 310 | 0.10% | 141 | 0.00% |
| **CCS Category 31 (n, %)** | Diagnostic procedures on nose, mouth and pharynx | 35,277 | 6.30% | 23,556 | 7.10% |
| **CCS Category 32 (n, %)** | Other non-OR therapeutic procedures on nose, mouth and pharynx | 11,490 | 2.10% | 8,153 | 2.40% |
| **CCS Category 33 (n, %)** | Other OR therapeutic procedures on nose, mouth and pharynx | 2,565 | 0.50% | 1,443 | 0.40% |
| **CCS Category 34 (n, %)** | Tracheostomy, temporary and permanent | 546 | 0.10% | 280 | 0.10% |
| **CCS Category 35 (n, %)** | Tracheoscopy and laryngoscopy with biopsy | 2,285 | 0.40% | 1,321 | 0.40% |
| **CCS Category 36 (n, %)** | Lobectomy or pneumonectomy | 2,924 | 0.50% | 1,764 | 0.50% |
| **CCS Category 37 (n, %)** | Diagnostic bronchoscopy and biopsy of bronchus | 3,993 | 0.70% | 2,495 | 0.70% |
| **CCS Category 38 (n, %)** | Other diagnostic procedures on lung and bronchus | 12,208 | 2.20% | 7,143 | 2.10% |
| **CCS Category 39 (n, %)** | Incision of pleura, thoracentesis, chest drainage | 4,860 | 0.90% | 2,738 | 0.80% |
| **CCS Category 40 (n, %)** | Other diagnostic procedures of respiratory tract and mediastinum | 427 | 0.10% | 239 | 0.10% |
| **CCS Category 41 (n, %)** | Other non-OR therapeutic procedures on respiratory system | 43,195 | 7.70% | 27,930 | 8.40% |
| **CCS Category 42 (n, %)** | Other OR therapeutic procedures on respiratory system | 1,513 | 0.30% | 1,004 | 0.30% |
| **CCS Category 43 (n, %)** | Heart valve procedures | 2,137 | 0.40% | 1,336 | 0.40% |
| **CCS Category 44 (n, %)** | Coronary artery bypass graft (CABG) | 2,400 | 0.40% | 1,220 | 0.40% |
| **CCS Category 45 (n, %)** | Percutaneous transluminal coronary angioplasty (PTCA) | 7,936 | 1.40% | 4,154 | 1.20% |
| **CCS Category 46 (n, %)** | Coronary thrombolysis | 84 | 0.00% | 74 | 0.00% |

| Variable | Description | 2022-2023 | | 2024 | |
|---|---|---|---|---|---|
| **CCS Category 47 (n, %)** | Diagnostic cardiac catheterization, coronary arteriography | 20,935 | 3.70% | 11,966 | 3.60% |
| **CCS Category 48 (n, %)** | Insertion, revision, replacement, removal of cardiac pacemaker or cardioverter/defibrillator | 5,563 | 1.00% | 3,504 | 1.10% |
| **CCS Category 49 (n, %)** | Other OR heart procedures | 2,268 | 0.40% | 1,474 | 0.40% |
| **CCS Category 50 (n, %)** | Extracorporeal circulation auxiliary to open heart procedures | 359 | 0.10% | 210 | 0.10% |
| **CCS Category 51 (n, %)** | Endarterectomy, vessel of head and neck | 1,985 | 0.40% | 1,168 | 0.40% |
| **CCS Category 52 (n, %)** | Aortic resection, replacement or anastomosis | 2,109 | 0.40% | 1,063 | 0.30% |
| **CCS Category 54 (n, %)** | Other vascular catheterization, not heart | 92,521 | 16.50% | 53,292 | 16.00% |
| **CCS Category 55 (n, %)** | Peripheral vascular bypass | 1,510 | 0.30% | 840 | 0.30% |
| **CCS Category 56 (n, %)** | Other vascular bypass and shunt, not heart | 173 | 0.00% | 104 | 0.00% |
| **CCS Category 57 (n, %)** | Creation, revision and removal of arteriovenous fistula or vessel-to-vessel cannula for dialysis | 1,291 | 0.20% | 915 | 0.30% |
| **CCS Category 58 (n, %)** | Hemodialysis | 9,028 | 1.60% | 5,990 | 1.80% |
| **CCS Category 59 (n, %)** | Other OR procedures on vessels of head and neck | 1,262 | 0.20% | 672 | 0.20% |
| **CCS Category 60 (n, %)** | Embolectomy and endarterectomy of lower limbs | 2,063 | 0.40% | 1,160 | 0.30% |
| **CCS Category 61 (n, %)** | Other OR procedures on vessels other than head and neck | 11,647 | 2.10% | 6,682 | 2.00% |
| **CCS Category 62 (n, %)** | Other diagnostic cardiovascular procedures | 14,761 | 2.60% | 8,973 | 2.70% |
| **CCS Category 63 (n, %)** | Other non-OR therapeutic cardiovascular procedures | 7,023 | 1.30% | 4,707 | 1.40% |
| **CCS Category 64 (n, %)** | Bone marrow transplant | 145 | 0.00% | 68 | 0.00% |
| **CCS Category 65 (n, %)** | Bone marrow biopsy | 977 | 0.20% | 490 | 0.10% |
| **CCS Category 66 (n, %)** | Procedures on spleen | 75 | 0.00% | 34 | 0.00% |
| **CCS Category 67 (n, %)** | Other therapeutic procedures, hemic and lymphatic system | 7,416 | 1.30% | 4,065 | 1.20% |
| **CCS Category 68 (n, %)** | Injection or ligation of esophageal varices | 333 | 0.10% | 191 | 0.10% |
| **CCS Category 69 (n, %)** | Esophageal dilatation | 799 | 0.10% | 518 | 0.20% |
| **CCS Category 70 (n, %)** | Upper gastrointestinal endoscopy, biopsy | 16,689 | 3.00% | 9,801 | 2.90% |
| **CCS Category 71 (n, %)** | Gastrostomy, temporary and permanent | 1,742 | 0.30% | 945 | 0.30% |
| **CCS Category 72 (n, %)** | Colostomy, temporary and permanent | 253 | 0.00% | 144 | 0.00% |
| **CCS Category 73 (n, %)** | Ileostomy and other enterostomy | 526 | 0.10% | 285 | 0.10% |
| **CCS Category 74 (n, %)** | Gastrectomy, partial and total | 282 | 0.10% | 152 | 0.00% |
| **CCS Category 75 (n, %)** | Small bowel resection | 761 | 0.10% | 432 | 0.10% |
| **CCS Category 76 (n, %)** | Colonoscopy and biopsy | 9,586 | 1.70% | 5,947 | 1.80% |
| **CCS Category 77 (n, %)** | Proctoscopy and anorectal biopsy | 2,163 | 0.40% | 1,297 | 0.40% |
| **CCS Category 78 (n, %)** | Colorectal resection | 3,700 | 0.70% | 2,032 | 0.60% |
| **CCS Category 80 (n, %)** | Appendectomy | 2,570 | 0.50% | 1,411 | 0.40% |

| Variable | Description | 2022-2023 | | 2024 | |
|---|---|---|---|---|---|
| **CCS Category 81 (n, %)** | Hemorrhoid procedures | 258 | 0.00% | 156 | 0.00% |
| **CCS Category 82 (n, %)** | Endoscopic retrograde cannulation of pancreas (ERCP) | 1,787 | 0.30% | 1,010 | 0.30% |
| **CCS Category 83 (n, %)** | Biopsy of liver | 1,315 | 0.20% | 702 | 0.20% |
| **CCS Category 84 (n, %)** | Cholecystectomy and common duct exploration | 3,993 | 0.70% | 2,269 | 0.70% |
| **CCS Category 85 (n, %)** | Inguinal and femoral hernia repair | 2,343 | 0.40% | 1,505 | 0.50% |
| **CCS Category 86 (n, %)** | Other hernia repair | 2,633 | 0.50% | 573 | 0.20% |
| **CCS Category 87 (n, %)** | Laparoscopy | 767 | 0.10% | 426 | 0.10% |
| **CCS Category 88 (n, %)** | Abdominal paracentesis | 3,353 | 0.60% | 1,872 | 0.60% |
| **CCS Category 89 (n, %)** | Exploratory laparotomy | 363 | 0.10% | 192 | 0.10% |
| **CCS Category 90 (n, %)** | Excision, lysis peritoneal adhesions | 266 | 0.00% | 143 | 0.00% |
| **CCS Category 91 (n, %)** | Peritoneal dialysis | 5,791 | 1.00% | 4,473 | 1.30% |
| **CCS Category 92 (n, %)** | Other bowel diagnostic procedures | 3,835 | 0.70% | 2,248 | 0.70% |
| **CCS Category 93 (n, %)** | Other non-OR upper GI therapeutic procedures | 1,161 | 0.20% | 748 | 0.20% |
| **CCS Category 94 (n, %)** | Other OR upper GI therapeutic procedures | 975 | 0.20% | 590 | 0.20% |
| **CCS Category 95 (n, %)** | Other non-OR lower GI therapeutic procedures | 1,606 | 0.30% | 948 | 0.30% |
| **CCS Category 96 (n, %)** | Other OR lower GI therapeutic procedures | 1,650 | 0.30% | 1,079 | 0.30% |
| **CCS Category 97 (n, %)** | Other gastrointestinal diagnostic procedures | 2,931 | 0.50% | 1,789 | 0.50% |
| **CCS Category 98 (n, %)** | Other non-OR gastrointestinal therapeutic procedures | 4,804 | 0.90% | 2,858 | 0.90% |
| **CCS Category 99 (n, %)** | Other OR gastrointestinal therapeutic procedures | 2,595 | 0.50% | 1,383 | 0.40% |
| **CCS Category 100 (n, %)** | Endoscopy and endoscopic biopsy of the urinary tract | 1,954 | 0.30% | 1,155 | 0.30% |
| **CCS Category 101 (n, %)** | Transurethral excision, drainage, or removal urinary obstruction | 2,600 | 0.50% | 1,576 | 0.50% |
| **CCS Category 102 (n, %)** | Ureteral catheterization | 3,991 | 0.70% | 2,321 | 0.70% |
| **CCS Category 103 (n, %)** | Nephrotomy and nephrostomy | 1,705 | 0.30% | 1,046 | 0.30% |
| **CCS Category 104 (n, %)** | Nephrectomy, partial or complete | 2,551 | 0.50% | 1,428 | 0.40% |
| **CCS Category 105 (n, %)** | Kidney transplant | 406 | 0.10% | 219 | 0.10% |
| **CCS Category 106 (n, %)** | Genitourinary incontinence procedures | 400 | 0.10% | 211 | 0.10% |
| **CCS Category 107 (n, %)** | Extracorporeal lithotripsy, urinary | 1,285 | 0.20% | 849 | 0.30% |
| **CCS Category 108 (n, %)** | Indwelling catheter | 6,197 | 1.10% | 3,577 | 1.10% |
| **CCS Category 109 (n, %)** | Procedures on the urethra | 652 | 0.10% | 352 | 0.10% |
| **CCS Category 110 (n, %)** | Other diagnostic procedures of urinary tract | 955 | 0.20% | 536 | 0.20% |
| **CCS Category 111 (n, %)** | Other non-OR therapeutic procedures of urinary tract | 2,520 | 0.50% | 1,474 | 0.40% |
| **CCS Category 112 (n, %)** | Other OR therapeutic procedures of urinary tract | 2,196 | 0.40% | 1,288 | 0.40% |
| **CCS Category 113 (n, %)** | Transurethral resection of prostate (TURP) | 3,940 | 0.70% | 2,294 | 0.70% |

| Variable | Description | 2022-2023 | | 2024 | |
|---|---|---|---|---|---|
| **CCS Category 114 (n, %)** | Open prostatectomy | 3,627 | 0.60% | 1,628 | 0.50% |
| **CCS Category 116 (n, %)** | Diagnostic procedures, male genital | 441 | 0.10% | 311 | 0.10% |
| **CCS Category 117 (n, %)** | Other non-OR therapeutic procedures, male genital | 320 | 0.10% | 147 | 0.00% |
| **CCS Category 118 (n, %)** | Other OR therapeutic procedures, male genital | 2,501 | 0.40% | 1,360 | 0.40% |
| **CCS Category 124 (n, %)** | Hysterectomy, abdominal and vaginal | 1,495 | 0.30% | 700 | 0.20% |
| **CCS Category 130 (n, %)** | Other diagnostic procedures, female organs | 180 | 0.00% | 121 | 0.00% |
| **CCS Category 132 (n, %)** | Other OR therapeutic procedures, female organs | 192 | 0.00% | 113 | 0.00% |
| **CCS Category 142 (n, %)** | Partial excision bone | 1,082 | 0.20% | 696 | 0.20% |
| **CCS Category 143 (n, %)** | Bunionectomy or repair of toe deformities | 123 | 0.00% | 87 | 0.00% |
| **CCS Category 144 (n, %)** | Treatment, facial fracture or dislocation | 284 | 0.10% | 160 | 0.00% |
| **CCS Category 145 (n, %)** | Treatment, fracture or dislocation of radius and ulna | 220 | 0.00% | 90 | 0.00% |
| **CCS Category 146 (n, %)** | Treatment, fracture or dislocation of hip and femur | 1,560 | 0.30% | 944 | 0.30% |
| **CCS Category 147 (n, %)** | Treatment, fracture or dislocation of lower extremity (other than hip or femur) | 882 | 0.20% | 450 | 0.10% |
| **CCS Category 148 (n, %)** | Other fracture and dislocation procedure | 529 | 0.10% | 355 | 0.10% |
| **CCS Category 149 (n, %)** | Arthroscopy | 144 | 0.00% | 102 | 0.00% |
| **CCS Category 151 (n, %)** | Excision of semilunar cartilage of knee | 120 | 0.00% | 74 | 0.00% |
| **CCS Category 152 (n, %)** | Arthroplasty knee | 13,718 | 2.40% | 8,553 | 2.60% |
| **CCS Category 153 (n, %)** | Hip replacement, total and partial | 7,089 | 1.30% | 4,226 | 1.30% |
| **CCS Category 154 (n, %)** | Arthroplasty other than hip or knee | 3,030 | 0.50% | 1,877 | 0.60% |
| **CCS Category 155 (n, %)** | Arthrocentesis | 1,612 | 0.30% | 970 | 0.30% |
| **CCS Category 156 (n, %)** | Injections and aspirations of muscles, tendons, bursa, joints and soft tissue | 882 | 0.20% | 445 | 0.10% |
| **CCS Category 157 (n, %)** | Amputation of lower extremity | 4,005 | 0.70% | 2,327 | 0.70% |
| **CCS Category 158 (n, %)** | Spinal fusion | 4,486 | 0.80% | 2,552 | 0.80% |
| **CCS Category 159 (n, %)** | Other diagnostic procedures on musculoskeletal system | 2,559 | 0.50% | 1,484 | 0.40% |
| **CCS Category 160 (n, %)** | Other therapeutic procedures on muscles and tendons | 2,472 | 0.40% | 1,690 | 0.50% |
| **CCS Category 161 (n, %)** | Other OR therapeutic procedures on bone | 4,643 | 0.80% | 2,569 | 0.80% |
| **CCS Category 162 (n, %)** | Other OR therapeutic procedures on joints | 1,270 | 0.20% | 825 | 0.20% |
| **CCS Category 163 (n, %)** | Other non-OR therapeutic procedures on musculoskeletal system | 723 | 0.10% | 422 | 0.10% |
| **CCS Category 164 (n, %)** | Other OR therapeutic procedures on musculoskeletal system | 699 | 0.10% | 707 | 0.20% |
| **CCS Category 167 (n, %)** | Mastectomy | 463 | 0.10% | 287 | 0.10% |
| **CCS Category 168 (n, %)** | Incision and drainage, skin and subcutaneous tissue | 3,312 | 0.60% | 1,817 | 0.50% |
| **CCS Category 169 (n, %)** | Debridement of wound, infection or burn | 4,357 | 0.80% | 2,751 | 0.80% |
| **CCS Category 170 (n, %)** | Excision of skin lesion | 3,144 | 0.60% | 2,016 | 0.60% |

| Variable | Description | 2022-2023 | | 2024 | |
|---|---|---|---|---|---|
| **CCS Category 171 (n, %)** | Suture of skin and subcutaneous tissue | 1,049 | 0.20% | 624 | 0.20% |
| **CCS Category 172 (n, %)** | Skin graft | 1,650 | 0.30% | 1,044 | 0.30% |
| **CCS Category 173 (n, %)** | Other diagnostic procedures on skin and subcutaneous tissue | 2,517 | 0.40% | 1,400 | 0.40% |
| **CCS Category 174 (n, %)** | Other non-OR therapeutic procedures on skin and breast | 6,494 | 1.20% | 4,343 | 1.30% |
| **CCS Category 175 (n, %)** | Other OR therapeutic procedures on skin and breast | 1,990 | 0.40% | 1,386 | 0.40% |
| **CCS Category 176 (n, %)** | Other organ transplantation | 42 | 0.00% | 20 | 0.00% |
| **CCS Category 177 (n, %)** | Computerized axial tomography (CT) scan head | 103,752 | 18.50% | 62,742 | 18.90% |
| **CCS Category 178 (n, %)** | CT scan chest | 91,308 | 16.30% | 56,054 | 16.80% |
| **CCS Category 179 (n, %)** | CT scan abdomen | 113,535 | 20.30% | 72,029 | 21.60% |
| **CCS Category 180 (n, %)** | Other CT scan | 43,280 | 7.70% | 26,979 | 8.10% |
| **CCS Category 181 (n, %)** | Myelogram | 242 | 0.00% | 151 | 0.00% |
| **CCS Category 182 (n, %)** | Mammography | 186 | 0.00% | 120 | 0.00% |
| **CCS Category 183 (n, %)** | Routine chest X-ray | 307,854 | 55.00% | 174,529 | 52.40% |
| **CCS Category 184 (n, %)** | Intraoperative cholangiogram | 311 | 0.10% | 154 | 0.00% |
| **CCS Category 185 (n, %)** | Upper gastrointestinal X-ray | 7,223 | 1.30% | 4,784 | 1.40% |
| **CCS Category 186 (n, %)** | Lower gastrointestinal X-ray | 119 | 0.00% | 53 | 0.00% |
| **CCS Category 187 (n, %)** | Intravenous pyelogram | 438 | 0.10% | 230 | 0.10% |
| **CCS Category 189 (n, %)** | Contrast aortogram | 4,956 | 0.90% | 3,109 | 0.90% |
| **CCS Category 190 (n, %)** | Contrast arteriogram of femoral and lower extremity arteries | 1,568 | 0.30% | 942 | 0.30% |
| **CCS Category 191 (n, %)** | Arterio- or venogram (not heart and head) | 21,127 | 3.80% | 14,294 | 4.30% |
| **CCS Category 192 (n, %)** | Diagnostic ultrasound of head and neck | 12,818 | 2.30% | 7,105 | 2.10% |
| **CCS Category 193 (n, %)** | Diagnostic ultrasound of heart (echocardiogram) | 119,563 | 21.40% | 71,741 | 21.60% |
| **CCS Category 194 (n, %)** | Diagnostic ultrasound of gastrointestinal tract | 1,109 | 0.20% | 498 | 0.10% |
| **CCS Category 195 (n, %)** | Diagnostic ultrasound of urinary tract | 3,023 | 0.50% | 1,108 | 0.30% |
| **CCS Category 196 (n, %)** | Diagnostic ultrasound of abdomen or retroperitoneum | 47,507 | 8.50% | 28,038 | 8.40% |
| **CCS Category 197 (n, %)** | Other diagnostic ultrasound | 70,231 | 12.50% | 44,423 | 13.30% |
| **CCS Category 198 (n, %)** | Magnetic resonance imaging | 52,656 | 9.40% | 32,202 | 9.70% |
| **CCS Category 199 (n, %)** | Electroencephalogram (EEG) | 5,563 | 1.00% | 3,431 | 1.00% |
| **CCS Category 200 (n, %)** | Nonoperative urinary system measurements | 90,426 | 16.10% | 54,169 | 16.30% |
| **CCS Category 201 (n, %)** | Cardiac stress tests | 14,669 | 2.60% | 7,595 | 2.30% |
| **CCS Category 202 (n, %)** | Electrocardiogram | 328,321 | 58.60% | 203,094 | 61.00% |
| **CCS Category 203 (n, %)** | Electrographic cardiac monitoring | 30,032 | 5.40% | 17,894 | 5.40% |
| **CCS Category 204 (n, %)** | Swan-Ganz catheterization for monitoring | 2,317 | 0.40% | 1,378 | 0.40% |

| Variable | Description | 2022-2023 | | 2024 | |
|---|---|---|---|---|---|
| **CCS Category 205 (n, %)** | Arterial blood gases | 64,291 | 11.50% | 42,134 | 12.70% |
| **CCS Category 206 (n, %)** | Microscopic examination (bacterial smear, culture, toxicology) | 318,815 | 56.90% | 165,626 | 49.80% |
| **CCS Category 207 (n, %)** | Radioisotope bone scan | 1,014 | 0.20% | 446 | 0.10% |
| **CCS Category 208 (n, %)** | Radioisotope pulmonary scan | 2,460 | 0.40% | 1,179 | 0.40% |
| **CCS Category 209 (n, %)** | Radioisotope scan and function studies | 21,952 | 3.90% | 11,739 | 3.50% |
| **CCS Category 210 (n, %)** | Other radioisotope scan | 5,473 | 1.00% | 2,575 | 0.80% |
| **CCS Category 211 (n, %)** | Therapeutic radiology | 1,424 | 0.30% | 887 | 0.30% |
| **CCS Category 212 (n, %)** | Diagnostic physical, occupational, and speech therapy | 183,489 | 32.80% | 115,205 | 34.60% |
| **CCS Category 213 (n, %)** | Physical, occupational, and speech therapy exercises; manipulation; and other procedures | 125,927 | 22.50% | 79,798 | 24.00% |
| **CCS Category 214 (n, %)** | Traction, splints, and other wound care | 8,288 | 1.50% | 4,517 | 1.40% |
| **CCS Category 215 (n, %)** | Other physical, occupational, and speech therapy and rehabilitation | 32,156 | 5.70% | 17,643 | 5.30% |
| **CCS Category 216 (n, %)** | Respiratory intubation and mechanical ventilation | 31,144 | 5.60% | 18,914 | 5.70% |
| **CCS Category 217 (n, %)** | Other respiratory therapy | 26,289 | 4.70% | 17,676 | 5.30% |
| **CCS Category 218 (n, %)** | Psychological and psychiatric evaluation and therapy | 136,155 | 24.30% | 95,819 | 28.80% |
| **CCS Category 219 (n, %)** | Alcohol and drug management, treatment, and rehabilitation | 23,387 | 4.20% | 15,220 | 4.60% |
| **CCS Category 220 (n, %)** | Ophthalmologic and otologic diagnosis and treatment | 10,698 | 1.90% | 6,455 | 1.90% |
| **CCS Category 221 (n, %)** | Nasogastric tube | 650 | 0.10% | 364 | 0.10% |
| **CCS Category 222 (n, %)** | Blood and blood product transfusion | 22,261 | 4.00% | 12,778 | 3.80% |
| **CCS Category 223 (n, %)** | Enteral and parenteral nutrition | 1,020 | 0.20% | 873 | 0.30% |
| **CCS Category 224 (n, %)** | Cancer chemotherapy | 2,757 | 0.50% | 1,625 | 0.50% |
| **CCS Category 225 (n, %)** | Conversion of cardiac rhythm | 5,578 | 1.00% | 3,781 | 1.10% |
| **CCS Category 226 (n, %)** | Other diagnostic radiology and related techniques | 145,224 | 25.90% | 87,717 | 26.40% |
| **CCS Category 227 (n, %)** | Consultation, evaluation, and preventative care | 535,385 | 95.60% | 315,164 | 94.70% |
| **CCS Category 228 (n, %)** | Prophylactic vaccinations and inoculations | 24,422 | 4.40% | 9,466 | 2.80% |
| **CCS Category 229 (n, %)** | Nonoperative removal of foreign body | 642 | 0.10% | 410 | 0.10% |
| **CCS Category 231 (n, %)** | Other therapeutic procedures | 77,754 | 13.90% | 45,743 | 13.70% |
| **CCS Category 232 (n, %)** | Anesthesia | 97,748 | 17.50% | 58,114 | 17.50% |
| **CCS Category 233 (n, %)** | Laboratory - Chemistry and Hematology | 469,817 | 83.90% | 276,428 | 83.10% |
| **CCS Category 234 (n, %)** | Pathology | 113,281 | 20.20% | 65,460 | 19.70% |
| **CCS Category 235 (n, %)** | Other Laboratory | 298,174 | 53.20% | 155,960 | 46.90% |
| **CCS Category 236 (n, %)** | Nonhospital-based care (e.g., home health care, hospice) | 251,307 | 44.90% | 156,143 | 46.90% |
| **CCS Category 237 (n, %)** | Ancillary Services | 324,703 | 58.00% | 202,253 | 60.80% |
| **CCS Category 239 (n, %)** | Transportation - patient, provider, equipment | 1,111 | 0.20% | 608 | 0.20% |

| Variable | Description | 2022-2023 | | 2024 | |
|---|---|---|---|---|---|
| **CCS Category 240 (n, %)** | Medications (Injections, infusions and other forms) | 87,987 | 15.70% | 50,163 | 15.10% |
| **CCS Category 241 (n, %)** | Visual aids and other optical supplies | 853 | 0.20% | 621 | 0.20% |
| **CCS Category 242 (n, %)** | Hearing devices and audiology supplies | 1,760 | 0.30% | 1,402 | 0.40% |
| **CCS Category 243 (n, %)** | DME and supplies | 98,602 | 17.60% | 61,433 | 18.50% |
| **CCS Category 244 (n, %)** | Gastric bypass and volume reduction | 1,697 | 0.30% | 986 | 0.30% |
| **CCS Category 245 (n, %)** | Telehealth (includes telephone calls, online communication, remote monitoring and surveillance) | 260,860 | 46.60% | 147,187 | 44.20% |

Abbreviations: AIDS: Acquired Immunodeficiency Syndrome; CCS: Clinical Classification Software; CT: Computed Tomography; DME: Durable Medical Equipment; GI: Gastrointestinal; HCC: Hierarchical Condition Categories; HIV: Human Immunodeficiency Virus; ICU: Intensive Care Unit; IQR: Interquartile Range; OR: Operating Room; 95%CI: 95% confidence interval
