# Supplementary material for: DASC-LOT Framework and S3 Metric: A Novel Evaluation and Benchmarking Method to Assess Initiation, Duration, and Spectrum of Antimicrobial Usage at Inpatient Hospital Settings
Source: Clin Infect Dis. 2026 Jun 25;83(3):e475–83. doi: 10.1093/cid/ciag389 (PMC13644869; doi:10.1093/cid/ciag389)
Supplement: Supplementary file 1 [file ciag389_supplementary_data.zip › Supplementary Table 2 20260304.pdf]

Supplementary Table 2. Fixed-Effect Covariates for Risk-Adjustments for Three Components

Variable	Description	Odds Ratio (Component 1)	p-value (Component 1)	Rate Ratio (Component 2)	p-value (Component 2)	Rate Ratio (Component 3)	p-value (Component 3)
**Age**	<45					(Reference)	
	45-54					0.99 (0.99-1.00)	
	55-64					0.98 (0.97-0.98)	
	65-74					0.97 (0.97-0.98)	<0.001
	75-84					0.97 (0.97-0.98)	
	>=85					0.97 (0.96-0.97)	
**CCS Category 1**	Incision and excision of CNS					0.69 (0.66-0.71)	
**CCS Category 3**	Laminectomy, excision intervertebral disc	2.10 (2.00-2.21)	<0.001			0.60 (0.59-0.61)	
**CCS Category 5**	Insertion of catheter or spinal stimulator and injection into spinal canal			0.85 (0.83-0.87)	<0.001		
**CCS Category 7**	Other diagnostic nervous system procedures			0.92 (0.90-0.95)	<0.001		
**CCS Category 8**	Other non-OR or closed therapeutic nervous system procedures	1.31 (1.26-1.35)	<0.001			0.94 (0.93-0.94)	<0.001
**CCS Category 10**	Thyroidectomy, partial or complete					0.57 (0.54-0.60)	<0.001
**CCS Category 28**	Plastic procedures on nose					0.68 (0.66-0.71)	<0.001
**CCS Category 31**	Diagnostic procedures on nose, mouth and pharynx	1.54 (1.51-1.56)	<0.001			1.04 (1.04-1.04)	<0.001
**CCS Category 32**	Other non-OR therapeutic procedures on nose, mouth and pharynx			0.98 (0.98-0.99)	<0.001		
**CCS Category 33**	Other OR therapeutic procedures on nose, mouth and pharynx			1.17 (1.13-1.21)	<0.001		
**CCS Category 36**	Lobectomy or pneumonectomy			0.75 (0.72-0.78)	<0.001	0.61 (0.59-0.62)	<0.001
**CCS Category 37**	Diagnostic bronchoscopy and biopsy of bronchus			1.08 (1.07-1.09)	<0.001	1.09 (1.08-1.10)	<0.001
**CCS Category 39**	Incision of pleura, thoracentesis, chest drainage	0.80 (0.77-0.83)	<0.001				
**CCS Category 41**	Other non-OR therapeutic procedures on respiratory system	1.37 (1.35-1.39)	<0.001			1.01 (1.01-1.02)	<0.001
**CCS Category 43**	Heart valve procedures			0.83 (0.80-0.85)	<0.001	0.80 (0.79-0.82)	<0.001
**CCS Category 44**	Coronary artery bypass graft (CABG)			0.64 (0.62-0.65)	<0.001	0.62 (0.61-0.63)	<0.001
**CCS Category 45**	Percutaneous transluminal coronary angioplasty (PTCA)	0.54 (0.51-0.58)	<0.001				

Variable	Description	Odds Ratio (Component 1)	p-value (Component 1)	Rate Ratio (Component 2)	p-value (Component 2)	Rate Ratio (Component 3)	p-value (Component 3)
**CCS Category 47**	Diagnostic cardiac catheterization, coronary arteriography	0.40 (0.39-0.42)	<0.001	0.91 (0.90-0.93)	<0.001		
**CCS Category 48**	Insertion, revision, replacement, removal of cardiac pacemaker or cardioverter/defibrillator	4.51 (4.33-4.70)	<0.001	0.85 (0.84-0.87)	<0.001	0.66 (0.65-0.67)	<0.001
**CCS Category 51**	Endarterectomy, vessel of head and neck					0.50 (0.49-0.52)	<0.001
**CCS Category 52**	Aortic resection, replacement or anastomosis					0.65 (0.63-0.66)	<0.001
**CCS Category 54**	Other vascular catheterization, not heart	1.15 (1.13-1.16)	<0.001	1.07 (1.06-1.07)	<0.001		
**CCS Category 58**	Hemodialysis			0.96 (0.95-0.98)	<0.001		
**CCS Category 60**	Embolectomy and endarterectomy of lower limbs					0.83 (0.81-0.84)	<0.001
**CCS Category 62**	Other diagnostic cardiovascular procedures	1.22 (1.19-1.26)	<0.001	1.05 (1.04-1.06)	<0.001	1.04 (1.03-1.04)	<0.001
**CCS Category 64**	Bone marrow transplant			1.28 (1.22-1.35)	<0.001		
**CCS Category 65**	Bone marrow biopsy					1.03 (1.01-1.06)	<0.01
**CCS Category 67**	Other therapeutic procedures, hemic and lymphatic system			0.91 (0.89-0.93)	<0.001	0.88 (0.87-0.89)	<0.001
**CCS Category 68**	Injection or ligation of esophageal varices					0.86 (0.83-0.88)	<0.001
**CCS Category 70**	Upper gastrointestinal endoscopy, biopsy	0.63 (0.62-0.65)	<0.001	0.94 (0.93-0.94)	<0.001	0.95 (0.94-0.96)	<0.001
**CCS Category 71**	Gastrostomy, temporary and permanent			0.90 (0.89-0.92)	<0.001		
**CCS Category 76**	Colonoscopy and biopsy	0.58 (0.55-0.60)	<0.001	0.94 (0.93-0.96)	<0.001		
**CCS Category 78**	Colorectal resection			0.87 (0.86-0.89)	<0.001		
**CCS Category 80**	Appendectomy					1.15 (1.13-1.17)	<0.001
**CCS Category 82**	Endoscopic retrograde cannulation of pancreas (ERCP)					1.11 (1.09-1.12)	<0.001
**CCS Category 83**	Biopsy of liver			0.93 (0.90-0.96)	<0.001		
**CCS Category 84**	Cholecystectomy and common duct exploration	2.26 (2.15-2.37)	<0.001			1.13 (1.12-1.15)	<0.001
**CCS Category 88**	Abdominal paracentesis	1.59 (1.53-1.66)	<0.001				
**CCS Category 91**	Peritoneal dialysis	0.85 (0.82-0.88)	<0.001			0.98 (0.97-0.99)	<0.01
**CCS Category 92**	Other bowel diagnostic procedures	0.83 (0.79-0.88)	<0.001				

Variable	Description	Odds Ratio (Component 1)	p-value (Component 1)	Rate Ratio (Component 2)	p-value (Component 2)	Rate Ratio (Component 3)	p-value (Component 3)
**CCS Category 96**	Other OR lower GI therapeutic procedures					1.12 (1.10-1.14)	<0.001
**CCS Category 98**	Other non-OR gastrointestinal therapeutic procedures	1.99 (1.92-2.07)	<0.001	1.14 (1.13-1.15)	<0.001	1.11 (1.10-1.12)	<0.001
**CCS Category 99**	Other OR gastrointestinal therapeutic procedures			1.05 (1.03-1.07)	<0.001	1.06 (1.04-1.07)	<0.001
**CCS Category 102**	Ureteral catheterization	1.50 (1.43-1.58)	<0.001				
**CCS Category 104**	Nephrectomy, partial or complete					0.63 (0.62-0.65)	<0.001
**CCS Category 108**	Indwelling catheter	1.50 (1.45-1.56)	<0.001				
**CCS Category 110**	Other diagnostic procedures of urinary tract			0.91 (0.87-0.94)	<0.001		
**CCS Category 111**	Other non-OR therapeutic procedures of urinary tract	1.65 (1.56-1.74)	<0.001				
**CCS Category 113**	Transurethral resection of prostate (TURP)					0.85 (0.83-0.87)	<0.001
**CCS Category 114**	Open prostatectomy					0.60 (0.59-0.61)	<0.001
**CCS Category 118**	Other OR therapeutic procedures, male genital			1.10 (1.07-1.13)	<0.001	1.20 (1.18-1.21)	<0.001
**CCS Category 142**	Partial excision bone			1.08 (1.06-1.10)	<0.001		
**CCS Category 146**	Treatment, fracture or dislocation of hip and femur			0.63 (0.61-0.65)	<0.001	0.64 (0.63-0.65)	<0.001
**CCS Category 147**	Treatment, fracture or dislocation of lower extremity (other than hip or femur)			0.69 (0.65-0.72)	<0.001	0.63 (0.61-0.65)	<0.001
**CCS Category 152**	Arthroplasty knee	15.90 (15.09-16.75)	<0.001			0.55 (0.54-0.55)	<0.001
**CCS Category 153**	Hip replacement, total and partial	11.88 (11.17-12.64)	<0.001	0.75 (0.73-0.76)	<0.001	0.54 (0.53-0.54)	<0.001
**CCS Category 154**	Arthroplasty other than hip or knee					0.55 (0.54-0.56)	<0.001
**CCS Category 157**	Amputation of lower extremity	2.11 (2.01-2.22)	<0.001	1.06 (1.05-1.07)	<0.001	1.14 (1.13-1.15)	<0.001
**CCS Category 158**	Spinal fusion	1.94 (1.84-2.05)	<0.001	0.77 (0.75-0.79)	<0.001	0.61 (0.60-0.62)	<0.001
**CCS Category 159**	Other diagnostic procedures on musculoskeletal system	1.29 (1.21-1.36)	<0.001				
**CCS Category 162**	Other OR therapeutic procedures on joints			1.15 (1.13-1.18)	<0.001		
**CCS Category 168**	Incision and drainage, skin and subcutaneous tissue	7.53 (7.03-8.07)	<0.001	1.16 (1.15-1.18)	<0.001	1.13 (1.12-1.14)	<0.001
**CCS Category 169**	Debridement of wound, infection or burn	2.63 (2.50-2.76)	<0.001	1.10 (1.09-1.11)	<0.001	1.13 (1.12-1.14)	<0.001

Variable	Description	Odds Ratio (Component 1)	p-value (Component 1)	Rate Ratio (Component 2)	p-value (Component 2)	Rate Ratio (Component 3)	p-value (Component 3)
**CCS Category 170**	Excision of skin lesion	1.26 (1.19-1.33)	<0.001				
**CCS Category 176**	Other organ transplantation					1.79 (1.69-1.90)	<0.001
**CCS Category 177**	Computerized axial tomography (CT) scan head	0.77 (0.76-0.78)	<0.001	0.98 (0.98-0.99)	<0.001		
**CCS Category 178**	CT scan chest	1.28 (1.26-1.29)	<0.001			1.08 (1.08-1.09)	<0.001
**CCS Category 179**	CT scan abdomen	1.58 (1.56-1.59)	<0.001	1.06 (1.06-1.07)	<0.001	1.06 (1.06-1.07)	<0.001
**CCS Category 180**	Other CT scan	1.09 (1.08-1.11)	<0.001			1.04 (1.03-1.04)	<0.001
**CCS Category 183**	Routine chest X-ray	1.19 (1.17-1.20)	<0.001	0.99 (0.99-0.99)	<0.001	1.06 (1.05-1.06)	<0.001
**CCS Category 189**	Contrast aortogram					1.05 (1.04-1.06)	<0.001
**CCS Category 191**	Arterio- or venogram (not heart and head)	0.61 (0.59-0.63)	<0.001				
**CCS Category 192**	Diagnostic ultrasound of head and neck	0.69 (0.67-0.72)	<0.001				
**CCS Category 193**	Diagnostic ultrasound of heart (echocardiogram)					0.99 (0.99-0.99)	<0.001
**CCS Category 195**	Diagnostic ultrasound of urinary tract	1.38 (1.31-1.46)	<0.001				
**CCS Category 196**	Diagnostic ultrasound of abdomen or retroperitoneum	1.26 (1.24-1.28)	<0.001				
**CCS Category 197**	Other diagnostic ultrasound	1.37 (1.35-1.39)	<0.001			1.01 (1.01-1.02)	<0.001
**CCS Category 198**	Magnetic resonance imaging	0.90 (0.88-0.91)	<0.001			1.03 (1.02-1.03)	<0.001
**CCS Category 200**	Nonoperative urinary system measurements	1.08 (1.07-1.10)	<0.001				
**CCS Category 201**	Cardiac stress tests	0.42 (0.40-0.43)	<0.001				
**CCS Category 202**	Electrocardiogram	0.79 (0.79-0.80)	<0.001	0.99 (0.99-0.99)	<0.001		
**CCS Category 203**	Electrographic cardiac monitoring	0.80 (0.79-0.82)	<0.001				
**CCS Category 204**	Swan-Ganz catheterization for monitoring	2.21 (2.08-2.34)	<0.001				
**CCS Category 205**	Arterial blood gases	1.22 (1.20-1.23)	<0.001			1.01 (1.01-1.01)	<0.001
**CCS Category 206**	Microscopic examination (bacterial smear, culture, toxicology)	1.78 (1.77-1.80)	<0.001	1.09 (1.09-1.10)	<0.001	1.05 (1.05-1.06)	<0.001
**CCS Category 209**	Radioisotope scan and function studies			0.98 (0.98-0.99)	<0.001		
**CCS Category 211**	Therapeutic radiology			0.89 (0.87-0.91)	<0.001		
**CCS Category 212**	Diagnostic physical, occupational, and speech therapy	1.21 (1.19-1.22)	<0.001			0.97 (0.97-0.97)	<0.001

Variable	Description	Odds Ratio (Component 1)	p-value (Component 1)	Rate Ratio (Component 2)	p-value (Component 2)	Rate Ratio (Component 3)	p-value (Component 3)
**CCS Category 213**	Physical, occupational, and speech therapy exercises; manipulation; and other procedures			0.97 (0.96-0.97)	<0.001	0.97 (0.97-0.98)	<0.001
**CCS Category 214**	Traction, splints, and other wound care	1.48 (1.44-1.52)	<0.001			1.03 (1.02-1.04)	<0.001
**CCS Category 215**	Other physical, occupational, and speech therapy and rehabilitation	0.91 (0.89-0.92)	<0.001	0.96 (0.96-0.97)	<0.001		
**CCS Category 216**	Respiratory intubation and mechanical ventilation					1.03 (1.02-1.03)	<0.001
**CCS Category 217**	Other respiratory therapy	1.56 (1.53-1.59)	<0.001				
**CCS Category 218**	Psychological and psychiatric evaluation and therapy	0.88 (0.87-0.89)	<0.001	0.98 (0.97-0.98)	<0.001		
**CCS Category 219**	Alcohol and drug management, treatment, and rehabilitation	0.58 (0.57-0.59)	<0.001			0.96 (0.95-0.97)	<0.001
**CCS Category 220**	Ophthalmologic and otologic diagnosis and treatment	0.88 (0.86-0.91)	<0.001				
**CCS Category 222**	Blood and blood product transfusion	1.08 (1.06-1.10)	<0.001				
**CCS Category 224**	Cancer chemotherapy			0.92 (0.90-0.93)	<0.001		
**CCS Category 225**	Conversion of cardiac rhythm	0.41 (0.39-0.43)	<0.001				
**CCS Category 226**	Other diagnostic radiology and related techniques	1.31 (1.30-1.33)	<0.001	1.00 (0.99-1.00)	0.11		
**CCS Category 227**	Consultation, evaluation, and preventative care	1.08 (1.06-1.10)	<0.001				
**CCS Category 228**	Prophylactic vaccinations and inoculations	0.94 (0.92-0.96)	<0.001	0.97 (0.96-0.98)	<0.001		
**CCS Category 231**	Other therapeutic procedures	1.14 (1.13-1.16)	<0.001				
**CCS Category 232**	Anesthesia	1.08 (1.07-1.10)	<0.001			0.94 (0.94-0.95)	<0.001
**CCS Category 234**	Pathology	1.11 (1.10-1.13)	<0.001				
**CCS Category 235**	Other Laboratory	0.91 (0.90-0.91)	<0.001	1.01 (1.00-1.01)	<0.01	1.01 (1.00-1.01)	<0.001
**CCS Category 236**	Nonhospital-based care (e.g., home health care, hospice)	1.09 (1.08-1.10)	<0.001	1.01 (1.01-1.01)	<0.001	1.00 (0.99-1.00)	<0.01
**CCS Category 237**	Ancillary Services	1.18 (1.17-1.19)	<0.001	1.03 (1.02-1.03)	<0.001	1.02 (1.02-1.03)	<0.001
**CCS Category 243**	DME and supplies	1.08 (1.07-1.10)	<0.001				
**CCS Category 245**	Telehealth (includes telephone calls, online communication, remote monitoring and surveillance)	1.30 (1.28-1.31)	<0.001			1.05 (1.05-1.05)	<0.001
**HCC Category 1**	HIV/AIDS	1.38 (1.34-1.43)	<0.001				

Variable	Description	Odds Ratio (Component 1)	p-value (Component 1)	Rate Ratio (Component 2)	p-value (Component 2)	Rate Ratio (Component 3)	p-value (Component 3)
**HCC Category 2**	Septicemia, Sepsis, Systemic Inflammatory Response Syndrome/Shock	3.09 (3.05-3.12)	<0.001	1.13 (1.12-1.13)	<0.001	1.11 (1.11-1.11)	<0.001
**HCC Category 6**	Opportunistic Infections	1.19 (1.15-1.23)	<0.001			1.05 (1.04-1.06)	<0.001
**HCC Category 8**	Metastatic Cancer and Acute Leukemia	0.90 (0.88-0.91)	<0.001			1.03 (1.02-1.03)	<0.001
**HCC Category 9**	Lung and Other Severe Cancers	0.92 (0.91-0.94)	<0.001			1.03 (1.02-1.03)	<0.001
**HCC Category 10**	Lymphoma and Other Cancers					1.02 (1.01-1.02)	<0.001
**HCC Category 18**	Diabetes with Chronic Complications	0.95 (0.94-0.96)	<0.001				
**HCC Category 21**	Protein-Calorie Malnutrition	0.91 (0.90-0.93)	<0.001	0.97 (0.97-0.97)	<0.001		
**HCC Category 23**	Other Significant Endocrine and Metabolic Disorders	0.88 (0.86-0.89)	<0.001	0.98 (0.97-0.98)	<0.001		
**HCC Category 27**	End-Stage Liver Disease	1.27 (1.24-1.30)	<0.001				
**HCC Category 28**	Cirrhosis of Liver					0.96 (0.95-0.96)	<0.001
**HCC Category 33**	Intestinal Obstruction/Perforation					1.03 (1.03-1.04)	<0.001
**HCC Category 34**	Chronic Pancreatitis	0.75 (0.73-0.78)	<0.001				
**HCC Category 39**	Bone/Joint/Muscle Infections/Necrosis	1.93 (1.89-1.96)	<0.001	1.16 (1.15-1.16)	<0.001	1.10 (1.09-1.10)	<0.001
**HCC Category 46**	Severe Hematological Disorders					1.05 (1.04-1.06)	<0.001
**HCC Category 47**	Disorders of Immunity	1.25 (1.23-1.27)	<0.001	1.04 (1.03-1.04)	<0.001	1.04 (1.04-1.05)	<0.001
**HCC Category 51**	Dementia With Complications	0.82 (0.80-0.84)	<0.001	0.90 (0.89-0.91)	<0.001	0.98 (0.97-0.98)	<0.001
**HCC Category 52**	Dementia Without Complication	0.89 (0.88-0.90)	<0.001	0.94 (0.93-0.94)	<0.001	0.98 (0.98-0.98)	<0.001
**HCC Category 54**	Drug/Alcohol Psychosis	0.71 (0.69-0.73)	<0.001				
**HCC Category 55**	Drug/Alcohol Dependence	0.85 (0.84-0.86)	<0.001	0.99 (0.99-0.99)	<0.001		
**HCC Category 56**	Major Depressive, Bipolar, and Paranoid Disorders	0.95 (0.94-0.97)	<0.001				
**HCC Category 57**	Schizophrenia	0.90 (0.88-0.92)	<0.001	0.97 (0.96-0.98)	<0.001		
**HCC Category 58**	Reactive and Unspecified Psychosis	0.92 (0.89-0.95)	<0.001	0.96 (0.95-0.98)	<0.001		
**HCC Category 59**	Personality Disorders	0.92 (0.91-0.93)	<0.001				
**HCC Category 60**	Eating Disorders	0.85 (0.83-0.87)	<0.001	0.96 (0.95-0.97)	<0.001		
**HCC Category 71**	Paraplegia	1.17 (1.12-1.21)	<0.001				
**HCC Category 75**	Myasthenia Gravis/Myoneural Disorders, Inflammatory and Toxic Neuropathy	0.86 (0.83-0.88)	<0.001				
**HCC Category 79**	Seizure Disorders and Convulsions	0.88 (0.87-0.90)	<0.001	0.97 (0.96-0.98)	<0.001		
**HCC Category 80**	Coma, Brain Compression/Anoxic Damage			0.92 (0.90-0.93)	<0.001		

Variable	Description	Odds Ratio (Component 1)	p-value (Component 1)	Rate Ratio (Component 2)	p-value (Component 2)	Rate Ratio (Component 3)	p-value (Component 3)
**HCC Category 82**	Respirator Dependence/Tracheostomy Status	0.74 (0.71-0.76)	<0.001	0.97 (0.96-0.98)	<0.001		
**HCC Category 84**	Cardio-Respiratory Failure and Shock	1.29 (1.28-1.31)	<0.001			1.04 (1.04-1.05)	<0.001
**HCC Category 85**	Congestive Heart Failure	0.83 (0.82-0.84)	<0.001	0.98 (0.98-0.99)	<0.001	0.98 (0.98-0.99)	<0.001
**HCC Category 86**	Acute Myocardial Infarction	0.91 (0.89-0.92)	<0.001				
**HCC Category 87**	Unstable Angina and Other Acute Ischemic Heart Disease	0.81 (0.79-0.82)	<0.001				
**HCC Category 88**	Angina Pectoris	0.86 (0.85-0.87)	<0.001				
**HCC Category 96**	Specified Heart Arrhythmias	0.91 (0.91-0.92)	<0.001			1.00 (0.99-1.00)	0.01
**HCC Category 100**	Ischemic or Unspecified Stroke	0.84 (0.83-0.85)	<0.001				
**HCC Category 106**	Atherosclerosis of the Extremities with Ulceration or Gangrene	1.09 (1.06-1.11)	<0.001			1.07 (1.06-1.07)	<0.001
**HCC Category 107**	Vascular Disease with Complications	0.85 (0.84-0.86)	<0.001				
**HCC Category 111**	Chronic Obstructive Pulmonary Disease	1.18 (1.17-1.19)	<0.001				
**HCC Category 112**	Fibrosis of Lung and Other Chronic Lung Disorders	1.09 (1.07-1.11)	<0.001			1.02 (1.02-1.03)	<0.001
**HCC Category 114**	Aspiration and Specified Bacterial Pneumonias	1.75 (1.71-1.78)	<0.001	1.04 (1.04-1.05)	<0.001	1.08 (1.07-1.08)	<0.001
**HCC Category 115**	Pneumococcal Pneumonia, Empyema, Lung Abscess	1.81 (1.77-1.85)	<0.001	1.09 (1.08-1.10)	<0.001	1.06 (1.06-1.07)	<0.001
**HCC Category 134**	Dialysis Status			0.95 (0.94-0.96)	<0.001	0.98 (0.97-0.99)	<0.001
**HCC Category 135**	Acute Renal Failure	0.97 (0.96-0.98)	<0.001	0.99 (0.98-0.99)	<0.001		
**HCC Category 136**	Chronic Kidney Disease, Stage 5	0.74 (0.73-0.76)	<0.001	0.95 (0.94-0.96)	<0.001	0.98 (0.98-0.99)	<0.001
**HCC Category 137**	Chronic Kidney Disease, Severe (Stage 4)	0.86 (0.84-0.88)	<0.001	0.96 (0.96-0.97)	<0.001	0.97 (0.97-0.98)	<0.001
**HCC Category 138**	Chronic Kidney Disease, Moderate (Stage 3)	0.92 (0.91-0.93)	<0.001			0.99 (0.98-0.99)	<0.001
**HCC Category 157**	Pressure Ulcer of Skin with Necrosis Through to Muscle, Tendon, or Bone					1.04 (1.03-1.05)	<0.001
**HCC Category 158**	Pressure Ulcer of Skin with Full Thickness Skin Loss			0.97 (0.96-0.98)	<0.001		
**HCC Category 159**	Major Skin Burn or Condition			0.98 (0.97-0.99)	<0.001		
**HCC Category 161**	Chronic Ulcer of Skin, Except Pressure	1.40 (1.38-1.43)	<0.001	1.04 (1.04-1.05)	<0.001	1.04 (1.03-1.04)	<0.001
**HCC Category 169**	Vertebral Fractures without Spinal Cord Injury	0.83 (0.81-0.85)	<0.001				
**HCC Category 170**	Hip Fracture/Dislocation	0.86 (0.83-0.89)	<0.001	0.97 (0.95-0.98)	<0.001		

Variable	Description	Odds Ratio (Component 1)	p-value (Component 1)	Rate Ratio (Component 2)	p-value (Component 2)	Rate Ratio (Component 3)	p-value (Component 3)
**HCC Category 176**	Complications of Specified Implanted Device or Graft	1.59 (1.56-1.61)	<0.001	1.05 (1.05-1.06)	<0.001		
**HCC Category 186**	Major Organ Transplant or Replacement Status	1.60 (1.54-1.67)	<0.001	1.07 (1.06-1.09)	<0.001	1.07 (1.06-1.08)	<0.001
**HCC Category 188**	Artificial Openings for Feeding or Elimination					1.03 (1.02-1.03)	<0.001
**Admission to ICU**		1.58 (1.56-1.60)	<0.001	1.04 (1.03-1.05)	<0.001	1.08 (1.08-1.08)	<0.001
**Log(Days Present)**		1.63 (1.62-1.64)	<0.001				
**Admission to Surgical Specialties**		1.16 (1.15-1.17)	<0.001			0.97 (0.97-0.97)	<0.001

Blank cells indicate the covariate was not retained/included in that component model.

Abbreviations: AIDS: Acquired Immunodeficiency Syndrome; CCS: Clinical Classification Software; CNS: Central Nervous System; CT: Computed Tomography; DME: Durable Medical Equipment; GI: Gastrointestinal; HCC: Hierarchical Condition Categories; HIV: Human Immunodeficiency Virus; ICU: Intensive Care Unit; OR: Operating Room; 95%CI: 95% confidence interval
