# Supplementary material for: DASC-LOT Framework and S3 Metric: A Novel Evaluation and Benchmarking Method to Assess Initiation, Duration, and Spectrum of Antimicrobial Usage at Inpatient Hospital Settings
Source: Clin Infect Dis. 2026 Jun 25;83(3):e475–83. doi: 10.1093/cid/ciag389 (PMC13644869; doi:10.1093/cid/ciag389)
Supplement: Supplementary file 1 [file ciag389_supplementary_data.zip › Technical_Supplement 20260428.pdf]

Technical Supplement

DASC-LOT Framework and S^3 Metric: A Novel Evaluation and Benchmarking Method to Assess Initiation, Duration, and Spectrum of Antimicrobial Usage for Inpatient Hospital Settings

Clinical Infectious Diseases 2026

Michihiko Goto MD MS, Shungo Imai PhD Rph,
Hyunkeun Cho PhD, James A Merchant PhD, Daniel J Livorsi MD MS

April 28, 2026

Contents

1 List of Abbreviations: 2

2 Description of Dataset 3

3 Overview of Modeling Approach 4
3.1 Motivation and Structure of Three-Level Modeling . . . 4
3.2 Interpretations of Component-Specific Evaluations . . . 5
3.3 Composite Benchmarking Metric that Integrates Three Components (S^3 Metric) . . . 5
3.4 Approach to Variable Selection for Risk-Adjustments . . . 6

4 Model Specifications 6
4.1 Mathematical Notations . . . 6
4.2 Joint Probability Model Definition . . . 7
4.3 Component 1: Probability of Antibiotic Initiation . . . 7
4.4 Component 2: Number of Days on Antibiotics (LOT; Conditional Count Model) . . . 7
4.5 Component 3: Cumulative Spectrum Score (DASC; Conditional Count Model) . . . 8
4.6 Hospital-Specific and Marginal Predictions, and Calculation of S^3 Metric . . . 8

5 Implementation of DASC-LOT Framework in R 10
5.1 Preparation of Dataset and Screening for Variables . . . 10
5.2 Predictor Selection, Model Fit, and Parameter Estimates for Component 1 . . . 12
5.2.1 Variable Selection by LASSO . . . 12
5.2.2 Model Fit with the Developmental Dataset . . . 14
5.3 Predictor Selection, Model Fit, and Parameter Estimates for Component 2 . . . 14
5.3.1 Variable Selection by LASSO . . . 15
5.3.2 Model Fit with the Developmental Dataset . . . 16
5.4 Predictor Selection, Model Fit, and Parameter Estimates for Component 3 . . . 17
5.4.1 Variable Selection by LASSO . . . 17
5.4.2 Model Fit with the Developmental Dataset . . . 19
5.5 Application of Risk-Adjustment Model to 2024 Dataset and Estimation of Random Effects for Component 1 . . . 20
5.6 Application of Risk-Adjustment Model to 2024 Dataset and Estimation of Random Effects for Component 2 . . . 21
5.7 Application of Risk-Adjustment Model to 2024 Dataset and Estimation of Random Effects for Component 3 . . . 22
5.8 Calculation of S^3 Metric for Each Hospital . . . 24
5.9 Estimation of 95% Confidence Interval for S^3 Metric (Two-Stage Bootstrapping) . . . 24
5.10 Visualization of Benchmarking Results . . . 30
5.11 Visualization of Three-Axis Evaluation for Each Hospital . . . 31

Bibliography for Technical Supplement 35

1 List of Abbreviations:

- AUR: Antimicrobial Use and Resistance
- CCS: Clinical Classification Software
- CPT: Current Procedural Terminology
- DASC: Days of Antimicrobial Spectrum Coverage
- DOT: Days of Therapy
- DP: Days Present
- HCC: Hierarchical Condition Categories
- ICD: International Classification of Diseases
- ICU: Intensive Care Unit
- LASSO: Least Absolute Shrinkage and Selection Operator
- LOT: Length of Therapy
- NHSN: National Healthcare Safety Network
- VA: US Department of Veterans Affairs

2 Description of Dataset

The unit of measurement for datasets is individual unit stay for each patient. The structure of dataset and data type with descriptions are shown in Table 1. Two datasets for this project, `Inpatient_2022_2023` (the development dataset) and `Inpatient_2024` (the validation dataset), have identical structures.

**Variable**	**Level**	**Data Type**	**Description**
HospitalID	Facility	factor	Hospital Identifier
WardID	Unit	factor	Unit Identifier
PatientID	Patient	factor	Patient Identifier
Month	N/A	factor	Month of inpatient stay
DaysPresent	Patient	integer	Days Present calculated per NHSN AUR protocol
DASC	Patient	integer	Days of Antimicrobial Spectrum Coverage
LOT	Patient	integer	Length of Therapy
Abx	Patient	logical	Indicator for the presence or absence of antibiotic administration during the hospital stay; 1: any administration; 0: no administration
AGE_CAT	Patient	factor	Categorized age at the time of admission
Gender	Patient	factor	M: male; F: female
ICU	Patient	logical	Admission to Intensive Care Unit (ICU) service; 1 = ICU; 0 = non-ICU
SURGERY	Patient	logical	Admission to surgical services; 1: surgical service; 0: non-surgical service
HCC1-86	Patient	logical	A series of patient-level comorbidities classified by Hierarchical Condition Categories (HCC), based on ICD-10 diagnostic codes
CCS1-246	Patient	logical	A series of patient-level procedures performed during the inpatient stay classified by Clinical Classification Software (CCS), based on ICD-10 procedure codes and CPT codes

Table 1: Dataset Structure for the Analysis with Patient-Level Data

3 Overview of Modeling Approach

3.1 Motivation and Structure of Three-Level Modeling

Conceptually, antimicrobial stewardship activities in hospital settings include three major domains: 1) avoiding unnecessary antibiotic therapy; 2) avoiding antibiotic therapy with a longer duration than necessary; 3) avoiding antibiotic therapy with an excessive or unnecessary spectrum of coverage and promoting de-escalation. While there are other potential targets for antimicrobial stewardship activities (e.g., IV to PO switch, cost management), the majority of stewardship activities fall under these three domains.

The CDC NHSN protocol has used days of therapy (DOT) aggregated at the hospital/unit level as the basic metric, and SAAR is calculated with single-level negative binomial regression models. However, DOT only addresses the first two domains of antimicrobial stewardship activities. In addition, because DOT counts concurrent therapeutic agents separately, it unfairly penalizes appropriate combination therapies. We addressed these issues by developing days of antimicrobial spectrum coverage (DASC)[1], incorporating spectrum into the evaluation while avoiding unfairly penalizing appropriate combination therapies. DASC is the cumulative number of daily antimicrobial spectrum coverage (ASC) scores. Therefore, it can be viewed as a composite evaluation of stewardship activities to address the duration and spectrum of therapy.

We previously described an approach to model DASC with zero-inflated negative binomial regressions to perform patient-level risk adjustment and benchmarking of hospitals.[2] While it improved content validity from the single-level negative binomial model using DOT aggregated at the hospital/unit level, there are remaining problems:

- Although the risk-adjusted DASC incorporates all three components of stewardship activities, the conditional count component of the zero-inflated negative binomial model assumes that the number of days and the spectrum scores have equal meaning. For example, a patient receives DASC = 80 with an antibiotics with ASC = 8 for 10 days or another antibiotics with ASC = 4 for 20 days. In real clinical practice, this assumption does not hold.
- The interpretation of hospital-specific random intercepts in the conditional count component of zero-inflated negative binomial regression can confuse frontline antimicrobial stewards, as larger deviations from 0 may be due to therapy duration, spectrum of therapy, or both (second and third domains).
- It would be ideal if we could inform each hospital about their performance on three domains separately.

To address these limitations in directly modeling DOT or DASC, we propose a three-level conditional modeling framework that disaggregates variabilities in antimicrobial usage at inpatient hospital settings by combining DASC and LOT ("DASC-LOT Framework"). We define a three-component model with separate hospital-specific random intercepts for all components:

- Binary component (component 1): Antibiotic initiation ($Y_{1ij} = 1$ for receiving antibiotics; $Y_{1ij} = 0$ for not receiving antibiotics).
  - This component reflects <u>the probability of antibiotics initiation</u> ($\text{P}(Y_{1ij} = 1)$) during the hospital stay.
  - Since patients are more likely to receive antibiotics when they stay longer, this component includes Days Present with logarithmic transformation as a regular predictor (logarithmic transformation was used because there is a likely non-linear relationship between likelihood of antibiotic initiation and days present).
  - Clinically, this reflects the stewardship programs' activities to avoid unnecessary antibiotic initiation/empiric therapy.
  - We model this component using hierarchical logistic regression with hospital-specific random intercept (ω_j).
- Conditional count component (component 2): Number of days on antibiotics (Y_{2ij}; Length of Therapy [LOT]) given antibiotic use ($Y_{2ij} \geq 1$ when $Y_{1ij} = 1$, and $Y_{2ij} = 0$ when $Y_{1ij} = 0$ by definition).
  - This component reflects <u>the number of days patients received antibiotics (when started) during the hospital stay, divided by days present (antibiotic density)</u>.
  - Note: this is not the same as "Days of Therapy (DOT)" in CDC-NHSN protocol, as we do not double-count combination therapy. For example, a patient who received vancomycin and ceftriaxone for 7 days will receive 7 for LOT while receiving 14 for DOT.
  - The denominator is days present. Therefore, this includes $\log(DaysPresent)$ as an offset variable.
  - Clinically, this reflects the stewardship program activity to avoid unnecessarily long antibiotic therapy.

- We model this component using hierarchical zero-truncated negative binomial regression model (conditional on $Y_{1ij} = 1$), with hospital-specific random intercept (ν_j).

- Conditional count component (component 3): Cumulative spectrum score (Y_{3ij}; Days of Antimicrobial Spectrum Coverage [DASC]) given number of days on antibiotics (LOT). $Y_{3ij} \geq 1$ when $Y_{2ij} \geq 1$ (and $Y_{1ij} = 1$) and $Y_{3ij} = 0$ when $Y_{2ij} = 0$ (and $Y_{1ij} = 0$) by definition.
  - This component reflects the cumulative number of ASC during the hospital stay (DASC) when started, divided by LOT (average daily antibiotic spectrum coverage [ASC] score).
  - The denominator is LOT. Therefore, we include $\log(Y_{2ij})$ as an offset variable.
  - Clinically, this reflects the stewardship program activity to avoid excessively broad-spectrum therapy and de-escalation.
  - We model this component by hierarchical zero-truncated negative binomial regression (conditional on $Y_{2ij} \geq 1$), with hospital-specific random intercept (τ_j).

3.2 Interpretations of Component-Specific Evaluations

Each hospital j has a random intercept for each component (ω_j, ν_j, τ_j), allowing hospital-specific variation. These three random intercepts reflect each hospital's performance in three different components of the stewardship program activities, ω_j for antimicrobial initiation, ν_j for antimicrobial therapy duration, and τ_j for antimicrobial spectrum. Interpretations of exponentiated random intercept values are:

- $exp(\omega_j)$: Odds ratio indicating how likely j-th hospital starts antibiotics compared to a hypothetical "standardized" hospital with $\omega = 0$
- $exp(\nu_j)$: Rate ratio indicating how much longer or shorter j-th hospital uses antibiotics compared to a hypothetical "standardized" hospital with $\nu = 0$, with given days present (i.e., rate ratio for antibiotic density)
- $exp(\tau_j)$: Rate ratio indicating how much broader or narrower agents j-th hospital uses, compared to a hypothetical "standardized" hospital with $\tau = 0$, with given length of therapy (i.e., rate ratio for daily average ASC score)

3.3 Composite Benchmarking Metric that Integrates Three Components (S^3 Metric)

To translate the three-component DASC–LOT framework into a single, clinician-friendly benchmarking measure, we define the S^3 metric as a hospital-level *predicted-to-expected* (P/E) ratio for total antibiotic spectrum burden (DASC). For a given inpatient stay, the model separately characterizes three clinically intuitive quantities: (i) how often antibiotics are initiated, (ii) how long therapy continues once started (LOT), and (iii) how broad the selected regimen is on average (daily spectrum intensity, reflected by DASC per day of therapy). Combining these three components yields a risk-adjusted estimate of the *total spectrum burden* attributable to that stay. When aggregated across all stays in a hospital, this provides a hospital-level summary of "how much DASC we would anticipate" for that hospital.

Predicted Total DASC (Hospital-Specific; Conditional on Hospital Effects). The *predicted* total DASC for hospital j is obtained by *combining outputs from all three model components*—antibiotic initiation, length of therapy (LOT) given initiation, and average daily spectrum intensity given LOT—while evaluating each component at hospital j's estimated random intercept. Although the final predicted quantity is expressed in units of DASC, it is therefore conditional on the full three-step prescribing process: whether antibiotics are started, how many days of therapy accrue, and how broad the selected regimen is on those days. Clinically, this can be interpreted as the expected antimicrobial usage (total DASC) *if the same hospital saw the same set of patients again*, under its typical practice patterns for initiation frequency, duration of therapy, and antibiotic selection/spectrum. In other words, the predicted value reflects both the hospital's case mix and its hospital-specific stewardship-related tendencies across *all three* components.

Expected Total DASC (Standardized/Population-Based). The *expected* total DASC is computed for the *same set of patients* under a standardized hospital that represents average practice after risk adjustment. Mathematically, this corresponds to a standardized prediction obtained by setting hospital-specific random intercepts to 0 (the reference hospital), so differences are driven by patient-level risk factors rather than hospital practice variation. Clinically, the expected value answers: "How much DASC would we expect for these patients *if* they were treated at an average (standardized) hospital?"

Hospital-Level S^3 Metric Definition. For hospital j, the S^3 metric is:

$$S^3Metric_j = \frac{\text{Predicted Total DASC for Hospital } j}{\text{Expected Total DASC for Hospital } j \text{ Under a Standardized Hospital}}$$

As described above, "Predicted Total DASC" is a hospital-specific prediction that incorporates random intercepts from all three components (ω_j, ν_j, τ_j). "Expected Total DASC" is a marginal prediction calculated by setting all random intercept values to 0. S^3 Metric > 1 indicates higher DASC than expected after risk adjustment (greater overall antimicrobial usage burden), whereas S^3 Metric < 1 indicates lower DASC than expected (smaller overall antimicrobial usage burden).

3.4 Approach to Variable Selection for Risk-Adjustments

Similar to our previous publication,[2] we randomly split the dataset into ten random subsets and performed cross-validation in each subset to determine regularization parameter (lambda) and selected variables, for each component independently. Our approach differs from traditional single-pass approaches. Our developmental dataset (data from 2022-2023) contains approximately 2 million observations and 300 patient-level candidate variables. Applying LASSO with cross-validation directly to the full dataset is computationally intensive and presents practical limitations due to its very large memory space requirements (not just to us but to any researchers who may want to replicate our work). Therefore, we adapted a strategy similar in spirit to stability selection[3]: we performed LASSO (with internal cross-validation to determine the most appropriate lambda value) independently across 10 random subsets (each 200,000 observations), and selected only variables that were chosen in at least 8 subsets.

Our approach was motivated by four reasons:

1. Reducing computational burden: Each subset is small enough to allow efficient LASSO with cross-validation using standard computing infrastructure. We used a workstation equipped with 12 CPU cores and 64 GB of memory, which allowed us to run LASSO models in parallel. We attempted to perform LASSO and traditional backward elimination with the entire training dataset first but could not fit into our computational environment.

2. Improving variable stability and avoiding overfitting: Rather than relying on a single model fit, our approach emphasizes repeatedly selected variables across multiple random subsets. This guards against the inclusion of variables whose selection is driven by random noise in any one sample and mimics the benefits of stability selection. Notably, variables that did not appear in the majority of subsets were often weakly predictive and exhibited low coefficients when re-evaluated on the full dataset.

3. Preserving the benefits of LASSO while enhancing generalizability: After selecting the stable set of variables (those selected in at least 8 subsets), we refit a final model on the full dataset using only these variables. This refitting step allowed us to recover much of the statistical efficiency and predictive accuracy that could be lost by operating only on subsets while maintaining an interpretable and robust variable set.

4. The lack of available software packages to perform LASSO for a fully joint multivariate model that combines a binary outcome and two conditional zero-truncated count outcomes.

We recognize that our strategy might reduce LASSO's full-model shrinkage behavior. However, because variables needed to pass a stricter inclusion threshold (selected in 8/10 subsets), this approach acts as a conservative filter, minimizing the risk of false positives in variable selection and increasing stability and applicability. We also recognize that our conservative approach could potentially exclude important variables that were not selected in most iterations. However, given that each random subset has approximately 200,000 observations, we believe the likelihood was minimal.

4 Model Specifications

4.1 Mathematical Notations

Let:

- i be index for patients.
- j be index for hospitals.
- $Y_{1ij} \in \{0, 1\}$: $Y_{1ij} = 1$ if patient i in hospital j received antibiotics, 0 otherwise, modeled by component 1 (logistic regression).

- $Y_{2ij} \in \{1, 2, \ldots\}$ be the number of days on antibiotics (length of therapy: LOT) for patient i in hospital j, conditional on $Y_{1ij} = 1$, modeled by component 2 (zero-truncated negative binomial regression).
- $Y_{3ij} \in \{1, 2, \ldots\}$ be the cumulative spectrum score (DASC), conditional on $Y_{2ij} \geq 1$, modeled by component 3 (zero-truncated negative binomial regression).
- D_{ij} be the number of days patient i stayed in j-th hospitals (days present).
- X_{ij} be a vector of patient-level predictor values included in Component 1 for patient i in hospital j.
- X'_{ij} be a vector of patient-level predictor values included in Component 2 for patient i in hospital j.
- X''_{ij} be a vector of patient-level predictor values included in Component 3 for patient i in hospital j.
- β be a set of coefficients for patient-level predictors included in Component 1.
- γ be a set of coefficients for patient-level predictors included in Component 2.
- δ be a set of coefficients for patient-level predictors included in Component 3.
- ω_j, ν_j, τ_j be hospital-specific random intercepts for Components 1, 2, and 3, respectively.

4.2 Joint Probability Model Definition

We define a joint probability model for the cumulative antibiotic spectrum score (DASC) for the i-th patient in the j-th hospital.

$$p(Y_{3ij}, Y_{2ij}, Y_{1ij} | X_{ij}, X'_{ij}, X''_{ij}) = p(Y_{1ij}|X_{ij}) \times p(Y_{2ij}|Y_{1ij} = 1, X'_{ij}) \times p(Y_{3ij}|Y_{2ij} \geq 1, X''_{ij})$$

By *joint*, we mean that we specify a single likelihood for the DASC-generating process by decomposing it into three conditional components: (i) antibiotic initiation, (ii) duration of therapy (LOT) conditional on initiation, and (iii) spectrum intensity (DASC per LOT) conditional on therapy duration. These components are linked through conditioning and hospital-specific random intercepts, allowing us to obtain coherent patient- and hospital-level predictions of total DASC and to derive standardized metric for benchmarking (e.g., S^3 Metric). The model consists of three components:

4.3 Component 1: Probability of Antibiotic Initiation

Let Y_{1ij} be a binary variable indicating whether patient i in hospital j received antibiotics ($Y_{1ij} = 1$ if yes, 0 otherwise). In this component, we include days present (DP) as a regular predictor with a logarithmic transformation. The probability of initiation is modeled using logistic regression:

$$\text{logit}\left(P(Y_{1ij} = 1)\right) = X_{ij}\beta + \omega_j$$

where:

- $\text{logit}(p) = \log\left(\frac{p}{1-p}\right)$,
- X_{ij} = a vector for patient-level predictors for the i-th patient in the j-th hospital,
- β = a vector of fixed-effect coefficients for patient-level predictors,
- $\omega_j \sim \mathcal{N}(0, \sigma^2_\omega)$ is the hospital-specific random intercept.

4.4 Component 2: Number of Days on Antibiotics (LOT; Conditional Count Model)

Given that a patient received antibiotics ($Y_{1ij} = 1$), let Y_{2ij} be a positive integer indicating the number of days patient i in hospital j received antibiotics. By including days present (DP) as an offset variable with a logarithmic transformation, this model essentially estimates the antibiotic density (LOT per DP) if a patient receives any antibiotics during a hospital stay. We assume LOT (Y_{2ij}) follows a zero-truncated negative binomial distribution (because $Y_{2ij} \geq 1$ when $Y_{1ij} = 1$ by definition):

$$[Y_{2ij}|Y_{1ij} = 1] \sim \text{ZTNB}(\mu_{Y_{2ij}}, \theta_{Y_2}), Y_{2ij} = 1, 2, 3, ...$$

with the log-link function:

$$\log(E[Y_{2ij} \mid Y_{1ij} = 1]) = X'_{ij}\gamma + \log(D_{ij}) + \nu_j$$

where:

- $\log(D_{ij})$ is an offset to model the number of days on antibiotics per days present,
- X'_{ij} = a vector for patient-level predictors for the i-th patient in the j-th hospital,
- γ = a vector of fixed-effect coefficients for patient-level predictors,
- $\nu_j \sim \mathcal{N}(0, \sigma^2_\nu)$ is the hospital-specific random intercept,
- θ_{Y_2} is the dispersion parameter of the zero-truncated negative binomial distribution.

4.5 Component 3: Cumulative Spectrum Score (DASC; Conditional Count Model)

Given that a patient received antibiotics for Y_{2ij} days ($Y_{2ij} \geq 1$), let Y_{3ij} be a positive integer indicating the cumulative spectrum score (DASC). By including Y_{2ij} as an offset variable with a logarithmic transformation, this model essentially estimates the daily average spectrum score (DASC per LOT) while receiving antibiotics if a patient receives any antibiotics during a hospital stay. We assume DASC (Y_{3ij}) follows a zero-truncated negative binomial distribution (because $Y_{3ij} \geq 1$ when $Y_{2ij} \geq 1$ by definition):

$$[Y_{3ij}|Y_{2ij} \geq 1] \sim \text{ZTNB}(\mu_{Y_{3ij}}, \theta_{Y_3}), Y_{3ij} = 1, 2, 3, ...$$

with the log-link function:

$$\log(E[Y_{3ij} \mid Y_{2ij} \geq 1]) = X''_{ij}\delta + \log(Y_{2ij}) + \tau_j$$

where:

- $\log(Y_{2ij})$ is an offset to model the cumulative spectrum score per day of therapy (i.e., the daily average spectrum score),
- X''_{ij} = a vector for patient-level predictors for the i-th patient in the j-th hospital,
- δ = a vector of fixed-effect coefficients for patient-level predictors,
- $\tau_j \sim \mathcal{N}(0, \sigma^2_\tau)$ is the hospital-specific random intercept,
- θ_{Y_3} is the dispersion parameter for the zero-truncated negative binomial distribution.

When computing the predicted DASC functional, we approximate the integration over LOT by substituting $E[Y_{2ij}|Y_1ij = 1]$. Our goal is the model-implied expected total DASC, integrating over the LOT-generating process, rather than the conditional expectation of DASC given the observed LOT (i.e., we consider the final DASC prediction as a plug-in estimator). This also allows us to avoid the undefined denominator ($log(0)$) issue when $LOT = 0$ (i.e., LOT is unobserved for counterfactual predictions). Therefore, $E[Y_{3ij} \mid Y_{2ij} \geq 1]$ can be expressed as:

$$\begin{aligned}\log(E[Y_{3ij} \mid Y_{2ij} \geq 1]) &= X''_{ij}\delta + \log(E[Y_{2ij} \mid Y_{1ij} = 1]) + \tau_j \\ &= X''_{ij}\delta + X'_{ij}\gamma + \log(D_{ij}) + \nu_j + \tau_j\end{aligned}$$

4.6 Hospital-Specific and Marginal Predictions, and Calculation of S^3 Metric

This subsection provides explicit mathematical definitions of (i) hospital-specific ("predicted") LOT and DASC that incorporate the hospital random effects from all three components, (ii) marginal/standardized ("expected") LOT and DASC that remove hospital effects to enable fair comparisons across hospitals, and (iii) hospital-level S^3 metrics.

Component-Level Hospital-Specific Predictions. Using the fitted three-component model, define the hospital-specific (conditional) predictions:

$$\hat{p}_{ij}^{(j)} \;=\; \Pr(Y_{1ij}=1 \mid X_{ij}, \hat{\beta}, \hat{\omega}_j) \;=\; \text{logit}^{-1}(X_{ij}\hat{\beta}+\hat{\omega}_j),$$

$$\widehat{\mu}_{2ij}^{(j)} \;=\; E(Y_{2ij} \mid Y_{1ij}=1, X'_{ij}, D_{ij}, \hat{\gamma}, \hat{\nu}_j) \;=\; \exp\bigl(X'_{ij}\hat{\gamma}+\log(D_{ij})+\hat{\nu}_j\bigr),$$

$$\widehat{\mu}_{3ij}^{(j)} \;=\; E(Y_{3ij} \mid Y_{2ij}\geq 1, X''_{ij}, \widehat{\mu}_{2ij}^{(j)}, \hat{\delta}, \hat{\tau}_j) \;=\; \exp\bigl(X''_{ij}\hat{\delta}+\log(\widehat{\mu}_{2ij}^{(j)})+\hat{\tau}_j\bigr).$$

Substituting $\log(\widehat{\mu}_{2ij}^{(j)}) = X'_{ij}\hat{\gamma}+\log(D_{ij})+\hat{\nu}_j$ yields:

$$\widehat{\mu}_{3ij}^{(j)} \;=\; \exp\bigl(X''_{ij}\hat{\delta}+X'_{ij}\hat{\gamma}+\log(D_{ij})+\hat{\nu}_j+\hat{\tau}_j\bigr),$$

highlighting that the DASC component depends on both the LOT component and the Component-3 random effect.

Hospital-Specific ("Predicted") Total LOT and DASC for a Patient. The hospital-specific predicted *total* LOT and DASC for patient i in hospital j (marginal over the unobserved initiation indicator) are:

$$\widehat{\text{LOT}}_{ij}^{(j)} \;=\; E(Y_{2ij} \mid X_{ij}, X'_{ij}, D_{ij}, \hat{\beta}, \hat{\gamma}, \hat{\omega}_j, \hat{\nu}_j) \;=\; \hat{p}_{ij}^{(j)} \times \widehat{\mu}_{2ij}^{(j)},$$

$$\widehat{\text{DASC}}_{ij}^{(j)} \;=\; E(Y_{3ij} \mid X_{ij}, X'_{ij}, X''_{ij}, D_{ij}, \hat{\beta}, \hat{\gamma}, \hat{\delta}, \hat{\omega}_j, \hat{\nu}_j, \hat{\tau}_j) \;=\; \hat{p}_{ij}^{(j)} \times \widehat{\mu}_{3ij}^{(j)}.$$

Thus, although the predicted quantity is DASC, $\widehat{\text{DASC}}_{ij}^{(j)}$ incorporates information from *all three components* and their hospital random effects: initiation ($\hat{\omega}_j$), duration ($\hat{\nu}_j$), and spectrum intensity ($\hat{\tau}_j$).

Marginal/Standardized ("Expected") Predictions for a Patient. To quantify expected antimicrobial use for a hospital *given its case mix but without hospital-specific practice patterns*, we use marginal (standardized) predictions by setting hospital random effects to the reference level (0). These are sometimes called *marginal predictions* because they remove the conditional dependence on hospital j.

Define:

$$\hat{p}_{ij}^{(0)} = \text{logit}^{-1}(X_{ij}\hat{\beta}), \qquad \widehat{\mu}_{2ij}^{(0)} = \exp\bigl(X'_{ij}\hat{\gamma}+\log(D_{ij})\bigr), \qquad \widehat{\mu}_{3ij}^{(0)} = \exp\bigl(X''_{ij}\hat{\delta}+X'_{ij}\hat{\gamma}+\log(D_{ij})\bigr),$$

and the corresponding standardized totals:

$$\widehat{\text{LOT}}_{ij}^{(0)} = \hat{p}_{ij}^{(0)} \times \widehat{\mu}_{2ij}^{(0)}, \qquad \widehat{\text{DASC}}_{ij}^{(0)} = \hat{p}_{ij}^{(0)} \times \widehat{\mu}_{3ij}^{(0)}.$$

Hospital-Level Aggregation. Let $\mathcal{I}_j$ index patients/hospitalizations in hospital j. We aggregate to obtain hospital-level predicted and expected totals:

$$\widehat{\text{LOT}}_{j}^{(j)} = \sum_{i\in\mathcal{I}_j} \widehat{\text{LOT}}_{ij}^{(j)}, \qquad \widehat{\text{LOT}}_{j}^{(0)} = \sum_{i\in\mathcal{I}_j} \widehat{\text{LOT}}_{ij}^{(0)},$$

$$\widehat{\text{DASC}}_{j}^{(j)} = \sum_{i\in\mathcal{I}_j} \widehat{\text{DASC}}_{ij}^{(j)}, \qquad \widehat{\text{DASC}}_{j}^{(0)} = \sum_{i\in\mathcal{I}_j} \widehat{\text{DASC}}_{ij}^{(0)}.$$

The "predicted" totals incorporate hospital-specific practice patterns via $\hat{\omega}_j, \hat{\nu}_j, \hat{\tau}_j$, whereas the "expected" totals reflect the same hospital's patient mix under the reference hospital effect.

S^3 Metric. The S^3 metric is defined as a predicted-to-expected (P/E) ratio for total DASC. For hospital j, we compute:

$$S_j^3 = \frac{\widehat{\text{DASC}}_{j}^{(j)}}{\widehat{\text{DASC}}_{j}^{(0)}}.$$

Values greater than 1 indicate higher-than-expected DASC given the case mix (i.e., higher overall antimicrobial usage burden after accounting for initiation, duration, and agent choice), whereas values less than 1 indicate lower-than-expected DASC (i.e., lower overall antimicrobial usage burden).

5 Implementation of DASC-LOT Framework in R

This section describes the end-to-end implementation steps for DASC-LOT framework in R. We used following versions of software and key packages:

- R: 4.5.0
- dplyr: 1.1.4
- caret: 7.0-1
- glmmTMB: 1.1.11
- glmnet: 4.1-8
- ggplot2: 3.5.2

5.1 Preparation of Dataset and Screening for Variables

This subsection describes the data-preparation steps used before model fitting. The goal is to build a stable and interpretable candidate predictor set by (i) removing extremely rare binary indicators, (ii) screening high-dimensional HCC/CCS categories for univariate association with antimicrobial use, and (iii) checking for strong collinearity among remaining indicators (defined as correlation coefficient > 0.7).

To ensure consistent variable definitions across time, any predictors removed during screening in the developmental dataset (2022–2023) are removed from the application dataset (2024) using the same variable list. The prevalence threshold and p-value cutoffs are pragmatic defaults and can be tightened or relaxed depending on sample size and the desired bias–variance trade-off. First, we excluded logical variables (HCC and CCS categories) with very low prevalence (<0.5%), because those are unlikely to be able to provide reliable estimates for fixed-effect coefficients.

```r
# Loading libraries
library(dplyr)

# Select columns that start with "HCC_" or "CCS_" and create a table with frequencies
selected_columns <- Inpatient_2022_2023 %>%
    select(starts_with("HCC_"), starts_with("CCS_"))
Variable_Count <- data.frame(
    Variable = character(),
    TrueCount = integer()
    )

for (i in 1:dim(selected_columns)[2]){
    Variable = names(selected_columns)[i]
    TrueCount = sum(as.integer(selected_columns[,i]))
    loopCount <- cbind(Variable, TrueCount)
    Variable_Count <- rbind(Variable_Count, loopCount)
    }

Variable_Count$TrueCount <- as.integer(Variable_Count$TrueCount)
Variable_Count$TotalCount <- nrow(selected_columns)
Variable_Count <- Variable_Count %>%
    mutate(TruePercentage = (TrueCount/TotalCount)*100)

# Identify variables with less than 0.5% prevalence
InfrequentVariable <- Variable_Count %>%
    filter(TruePercentage <0.5) %>%
    select(Variable) %>%  # Select relevant columns
    pull()

# Remove infrequent variables
Inpatient_2022_2023 <- Inpatient_2022_2023 %>%
    select(-any_of(InfrequentVariable))  # Select relevant columns
Inpatient_2024 <- Inpatient_2024 %>%
    select(-any_of(InfrequentVariable))  # Select relevant columns
```

Next, we screen variables by univariate analysis with Mann-Whitney U test (aka., Wilcoxon rank sum test) to assess associationsiations between candidate variables (HCC and CCS categories) and antimicrobial usage.

```
# Loading libraries
library(dplyr)
library(parallel)
library(doParallel)
library(foreach)

# Select columns that start with "HCC_" or "CCS_" and create a table with frequencies
selected_columns <- Inpatient_2022_2023 %>%
    select(starts_with("HCC_"), starts_with("CCS_"))
DASCperDP <- Inpatient_2022_2023$DASC/Inpatient_2022_2023$DaysPresent

# Initialize cluster for parallel processing
num_cores <- detectCores() - 2 #Leaving 2 cores for general operations
cl <- makeCluster(num_cores)  # Define 'cl'
registerDoParallel(cl)  # Register parallel backend

# Perform parallel computation
DASCUnivariateResult <- foreach(i = 1:ncol(selected_columns),
                               .combine = rbind,
                               .packages = c("dplyr")) %dopar% {
        Variable <- selected_columns[, i]
        wilcox_test_result <- wilcox.test(DASCperDP ~ Variable)
        VariableName <- names(selected_columns)[i]
        p_value <- wilcox_test_result$p.value
        c(VariableName, p_value)
        }

# Convert result to data frame and set column names
DASCUnivariateResult <- as.data.frame(DASCUnivariateResult, stringsAsFactors = FALSE)
colnames(DASCUnivariateResult) <- c("Variable", "p_value")

# Stop the cluster
stopCluster(cl)  # Stop the parallel cluster

DASCUnivariateResult$p_value <- as.numeric(DASCUnivariateResult$p_value)
DASCUnrelatedColumns <- DASCUnivariateResult %>%
    filter(p_value >= 0.05)
DASCVariablesToBeRemoved <- DASCUnrelatedColumns$Variable

Inpatient_2022_2023 <- Inpatient_2022_2023 %>%
    select(-all_of(DASCVariablesToBeRemoved))  # Select relevant columns
Inpatient_2024 <- Inpatient_2024 %>%
    select(-all_of(DASCVariablesToBeRemoved))  # Select relevant columns
```

Lastly, we checked collinearities among HCC and CCS categories, and identify combinations with strong collinearities (defined by correlation coefficient >0.7).

```
# Loading libraries
library(dplyr)
library(caret)

# Check collinearity among HCC and CCS variables
selected_columns <- Inpatient_2022_2023 %>%
    select(starts_with("HCC_"), starts_with("CCS_"))  # Select relevant columns

# Check for multicollinearity and inspect r >0.7
cor_matrix <- cor(selected_columns, use = "pairwise.complete.obs")
highly_correlated <- findCorrelation(cor_matrix, cutoff = 0.7)
highly_correlated_vars <- rownames(cor_matrix)[highly_correlated]
print(highly_correlated_vars)  # Print object to console/output
```

```r
# Three sets of collinearities were identified
# Set 1: "HCC_18" "HCC_19"
# Set 2: "HCC_134" "HCC_136" "CCS_58"
# Set 3: "CCS_15" "CCS_241"

# Find which variables are highly correlated with HCC_18
highly_correlated_with_HCC_18 <- cor_matrix["HCC_18",] > 0.7
print(names(highly_correlated_with_HCC_18[highly_correlated_with_HCC_18]))
# "HCC_18" "HCC_19"

# Find which variables are highly correlated with HCC_134
highly_correlated_with_HCC_134 <- cor_matrix["HCC_134",] > 0.7
print(names(highly_correlated_with_HCC_134[highly_correlated_with_HCC_134]))
# "HCC_134" "HCC_136" "CCS_58"

# Find which variables are highly correlated with CCS_Cat15
highly_correlated_with_CCS_15 <- cor_matrix["CCS_15",] > 0.7
print(names(highly_correlated_with_CCS_15[highly_correlated_with_CCS_15]))
# "CCS_15" "CCS_241"

# HCC_134 (dialysis status), HCC_136 (Chronic Kidney Disease, Stage 5),
# and CCS_58 (hemodialysis) are correlated as expected. Remove HCC_134
# and CCS_58, as HCC_136 had a stronger association with antimicrobial use.

# HCC_18 (diabetes with chronic complications) and HCC_19 (diabetes
# without complication) are correlated. Remove HCC_19, as HCC_18 had
# a stronger association with antimicrobial use.

# CCS_15 (Lens and cataract procedures) and CCS_241 (Visual aids and
# other optical supplies) are correlated. Remove CCS_241, as CCS_15
# had a stronger association with antimicrobial use.

Inpatient_2022_2023 <- Inpatient_2022_2023 %>%
    select(-c(HCC_134, CCS_58, HCC_19, CCS_241))
Inpatient_2024 <- Inpatient_2024 %>%
    select(-c(HCC_134, CCS_58, HCC_19, CCS_241))
```

5.2 Predictor Selection, Model Fit, and Parameter Estimates for Component 1

Component 1 models the probability of antibiotic initiation (binary outcome). This subsection first performs high-dimensional predictor screening using LASSO logistic regression on repeated random subsets with cross-validation, and then carries forward robustly selected predictors into a multivariable hierarchical logistic regression model.

The hierarchical model is fit with a hospital-specific random intercept to quantify between-hospital variation after risk adjustment. The code below is written to be reproducible (fixed random seed) and can be adapted by changing the number of subsets, the cross-validation folds, or the selection threshold (e.g., requiring selection in ≥ 8 of 10 subsets).

5.2.1 Variable Selection by LASSO

As detailed in Section 3.4, we ran LASSO in ten random subsets and performed cross-validation in each subset to determine the lambda value, then selected variables (i.e., keeping predictors with coefficients > 0) separately. We then selected variables that were chosen in at least 8 subsets.

```r
library(glmnet)
library(Matrix)
library(doParallel)
library(foreach)
library(stringr)

# (Component 1 uses all admissions; no restriction to LOT > 0 is applied here.)
```

```r
# List of predictor candidates
predictor_candidates <- Inpatient_2022_2023 %>%
    select(-c("PatientID", "HospitalID", "WardID", "DASC", "LOT", "Abx")) %>%
    names()
factor_columns <- c("Gender", "Month", "AGE_CAT")

# Set seed number for reproducibility
set.seed(12345)  # Set random seed for reproducibility

# Prepare data for glmnet package
y <- as.integer(Inpatient_2022_2023$Abx)
X <- Inpatient_2022_2023 %>%
    select(all_of(predictor_candidates))

# Convert categorical variables to dummy variables
X_model_matrix <- model.matrix(~ ., data = X)[, -1]  # remove intercept

# Combine X and y
full_data <- data.frame(Y = y, X_model_matrix)

# Split into 10 random subsets
n_subsets <- 10  # Define `n_subsets`
subset_indices <- sample(rep(1:n_subsets, length.out = nrow(full_data)))

# Register parallel backend
n_cores <- parallel::detectCores() - 2  # leave two cores free
cl <- makeCluster(n_cores)
registerDoParallel(cl)  # Register parallel backend

# Run LASSO in parallel on each subset
selected_vars_list <- foreach(i = 1:n_subsets,
                              .packages = c("glmnet", "dplyr", "Matrix")) %dopar% {
    subset_data <- full_data[subset_indices == i, ]  # Define `subset_data`
    y_subset <- subset_data$Y  # Define `y_subset`
    X_subset <- subset_data %>% select(-Y)  # Select relevant columns

    # Convert to matrix
    X_mat <- as.matrix(X_subset)  # Define `X_mat`

    # LASSO with CV
    cvfit <- cv.glmnet(X_mat, y_subset, family = "binomial", alpha = 1, nfolds = 5)

    # Extract selected variables
    coef_i <- coef(cvfit, s = "lambda.1se")  # Extract model coefficients
    selected_vars <- rownames(coef_i)[coef_i[,1] != 0]
    setdiff(selected_vars, "(Intercept)")
    }

# Stop parallel backend
stopCluster(cl)  # Stop the parallel cluster
registerDoSEQ()

# Tabulate frequency of each variable across subsets
all_selected <- unlist(selected_vars_list)
var_freq <- table(all_selected)

# List variables selected in at least 8 out of 10 subsets
selected_ge8 <- names(var_freq[var_freq >= 8])
selected_ge8 <- as.data.frame(selected_ge8)
colnames(selected_ge8) <- "Variable"

selected_ge8 <- selected_ge8 %>%
    mutate(Variable = str_replace_all(Variable, "TRUE", "")) %>%
```

```
    mutate(Variable = str_replace_all(Variable, "GenderM", "Gender")) %>%
    mutate(Variable = ifelse(str_detect(Variable, "AGE_CAT"), "AGE_CAT", Variable)) %>%
    mutate(Variable = ifelse(str_detect(Variable, "Month"), "Month", Variable)) %>%
    distinct(Variable) %>%
    pull()
```

5.2.2 Model Fit with the Developmental Dataset

```
library(glmmTMB)
library(parallel)

# Set up the control parameters for the glmmTMB optimizer (adjust as needed)
num_cores <- detectCores() -2
ctrl <- glmmTMBControl(
  optimizer = optim,
  optArgs = list(method = "L-BFGS-B", maxfun = 100000),
  eigval_check = FALSE,
  parallel = num_cores
)

FinalFormulaSingle_Comp1 <- as.formula(paste0("Abx ~ ",
                                        paste0(selected_ge8, collapse = (" + "))))

FinalFormula_Comp1 <- as.formula(paste0("Abx ~ ",
                                        paste0(selected_ge8, collapse = (" + ")),
                                        " + (1 | HospitalID)"))

Inpatient_2022_2023 <- Inpatient_2022_2023[order(Inpatient_2022_2023$HospitalID),]

# Fit the multivariable logistic regression model without hospital-specific
# random intercepts to obtain start values for the hierarchical model
SingleModelMVComp1 <- glmmTMB(FinalFormulaSingle_Comp1,
                             data = Inpatient_2022_2023,
                             family = binomial(),
                             control = ctrl)
start_vals <- list(beta = fixef(SingleModelMVComp1)$cond)  # Extract fixed effects
rm(SingleModelMVComp1)

# Fit the multivariable hierarchical logistic regression model
FinalModelMVComp1 <- glmmTMB(FinalFormula_Comp1,  # Fit hierarchical model with glmmTMB
                           data = Inpatient_2022_2023,
                           family = binomial(),
                           start = start_vals,
                           control = ctrl)
summary(FinalModelMVComp1)
```

5.3 Predictor Selection, Model Fit, and Parameter Estimates for Component 2

Component 2 models duration of therapy among admissions with antibiotics, using length of therapy (LOT) as a strictly positive count outcome. Because penalized zero-truncated negative binomial (ZTNB) regression is not routinely available in standard software, we use penalized overdispersed Poisson (quasi-Poisson) regression with an offset for log(days present) as a computationally efficient screening step.

After screening, the selected predictors are refit in a ZTNB model using
texttt glmmTMB with the same offset and a hospital-specific random intercept. This two-step strategy prioritizes scalable variable reduction while ensuring final inference is based on a distribution that accommodates overdispersion and truncation at zero.

5.3.1 Variable Selection by LASSO

We used a similar approach as Component 1, and performed variable selections by LASSO in 10 random subsets, then selected variables that were chosen in at least 8 subsets. However, because penalized estimation procedures for zero-truncated negative binomial regression are not available in standard software, we implemented a two-step approach. First, we used LASSO regression under a Poisson likelihood[4, 5] (`glmnet` package) to screen candidate predictors as a computationally efficient screening step (final refitting uses a negative binomial model to accommodate overdispersion). This step leverages the same mean–variance structure as the zero-truncated model but benefits from efficient penalized estimation. We then refitted the selected predictors in a zero-truncated negative binomial model[6, 7] (`glmmTMB` package) to ensure unbiased effect estimates under the appropriate distributional assumption.

```
# As component 2 is a conditional model (LOT >0), filter dataset by the condition
Inpatient_LOT_2022_2023 <- Inpatient_2022_2023 %>%
    filter(LOT >0)

library(MASS)
library(glmnet)
library(parallel)
library(doParallel)
library(dplyr)
library(stringr)

set.seed(12345)  # Set random seed for reproducibility

randomnumber <- sample(0:9, nrow(Inpatient_LOT_2022_2023), replace = TRUE)
Inpatient_LOT_2022_2023 <- cbind(Inpatient_LOT_2022_2023, randomnumber)

selection_output <- list()

n_cores <- detectCores() - 2
cl <- makeCluster(n_cores)
registerDoParallel(cl)

Sys.setenv(OMP_NUM_THREADS = "1",
           MKL_NUM_THREADS = "1",
           OPENBLAS_NUM_THREADS = "1")

for (i in 0:9){
  df <- Inpatient_LOT_2022_2023 %>%
    dplyr::filter(randomnumber == i) %>%
    dplyr::select(-randomnumber)

  logical_vars <- sapply(df, is.logical)
  df[logical_vars] <- lapply(df[logical_vars], function(x) as.numeric(x))

  integer_vars <- sapply(df, is.integer)
  df[integer_vars] <- lapply(df[integer_vars], function(x) as.numeric(x))


  factor_vars <- names(Filter(is.factor, df))

  non_factor_df <- df[, !(names(df) %in% factor_vars), drop = FALSE]
  factor_formula <- as.formula(paste("~ ", paste(factor_vars, collapse = " + ")))
  factor_dummies <- model.matrix(factor_formula, data = df)
  processed_df <- cbind(non_factor_df, factor_dummies)
  processed_df <- as.data.frame(processed_df)
  processed_df <- processed_df %>%
    dplyr::select(-Intercept)

  gc()

  # Basic hygiene
  stopifnot(is.numeric(processed_df$LOT), is.numeric(processed_df$Log_DaysPresent))
```

```
  processed_df <- subset(processed_df, is.finite(LOT) & is.finite(Log_DaysPresent))

  # Build X without response/offset and drop zero-variance columns
  X <- processed_df[ , setdiff(names(processed_df), c("LOT","Log_DaysPresent"))]
  nzv <- vapply(X, function(z) sd(z, na.rm=TRUE) > 0, logical(1))
  X <- as.matrix(X[ , nzv, drop=FALSE])

  y <- processed_df$LOT
  off <- processed_df$Log_DaysPresent

  # Standardize X for glmnet
  X_std <- scale(X, center = TRUE, scale = TRUE)

  # Fit cross-validated LASSO model
  cv_pois <- cv.glmnet(x = X_std, y = y, family = quasipoisson(), offset = off,
                       nfolds = 5, parallel = TRUE)

  keep <- which(as.numeric(coef(cv_pois, s = "lambda.1se"))[-1] != 0)
  X_small <- X[ , keep, drop = FALSE]

  selected_predictors <- colnames(X_small) %>%
    lapply(function(x) str_replace_all(x, "AGE_CAT.*", "AGE_CAT")) %>%
    lapply(function(x) str_replace_all(x, "Month.*", "Month")) %>%
    lapply(function(x) str_replace_all(x, "Gender.*", "Gender")) %>%
    unique() %>%
    unlist()

  selection_output <- c(selection_output, selected_predictors) %>%
    unlist()

  gc()
}

stopCluster(cl)  # Stop the parallel cluster

frequency_table <- table(selection_output)
frequency_table <- as.data.frame(frequency_table)

selected_ge8 <- frequency_table %>%
  dplyr::filter(Freq >=8) %>%
  dplyr::select(selection_output) %>%
  mutate(selected = as.character(selection_output)) %>%
  pull() %>%
  as.list() %>%
  unlist()
```

5.3.2 Model Fit with the Developmental Dataset

```
ztnb_formula <- as.formula(paste0("LOT ~ ", paste0(selected_ge8, collapse = " + "),
                                  " + offset(Log_DaysPresent)"))
ztnb_formula_MTL <- as.formula(paste0("LOT ~ ", paste0(selected_ge8, collapse = " + "),
                                      " + offset(Log_DaysPresent) + (1|HospitalID)"))

library(glmmTMB)
library(parallel)

# Set up the control parameters for the glmmTMB optimizer (adjust as needed)
num_cores <- detectCores() -2
ctrl <- glmmTMBControl(
  optimizer = optim,
  optArgs = list(method = "L-BFGS-B", maxfun = 100000),
```

```
  eigval_check = FALSE,
  parallel = num_cores
)

# Fit a non-hierarchical model with glmmTMB to obtain start values
SingleModelMVComp2 <- glmmTMB(ztnb_formula,
                             data = Inpatient_LOT_2022_2023,
                             family=truncated_nbinom2,
                             control = ctrl)
start_beta <- fixef(SingleModelMVComp2)$cond  # Extract fixed effects
start_betadisp <- fixef(SingleModelMVComp2)$disp  # Extract fixed effects

# Fit hierarchical model with glmmTMB
FinalModelMVComp2 <- glmmTMB(ztnb_formula_MTL,
                          data = Inpatient_LOT_2022_2023,
                          family=truncated_nbinom2,
                          start = list(
                            beta = start_beta,
                            betadisp = start_betadisp
                          ),
                          control = ctrl)
summary(FinalModelMVComp2)
```

5.4 Predictor Selection, Model Fit, and Parameter Estimates for Component 3

Component 3 models cumulative spectrum burden (DASC) conditional on antibiotic initiation and LOT ≥ 1. An offset for log(LOT) is used so that model coefficients can be interpreted on the scale of average daily spectrum burden (approximately DASC per LOT day).

As in Component 2, we use penalized overdispersed Poisson regression as a screening step, followed by refitting the selected predictors in a ZTNB model with `glmmTMB`. This subsection provides code for both steps and yields a final hierarchical model that can be used for hospital-level benchmarking. Component 3 models DASC conditional on antibiotic initiation and LOT ≥ 1, with an offset for log(LOT) to estimate the average daily spectrum burden (DASC per LOT).

5.4.1 Variable Selection by LASSO

We again used a similar approach as Components 1 and 2, and performed variable selections by LASSO in 10 random subsets, then selected variables that were chosen in at least 8 subsets. We used the `glmnet` package with a quasi-Poisson family (`family = quasipoisson()`) for covariate screening, which allows for overdispersion while preserving the same mean model as Poisson regression. Because this LASSO step is used for variable screening, the final effect estimates are obtained by refitting the full model under the intended distributional assumption (zero-truncated negative binomial via `glmmTMB`) on the full dataset restricted to LOT ≥ 1 (see Section 6.3.1).

```
library(MASS)
library(glmnet)
library(parallel)
library(doParallel)
library(dplyr)
library(stringr)

set.seed(12345)  # Set random seed for reproducibility

randomnumber <- sample(0:9, nrow(Inpatient_LOT_2022_2023), replace = TRUE)
Inpatient_LOT_2022_2023 <- cbind(Inpatient_LOT_2022_2023, randomnumber)

selection_output <- list()

n_cores <- detectCores() - 2
cl <- makeCluster(n_cores)
registerDoParallel(cl)
```

```r
Sys.setenv(OMP_NUM_THREADS = "1",
           MKL_NUM_THREADS = "1",
           OPENBLAS_NUM_THREADS = "1")

for (i in 0:9){
  df <- Inpatient_LOT_2022_2023 %>%
    dplyr::filter(randomnumber == i) %>%
    dplyr::select(-randomnumber) %>%
    dplyr::filter(LOT >0)

  logical_vars <- sapply(df, is.logical)
  df[logical_vars] <- lapply(df[logical_vars], function(x) as.numeric(x))

  integer_vars <- sapply(df, is.integer)
  df[integer_vars] <- lapply(df[integer_vars], function(x) as.numeric(x))


  factor_vars <- names(Filter(is.factor, df))

  non_factor_df <- df[, !(names(df) %in% factor_vars), drop = FALSE]
  factor_formula <- as.formula(paste("~ ", paste(factor_vars, collapse = " + ")))
  factor_dummies <- model.matrix(factor_formula, data = df)
  processed_df <- cbind(non_factor_df, factor_dummies)
  processed_df <- as.data.frame(processed_df)
  processed_df <- processed_df %>%
    dplyr::select(-`(Intercept)`)

  gc()

  # Basic hygiene
  stopifnot(is.numeric(processed_df$DASC), is.numeric(processed_df$Log_LOT))
  processed_df <- subset(processed_df, is.finite(DASC) & is.finite(Log_LOT))

  # Build X without response/offset and drop zero-variance columns
  X <- processed_df[ , setdiff(names(processed_df), c("DASC","Log_LOT"))]
  nzv <- vapply(X, function(z) sd(z, na.rm=TRUE) > 0, logical(1))
  X <- as.matrix(X[ , nzv, drop=FALSE])

  y <- processed_df$DASC  # Define `y`
  off <- processed_df$Log_LOT  # Define `off`

  # Standardize X for glmnet
  X_std <- scale(X, center = TRUE, scale = TRUE)

  # Fit cross-validated LASSO model
  cv_pois <- cv.glmnet(x = X_std, y = y, family = quasipoisson(), offset = off,
                       nfolds = 5, parallel = TRUE)

  keep <- which(as.numeric(coef(cv_pois, s = "lambda.1se"))[-1] != 0)  # Extract model
      coefficients
  X_small <- X[ , keep, drop = FALSE]

  selected_predictors <- colnames(X_small) %>%
    lapply(function(x) str_replace_all(x, "AGE_CAT.*", "AGE_CAT")) %>%
    lapply(function(x) str_replace_all(x, "Month.*", "Month")) %>%
    lapply(function(x) str_replace_all(x, "Gender.*", "Gender")) %>%
    unique() %>%
    unlist()

  selection_output <- c(selection_output, selected_predictors) %>%
    unlist()

  gc()
```

```
}

stopCluster(cl)  # Stop the parallel cluster

frequency_table <- table(selection_output)
frequency_table <- as.data.frame(frequency_table)

selected_ge8 <- frequency_table %>%
  dplyr::filter(Freq >=8) %>%
  dplyr::select(selection_output) %>%
  mutate(selected = as.character(selection_output)) %>%
  pull() %>%
  as.list() %>%
  unlist()
```

5.4.2 Model Fit with the Developmental Dataset

Using the final set of selected predictors, we fit a hierarchical zero-truncated negative binomial regression model with hospital-specific random intercepts and an offset for `log(LOT)`.

```
library(glmmTMB)
library(parallel)

ztnb_formula <- as.formula(paste0("DASC ~ ", paste0(selected_ge8, collapse = " + "),
                                  " + offset(Log_LOT)"))
ztnb_formula_MTL <- as.formula(paste0("DASC ~ ", paste0(selected_ge8, collapse = " + "),
                                      " + offset(Log_LOT) + (1|HospitalID)"))

# Set up the control parameters for the glmmTMB optimizer
num_cores <- detectCores() -2
ctrl <- glmmTMBControl(
  optimizer = optim,
  optArgs = list(method = "L-BFGS-B", maxfun = 100000),
  eigval_check = FALSE,
  parallel = num_cores
)

# Fit a non-hierarchical model with glmmTMB
SingleModelMVComp3 <- glmmTMB(ztnb_formula,
                              data = Inpatient_LOT_2022_2023,
                              family=truncated_nbinom2,
                              control = ctrl)

start_beta <- fixef(SingleModelMVComp3)$cond  # Extract fixed effects
start_betadisp <- fixef(SingleModelMVComp3)$disp  # Extract fixed effects

# Fit hierarchical model with glmmTMB
FinalModelMVComp3 <- glmmTMB(ztnb_formula_MTL,
                             data = Inpatient_LOT_2022_2023,
                             family=truncated_nbinom2,
                             start = list(
                               beta = start_beta,
                               betadisp = start_betadisp
                             ),
                             control = ctrl)
summary(FinalModelMVComp3)
```

5.5 Application of Risk-Adjustment Model to 2024 Dataset and Estimation of Random Effects for Component 1

This subsection applies the finalized Component 1 risk-adjustment model to the 2024 evaluation dataset. We first compute the fixed-effect linear predictor from the developmental model for each admission and then treat it as an emphoffset in a new model fit to 2024 data.

Fitting only an intercept and a hospital-specific random intercept on top of the fixed-effect offset isolates hospital-level deviations from expected initiation probability under the same case-mix. The resulting "conditional" predictions include hospital random effects (predicted), whereas the "unconditional" predictions set random effects to zero (expected).

```
AbxUsageData <- Inpatient_2024 %>%
  select(PatientSID, PatientICN, HospitalID, Abx, LOT, DASC, DaysPresent)

HospitalIDRanefSummary <- Inpatient_2024 %>%
  select(HospitalID) %>%
  unique()

# Set up the control parameters for the glmmTMB optimizer
num_cores <- detectCores() -2
ctrl <- glmmTMBControl(
  optimizer = optim,
  optArgs = list(method = "L-BFGS-B", maxfun = 100000),
  eigval_check = FALSE,
  parallel = num_cores
)

# Extract the list of predictors
VarsComp1 <- names(fixef(FinalModelMVComp1)$cond) %>%
  str_remove("TRUE") %>%
  str_subset("(Intercept)", negate = TRUE)

# Extract the fixed effect coefficients
FixefComp1 <- fixef(FinalModelMVComp1)$cond

# Create a dataset with only predictors in component 1
DfComp1 <- Inpatient_2024 %>%
  select(all_of(VarsComp1))

# Convert a dataset with only predictors to matrix
Intercept <- rep(1, nrow(DfComp1))
DfComp1 <- as.matrix(cbind(Intercept, DfComp1))

# Calculate linear predictor without random effects and expected probability for
    component 1
LinearPredictorComp1 <- DfComp1 %*% FixefComp1

AbxUsageData <- cbind(AbxUsageData, LinearPredictorComp1)

# Fit a model with 2024 data with an offset predictor
Model2024Comp1 <- glmmTMB(Abx ~ 1 + offset(LinearPredictorComp1) + (1|HospitalID),
                          data = AbxUsageData,
                          family = binomial(),
                          control = ctrl)

# Generate model-based predictions specific to each hospital
LinearPredictorComp1_cond <- predict(Model2024Comp1,
                                     type = "link")
# Generate marginal predictions
LinearPredictorComp1_uncond <- predict(Model2024Comp1,
                                       newdata = AbxUsageData,
                                       re.form = ~0,
```

```
                                        type = "link")
PredictProbComp1 <- plogis(LinearPredictorComp1_cond)
ExpectProbComp1 <- plogis(LinearPredictorComp1_uncond)
AbxUsageData <- cbind(AbxUsageData, LinearPredictorComp1_cond,
    LinearPredictorComp1_uncond,
                      PredictProbComp1, ExpectProbComp1)

# Extract HospitalID-specific random intercepts
HospitalIDRanefComp1 <- ranef(Model2024Comp1, condVar = TRUE)$cond$HospitalID
HospitalIDRanefVarComp1 <- attr(HospitalIDRanefComp1, "condVar")

HospitalIDRanefComp1 <- as.data.frame(HospitalIDRanefComp1)
HospitalIDRanefComp1$HospitalID <- rownames(HospitalIDRanefComp1)
rownames(HospitalIDRanefComp1) <- NULL
colnames(HospitalIDRanefComp1) <- c("InterceptComp1", "HospitalID")
HospitalIDRanefComp1$VarianceComp1 <- HospitalIDRanefVarComp1[1,1,]
HospitalIDRanefComp1$StdErrComp1 <- sqrt(HospitalIDRanefComp1$VarianceComp1)
attr(HospitalIDRanefComp1, "condVar") <- NULL

HospitalIDRanefSummary <- HospitalIDRanefSummary %>%
  left_join(HospitalIDRanefComp1, by = "HospitalID")
```

5.6 Application of Risk-Adjustment Model to 2024 Dataset and Estimation of Random Effects for Component 2

Analogous to Component 1, this subsection applies the finalized Component 2 (LOT) model to the 2024 dataset. The fixed-effect linear predictor from the developmental ZTNB model is computed for each admission and used as an offset when refitting a random-intercept-only model in 2024.

This approach yields hospital-specific random intercepts for LOT after risk adjustment, as well as admission-level predicted and expected LOT values. These quantities are later aggregated to the hospital level and combined with Components 1 and 3 to derive predicted and expected DASC.

```
# Extract the list of predictors from model
VarsComp2 <- names(fixef(FinalModelMVComp2)$cond) %>%
  str_remove("TRUE") %>%
  str_subset("(Intercept)", negate = TRUE)

# Extract fixed-effect coefficients
FixefComp2 <- fixef(FinalModelMVComp2)$cond

# Create a dataframe with only predictors in component 2 model
DfComp2 <- Inpatient_2024 %>%
  select(all_of(VarsComp2))

# Offset variable (Log_DaysPresent)
OffsetComp2 <- Inpatient_2024$Log_DaysPresent

# Calculate linear predictor for component 2
Intercept <- rep(1, nrow(DfComp2))
DfComp2 <- as.matrix(cbind(Intercept, DfComp2))

LinearPredictorComp2 <- DfComp2 %*% FixefComp2
LinearPredictorComp2 <- LinearPredictorComp2 + OffsetComp2

# Add to AbxUsageData dataframe
AbxUsageData <- cbind(AbxUsageData, LinearPredictorComp2)

# Fit model in 2024 data to estimate HospitalID-specific random intercepts
Model2024Comp2 <- glmmTMB(LOT ~ 1 + offset(LinearPredictorComp2) + (1|HospitalID),
                          data = AbxUsageData[AbxUsageData$LOT >0,],
                          family = truncated_nbinom2,
```

```
                          control = ctrl)

# Generate model-based predictions specific to each hospital
LinearPredictorComp2_cond <- predict(Model2024Comp2,
                                      type = "link",
                                      newdata = AbxUsageData)
# Generate marginal predictions
LinearPredictorComp2_uncond <- predict(Model2024Comp2,
                                        type = "link",
                                        re.form = ~0,
                                        newdata = AbxUsageData)
PredictLOTComp2 <- exp(LinearPredictorComp2_cond)
ExpectLOTComp2 <- exp(LinearPredictorComp2_uncond)
AbxUsageData <- cbind(AbxUsageData, LinearPredictorComp2_cond,
    LinearPredictorComp2_uncond,
                      PredictLOTComp2, ExpectLOTComp2)

# Extract and summarize HospitalID-specific random intercepts
HospitalIDRanefComp2 <- ranef(Model2024Comp2, condVar = TRUE)$cond$HospitalID
HospitalIDRanefVarComp2 <- attr(HospitalIDRanefComp2, "condVar")

HospitalIDRanefComp2 <- as.data.frame(HospitalIDRanefComp2)
HospitalIDRanefComp2$HospitalID <- rownames(HospitalIDRanefComp2)
rownames(HospitalIDRanefComp2) <- NULL
colnames(HospitalIDRanefComp2) <- c("InterceptComp2", "HospitalID")
HospitalIDRanefComp2$VarianceComp2 <- HospitalIDRanefVarComp2[1,1,]
HospitalIDRanefComp2$StdErrComp2 <- sqrt(HospitalIDRanefComp2$VarianceComp2)
attr(HospitalIDRanefComp2, "condVar") <- NULL

HospitalIDRanefSummary <- HospitalIDRanefSummary %>%
  left_join(HospitalIDRanefComp2, by = "HospitalID")
```

5.7 Application of Risk-Adjustment Model to 2024 Dataset and Estimation of Random Effects for Component 3

This subsection applies the finalized Component 3 (DASC with offset for log(LOT)) model to 2024. As in the prior components, we compute the developmental-model fixed-effect predictor and use it as an offset when estimating a hospital-specific random intercept in the 2024 data.

The output includes both conditional and unconditional predictions for DASC (on the appropriate scale given the offset), along with hospital-level random effect estimates. These are used to compute hospital-level predicted and expected spectrum burden in the composite benchmarking metric.

```
# Extract predictors for component 3
VarsComp3 <- names(fixef(FinalModelMVComp3)$cond) %>%
  str_remove("TRUE") %>%
  str_subset("(Intercept)", negate = TRUE)

# Extract fixed effect coefficients
FixefComp3 <- fixef(FinalModelMVComp3)$cond

# Create a dataframe only with predictors in component 3
DfComp3 <- Inpatient_2024 %>%
  mutate(AGE_CATAGE_45_54 = ifelse(AGE_CAT == "AGE_45_54", 1, 0),
         AGE_CATAGE_55_64 = ifelse(AGE_CAT == "AGE_55_64", 1, 0),
         AGE_CATAGE_65_74 = ifelse(AGE_CAT == "AGE_65_74", 1, 0),
         AGE_CATAGE_75_84 = ifelse(AGE_CAT == "AGE_75_84", 1, 0),
         AGE_CATAGE_GE85 = ifelse(AGE_CAT == "AGE_GE85", 1, 0))

DfComp3 <- DfComp3 %>%
  select(all_of(VarsComp3))
```

```
# Calculate linear predictor for component 3
# linear predictor for component 2 will be offset variable because the denominator is
    expected LOT
Intercept <- rep(1, nrow(DfComp3))
MatrixComp3 <- as.matrix(cbind(Intercept, DfComp3))

LinearPredictorComp3 <- as.numeric(MatrixComp3 %*% FixefComp3)

AbxUsageData <- cbind(AbxUsageData, LinearPredictorComp3)

AbxUsageData <- AbxUsageData %>%
  mutate(OffsetComp3 = log(PredictLOTComp2))

# Fit model with 2024 data for component 3 to estimate HospitalID-specific random
    intercepts
Model2024Comp3 <- glmmTMB(DASC ~ 1 + offset(LinearPredictorComp3) + offset(OffsetComp3)
    + (1|HospitalID),
                           data = AbxUsageData[AbxUsageData$LOT >0,],
                           family = truncated_nbinom2,
                           control = ctrl)

# Generate model-based predictions specific to each hospital
LinearPredictorComp3_cond <- predict(Model2024Comp3,
                                      type = "link",
                                      newdata = AbxUsageData)

AbxUsageData <- AbxUsageData %>%
  mutate(OffsetComp3 = log(ExpectLOTComp2))
# Generate marginal predictions
LinearPredictorComp3_uncond <- predict(Model2024Comp3,
                                        type = "link",
                                        re.form = ~0,
                                        newdata = AbxUsageData)

PredictDASCComp3 <- exp(LinearPredictorComp3_cond)
ExpectDASCComp3 <- exp(LinearPredictorComp3_uncond)
AbxUsageData <- cbind(AbxUsageData, LinearPredictorComp3_cond,
    LinearPredictorComp3_uncond,
                      PredictDASCComp3, ExpectDASCComp3)

# Calculate conditional predictions for LOT and DASC
AbxUsageData <- AbxUsageData %>%
  mutate(PredictLOTComp2 = PredictProbComp1 * PredictLOTComp2,
         ExpectLOTComp2 = ExpectProbComp1 * ExpectLOTComp2,
         PredictDASCComp3 = PredictProbComp1 * PredictDASCComp3,
         ExpectDASCComp3 = ExpectProbComp1 * ExpectDASCComp3)

AbxUsageData$OffsetComp3 <- NULL

HospitalIDRanefComp3 <- ranef(Model2024Comp3_cond, condVar = TRUE)$cond$HospitalID  #
    Extract random effects
HospitalIDRanefVarComp3 <- attr(HospitalIDRanefComp3, "condVar")

HospitalIDRanefComp3 <- as.data.frame(HospitalIDRanefComp3)
HospitalIDRanefComp3$HospitalID <- rownames(HospitalIDRanefComp3)
rownames(HospitalIDRanefComp3) <- NULL
colnames(HospitalIDRanefComp3) <- c("InterceptComp3", "HospitalID")
HospitalIDRanefComp3$VarianceComp3 <- HospitalIDRanefVarComp3[1,1,]
HospitalIDRanefComp3$StdErrComp3 <- sqrt(HospitalIDRanefComp3$VarianceComp3)
attr(HospitalIDRanefComp3, "condVar") <- NULL

HospitalIDRanefSummary <- HospitalIDRanefSummary %>%
  left_join(HospitalIDRanefComp3, by = "HospitalID")
```

5.8 Calculation of S^3 Metric for Each Hospital

This subsection aggregates admission-level quantities to the hospital level and computes the composite benchmarking metric, S^3 metric, defined as the predicted-to-expected (P/E) ratio of DASC. Predicted DASC incorporates all three model components and their hospital-specific random effects, whereas expected DASC corresponds to the marginal prediction with random effects set to zero.

The code below joins component-specific predictions, summarizes observed/predicted/expected totals by hospital, and calculates point estimates of S^3 metric for reporting and visualization.

```
HospitalID_PE_PointEstimateSummary <- AbxUsageData %>%
  group_by(HospitalID) %>%
  summarise(ExpectDASCComp3 = sum(ExpectDASCComp3),
            PredictDASCComp3 = sum(PredictDASCComp3)) %>%
  ungroup() %>%
  mutate(S3Metric = PredictDASCComp3/ExpectDASCComp3)
```

5.9 Estimation of 95% Confidence Interval for S^3 Metric (Two-Stage Bootstrapping)

To quantify uncertainty in the hospital-level S^3 metric estimates, we use a two-stage bootstrap that resamples patients (or admissions) within hospitals and resamples hospitals across the system. This preserves the hierarchical structure of the data while reflecting both within-hospital sampling variability and between-hospital variation.[8, 9] We also used bootstrap resampling to quantify uncertainty in hospital random effects because their sampling distributions can be non-normal and model-based standard errors may be unreliable, especially with small or unbalanced hospital sample sizes.

For each bootstrap replicate, component models are refit (or their random effects are re-estimated) and the hospital-level S^3 metric is recalculated. The empirical bootstrap distribution is then used to compute percentile-based 95% confidence intervals. Bootstrap confidence intervals for S^3 Metric capture uncertainty due to the mix of patients and admissions observed in the 2024 evaluation cohort. For these intervals, we treated the risk-adjustment relationships learned from the 2022–2023 developmental data as fixed (implemented as offsets) and did not re-estimate those patient-level effects within each bootstrap replicate. In other words, the intervals assume that patient-level predictors have the same effects in 2024 as in 2022–2023.

```
# Set up the control parameters for the glmmTMB optimizer
num_cores <- detectCores() -2
ctrl <- glmmTMBControl(
  optimizer = optim,
  optArgs = list(method = "L-BFGS-B", maxfun = 100000),
  eigval_check = FALSE,
  parallel = num_cores
)

VarsComp1 <- names(fixef(FinalModelMVComp1)$cond) %>%  # Extract fixed effects
  str_remove("TRUE") %>%
  str_subset("(Intercept)", negate = TRUE)
FixefComp1 <- fixef(FinalModelMVComp1)$cond

VarsComp2 <- names(fixef(FinalModelMVComp2)$cond) %>%
  str_remove("TRUE") %>%
  str_subset("(Intercept)", negate = TRUE)
FixefComp2 <- fixef(FinalModelMVComp2)$cond

VarsComp3 <- names(fixef(FinalModelMVComp3)$cond) %>%
  str_remove("TRUE") %>%
  str_subset("(Intercept)", negate = TRUE)
FixefComp3 <- fixef(FinalModelMVComp3)$cond

start_vals_comp1 <- list(beta = fixef(Model2024Comp1)$cond)
```

```
start_vals_comp2 <- list(beta = fixef(Model2024Comp2)$cond,
                         theta = fixef(Model2024Comp2)$disp)
start_vals_comp3 <- list(beta = fixef(Model2024Comp3)$cond,
                         theta = fixef(Model2024Comp3)$disp)

# One-time prep (sort + index)
prep_nested_boot <- function(df, cluster_col = "HospitalID", patient_col = "PatientID")
    {
  dt <- as.data.table(df)

  # Use setorderv() with character column names
  setorderv(dt, c(cluster_col, patient_col))
  dt[, .row := .I]

  # Stable run-length group id for (hospital, patient)
  dt[, hp_gid := rleidv(.SD, cols = c(cluster_col, patient_col))]

  # Row indices per (hospital, patient) group
  idx_by_gid <- split(dt$.row, dt$hp_gid)

  # Map: hospital -> list of hp_gids (groups) and patients present
  # Use character 'by' and .() for list columns, avoid .. pronoun here
  map_h <- dt[, .(  # Define `map_h`
    gids = .(unique(hp_gid)),
    pats = .(unique(get(patient_col)))
  ), by = c(cluster_col)]

  list(dt = dt,
       idx_by_gid = idx_by_gid,
       map_h = map_h,
       cluster_col = cluster_col,
       patient_col = patient_col)
}

# Fast nested bootstrap using the precomputed structure
nested_boot_fast <- function(prep, keep_tags = FALSE, seed = NULL) {
  dt        <- prep$dt
  map_h     <- prep$map_h
  idx_list  <- prep$idx_by_gid
  cluster_col <- prep$cluster_col

  H <- nrow(map_h)
  if (H == 0L) return(as.data.frame(dt[0]))

  # Sample hospitals by row index of map_h
  h_draw <- sample.int(H, H, replace = TRUE)

  chunks <- vector("list", H)
  jj <- 1L  # Define `jj`

  for (h_i in h_draw) {
    gids <- map_h$gids[[h_i]]   # vector of hp_gid for this hospital
    P    <- length(gids)
    if (P == 0L) next

    p_draw <- sample.int(P, P, replace = TRUE)
    chosen_gids <- gids[p_draw]

    # Collect row indices; each hp_gid maps to a contiguous slice
    chunks[[jj]] <- unlist(idx_list[chosen_gids], use.names = FALSE)
    jj <- jj + 1L  # Define `jj`
  }
```

```r
  idx <- unlist(chunks[seq_len(jj - 1L)], use.names = FALSE)
  out <- dt[idx]

  if (!keep_tags) {
    out[, c("hp_gid", ".row") := NULL]
  } else {
    # lightweight tag; keep hp_gid so you can trace which (hospital,patient) block a
        row came from
    out[, .boot_seq := seq_len(.N)]
  }

  as.data.frame(out)
}

# Run data preparation function
prep_nested_boot(Inpatient_2024)

iteration <- 0

# Create data frame `HospitalID_PESummary_Boot`
HospitalID_PESummary_Boot <- data.frame(
  HospitalID = factor(),
  PredictProbComp1 = numeric(),
  ExpectProbComp1 = numeric(),
  PredictLOTComp2 = numeric(),
  ExpectLOTComp2 = numeric(),
  PredictDASCComp3 = numeric(),
  ExpectDASCComp3 = numeric(),
  LOT_PERatio = numeric(),
  S3Metric = numeric(),
  BootstrapSample = integer()
)

HospitalID_Ranef_Boot <- data.frame(
  HospitalID = factor(),
  RanefComp1 = numeric(),
  RanefComp2 = numeric(),
  RanefComp3 = numeric(),
  BootstrapSample = integer()
)

seed.number <- 12345 # Set seed number for reproducibility

for (b in (iteration +1):1000) {
  seed.number.loop <- seed.number + b
  set.seed(seed.number.loop)
  # Draw a nested bootstrap sample
  df_b <- nested_boot_fast(Inpatient_2024)

  AbxUsageData <- df_b %>%
    select(PatientSID, HospitalID, Abx, LOT, DASC, DaysPresent)

  #### Component 1 ####
  # Create a dataset with only predictors in component 1
  DfComp1 <- df_b %>%
    select(all_of(VarsComp1))

  # Convert a dataset with only predictors to matrix
  Intercept <- rep(1, nrow(DfComp1))  # Define `Intercept`
  DfComp1 <- as.matrix(cbind(Intercept, DfComp1))  # Define `DfComp1`

  # Calculate linear predictor without random effects and expected probability for
      component 1
```

```
LinearPredictorComp1 <- DfComp1 %*% FixefComp1  # Define `LinearPredictorComp1`

AbxUsageData <- cbind(AbxUsageData, LinearPredictorComp1)  # Define `AbxUsageData`

# Fit a model with 2024 data with offset predictor
Model2024Comp1 <- glmmTMB(Abx ~ 1 + offset(LinearPredictorComp1) + (1|HospitalID),  #
    Fit hierarchical model with glmmTMB
                          data = AbxUsageData,
                          family = binomial(),
                          start = start_vals_comp1,
                          control = ctrl)
LinearPredictorComp1_cond <- predict(Model2024Comp1,
                                     type = "link")
LinearPredictorComp1_uncond <- predict(Model2024Comp1,
                                       newdata = AbxUsageData,
                                       re.form = ~0,
                                       type = "link")
PredictProbComp1 <- plogis(LinearPredictorComp1_cond)  # Define `PredictProbComp1`
ExpectProbComp1 <- plogis(LinearPredictorComp1_uncond)  # Define `ExpectProbComp1`

RanefComp1 <- as.data.frame(ranef(Model2024Comp1)$cond$HospitalID)
RanefComp1 <- RanefComp1 %>%
  rownames_to_column()
colnames(RanefComp1) <- c("HospitalID", "RanefComp1")
attr(RanefComp1, "condvar") <- NULL

AbxUsageData <- cbind(AbxUsageData, LinearPredictorComp1_cond,
    LinearPredictorComp1_uncond,
                      PredictProbComp1, ExpectProbComp1)

rm(list=ls()[! ls() %in% c("FixefComp1", "FixefComp2", "FixefComp3",
                           "VarsComp1", "VarsComp2", "VarsComp3",
                           "Inpatient_2024", "nested_boot_fast",
                              "HospitalID_PESummary_Boot",
                           "HospitalID_Ranef_Boot", "RanefComp1",
                           "num_cores", "ctrl", "df_b", "AbxUsageData",
                              "seed.number", "b",
                           "start_vals_comp1","start_vals_comp2",
                              "start_vals_comp3")])
gc()

#### Component 2 ####
# Create a dataframe with only predictors in component 2 model
DfComp2 <- df_b %>%
  select(all_of(VarsComp2))

# Offset variable (Log_DaysPresent)
OffsetComp2 <- df_b$Log_DaysPresent

# Calculate linear predictor for component 2
Intercept <- rep(1, nrow(DfComp2))
DfComp2 <- as.matrix(cbind(Intercept, DfComp2))

LinearPredictorComp2 <- DfComp2 %*% FixefComp2
LinearPredictorComp2 <- LinearPredictorComp2 + OffsetComp2

# Add to AbxUsageData dataframe
AbxUsageData <- cbind(AbxUsageData, LinearPredictorComp2)

# Fit model in 2024 data to estimate HospitalID-specific random intercepts
Model2024Comp2 <- glmmTMB(LOT ~ 1 + offset(LinearPredictorComp2) + (1|HospitalID),
                          data = AbxUsageData[AbxUsageData$LOT >0,],
                          family = truncated_nbinom2,
```

```
                             control = ctrl)

# Generate model-based predictions specific to each hospital
LinearPredictorComp2_cond <- predict(Model2024Comp2,
                                     type = "link",
                                     newdata = AbxUsageData)
# Generate marginal predictions
LinearPredictorComp2_uncond <- predict(Model2024Comp2,
                                       type = "link",
                                       re.form = ~0,
                                       newdata = AbxUsageData)
PredictLOTComp2 <- exp(LinearPredictorComp2_cond)
ExpectLOTComp2 <- exp(LinearPredictorComp2_uncond)

RanefComp2 <- as.data.frame(ranef(Model2024Comp2)$cond$HospitalID)
RanefComp2 <- RanefComp2 %>%
  rownames_to_column()
colnames(RanefComp2) <- c("HospitalID", "RanefComp2")
attr(RanefComp2, "condvar") <- NULL

AbxUsageData <- cbind(AbxUsageData, LinearPredictorComp2_cond,
   LinearPredictorComp2_uncond,
                     PredictLOTComp2, ExpectLOTComp2)

rm(list=ls()[! ls() %in% c("FixefComp1", "FixefComp2", "FixefComp3",
                           "VarsComp1", "VarsComp2", "VarsComp3",
                           "Inpatient_2024", "nested_boot_fast",
                              "HospitalID_PESummary_Boot",
                           "HospitalID_Ranef_Boot", "RanefComp1", "RanefComp2",
                           "num_cores", "ctrl", "df_b", "AbxUsageData",
                              "seed.number", "b",
                           "start_vals_comp1","start_vals_comp2",
                              "start_vals_comp3")])
gc()

#### Component 3 ####
# Create a dataframe only with predictors in component 3
DfComp3 <- df_b %>%  # Create `DfComp3` via a data pipeline
  mutate(AGE_CATAGE_45_54 = ifelse(AGE_CAT == "AGE_45_54", 1, 0),
         AGE_CATAGE_55_64 = ifelse(AGE_CAT == "AGE_55_64", 1, 0),
         AGE_CATAGE_65_74 = ifelse(AGE_CAT == "AGE_65_74", 1, 0),
         AGE_CATAGE_75_84 = ifelse(AGE_CAT == "AGE_75_84", 1, 0),
         AGE_CATAGE_GE85 = ifelse(AGE_CAT == "AGE_GE85", 1, 0))
DfComp3 <- DfComp3 %>%
  select(all_of(VarsComp3))

# Calculate linear predictor for component 3
# Linear predictor for component 2 will be offset variable because the denominator is
   expected LOT
Intercept <- rep(1, nrow(DfComp3))
MatrixComp3 <- as.matrix(cbind(Intercept, DfComp3))

LinearPredictorComp3 <- as.numeric(MatrixComp3 %*% FixefComp3)

AbxUsageData <- cbind(AbxUsageData, LinearPredictorComp3)

# Create offset variable (offset: hospital-specific prediction for component 2)
AbxUsageData <- AbxUsageData %>%
  mutate(OffsetComp3 = log(PredictLOTComp2))
# Fit model with 2024 data for component 3 to estimate HospitalID-specific random
   intercepts
Model2024Comp3 <- glmmTMB(DASC ~ 1 + offset(LinearPredictorComp3) +
   offset(OffsetComp3) + (1|HospitalID),
```

```
                             data = AbxUsageData[AbxUsageData$LOT >0,],
                             family = truncated_nbinom2,
                             start = start_vals_comp3,
                             control = ctrl)

# Generate model-based predictions specific to each hospital
LinearPredictorComp3_cond <- predict(Model2024Comp3,
                                     type = "link",
                                     newdata = AbxUsageData)

# Create offset variable (offset: marginal prediction for component2)
AbxUsageData <- AbxUsageData %>%
  mutate(OffsetComp3 = log(ExpectLOTComp2))
# Generate marginal predictions
LinearPredictorComp3_uncond <- predict(Model2024Comp3,
                                     type = "link",
                                     re.form = ~0,
                                     newdata = AbxUsageData)

PredictDASCComp3 <- exp(LinearPredictorComp3_cond)
ExpectDASCComp3 <- exp(LinearPredictorComp3_uncond)

RanefComp3 <- as.data.frame(ranef(Model2024Comp3)$cond$HospitalID)
RanefComp3 <- RanefComp3 %>%
  rownames_to_column()
colnames(RanefComp3) <- c("HospitalID", "RanefComp3")
attr(RanefComp3, "condvar") <- NULL

AbxUsageData <- cbind(AbxUsageData, LinearPredictorComp3_cond,
    LinearPredictorComp3_uncond,
                      PredictDASCComp3, ExpectDASCComp3)

# Calculate conditional prediction for DASC
AbxUsageData <- AbxUsageData %>%
  mutate(PredictDASCComp3 = PredictProbComp1 * PredictDASCComp3,
         ExpectDASCComp3 = ExpectProbComp1 * ExpectDASCComp3)

AbxUsageData$OffsetComp3 <- NULL

rm(list=ls()[! ls() %in% c("FixefComp1", "FixefComp2", "FixefComp3",
                             "VarsComp1", "VarsComp2", "VarsComp3",
                             "Inpatient_2024", "nested_boot_fast",
                                "HospitalID_PESummary_Boot",
                             "HospitalID_Ranef_Boot", "RanefComp1", "RanefComp2",
                                "RanefComp3",
                             "num_cores", "ctrl", "df_b", "AbxUsageData",
                                "seed.number", "b",
                             "start_vals_comp1","start_vals_comp2",
                                "start_vals_comp3")])
gc()

HospitalID_PESummary <- AbxUsageData %>%
  group_by(HospitalID) %>%
  summarise(ExpectDASCComp3 = sum(ExpectDASCComp3),
            PredictDASCComp3 = sum(PredictDASCComp3)) %>%
  ungroup() %>%
  mutate(S3Metric = PredictDASCComp3/ExpectDASCComp3,
         BootstrapSample = b)

HospitalID_PESummary_Boot <- HospitalID_PESummary_Boot %>%
  dplyr::select(HospitalID, PredictDASCComp3, ExpectDASCComp3,
              S3Metric, BootstrapSample) %>%
  rbind(HospitalID_PESummary)
```

```r
  HospitalID_Ranef <- RanefComp1 %>%
    left_join(RanefComp2, by = "HospitalID") %>%
    left_join(RanefComp3, by = "HospitalID") %>%
    mutate(BootstrapSample = b)

  HospitalID_Ranef_Boot <- rbind(HospitalID_Ranef_Boot, HospitalID_Ranef)

  rm(list=ls()[! ls() %in% c("FixefComp1", "FixefComp2", "FixefComp3",
                             "VarsComp1", "VarsComp2", "VarsComp3",
                             "Inpatient_2024", "nested_boot_fast",
                                 "HospitalID_PESummary_Boot",
                             "HospitalID_Ranef_Boot",
                             "num_cores", "ctrl", "seed.number", "b",
                             "start_vals_comp1","start_vals_comp2",
                                 "start_vals_comp3")])
  gc()
}

HospitalID_PE_CI <- HospitalID_PESummary_Boot %>%
  select(HospitalID, S3Metric) %>%
  group_by(HospitalID) %>%
  summarize(S3Metric_L95 = quantile(S3Metric, probs = 0.025),
            S3Metric_U95 = quantile(S3Metric, probs = 0.975)) %>%
  ungroup()
```

5.10 Visualization of Benchmarking Results

This subsection produces summary visualizations of the benchmarking results, such as ranked plots of S^3 with confidence intervals and reference lines at 1.0 (no deviation from expected). These plots are intended for stewardship stakeholders to quickly identify hospitals with unusually high or low spectrum-weighted antimicrobial use after risk adjustment. Refer to Figure 3 in the main manuscript for the plots.

The plotting code can be adapted to different display choices (e.g., ordering by point estimate vs. upper confidence limit, adding funnel limits, or faceting by hospital complexity).

```r
HospitalID_PE <- HospitalID_PE_PointEstimateSummary %>%
  select(HospitalID, S3Metric) %>%
  left_join(
    HospitalID_PE_CI %>% select(HospitalID, S3Metric_L95, S3Metric_U95),
    by = "HospitalID"
  ) %>%
  mutate(S3MetricRank = rank(S3Metric))

ggplot(HospitalID_PE, aes(x = S3MetricRank, y = S3Metric)) +
  geom_point() +
  geom_segment(aes(y = S3Metric_L95, yend = S3Metric_U95)) +
  geom_hline(yintercept = 1, linetype = 2) +
  scale_y_log10(
    name = "S3 Metric (Logarithmic Scale)",
    breaks = c(0.7, 0.8, 0.9, 1.0, 1.2, 1.4, 1.6, 1.8)
  ) +
  scale_x_continuous(
    name = "Hospital Ranking Based on S3 Metric",
    breaks = c(1, 30, 60, 90, 120)
  ) +
  theme_bw()
```

5.11 Visualization of Three-Axis Evaluation for Each Hospital

In addition to the composite S^3 metric, it would be helpful to visualize the three component-specific evaluations (initiation, duration, and spectrum) simultaneously. This subsection generates per-hospital "three-axis" displays to highlight which component(s) drive a hospital's composite deviation.

These graphics support targeted stewardship interpretation: for example, a hospital with average initiation and duration but high spectrum burden suggests opportunities in antibiotic selection, whereas a hospital with elevated initiation may benefit from diagnostic stewardship or prescribing thresholds. Refer to Figures 2 and 3 in the main manuscript for the plots.

```
#### 1) Triangular radar chart prep (RR/OR + CI per component) ####

# Compute exponentiated (RR/OR-like) point estimates + CI bounds
HospitalIDRanefSummary2 <- HospitalIDRanefSummary %>%
  select(HospitalID, InterceptComp1, InterceptComp2, InterceptComp3) %>%
  left_join(HospitalID_Ranef_CI, by = "HospitalID") %>%
  transmute(
    HospitalID,
    Initiation   = exp(InterceptComp1),
    Initiation_L = exp(RanefComp1_L95),
    Initiation_U = exp(RanefComp1_U95),
    Duration     = exp(InterceptComp2),
    Duration_L   = exp(RanefComp2_L95),
    Duration_U   = exp(RanefComp2_U95),
    Spectrum     = exp(InterceptComp3),
    Spectrum_L   = exp(RanefComp3_L95),
    Spectrum_U   = exp(RanefComp3_U95)
  )

#### 2) Axis scaling (component-specific log scaling) ####

r_max <- 2.0
r_ref <- r_max / 2  # ensures (1,1,1) is equilateral in the triangle coordinates

# Axis definitions
axis_def <- tibble(
  Component = c("Initiation", "Duration", "Spectrum"),
  theta_deg = c(90, 210, 330),
  theta     = theta_deg * pi / 180
)

# Component-specific CI limits (raw scale) -> compute scaling that maps v=1 to r_ref
comp_limits_tbl <- HospitalIDRanefSummary2 %>%
  summarise(
    Initiation_lo = min(Initiation_L, na.rm = TRUE),
    Initiation_hi = max(Initiation_U, na.rm = TRUE),
    Duration_lo   = min(Duration_L,   na.rm = TRUE),
    Duration_hi   = max(Duration_U,   na.rm = TRUE),
    Spectrum_lo   = min(Spectrum_L,   na.rm = TRUE),
    Spectrum_hi   = max(Spectrum_U,   na.rm = TRUE)
  ) %>%
  pivot_longer(
    cols = everything(),
    names_to = c("Component", ".value"),
    names_sep = "_"
  ) %>%
  mutate(
    lo = pmax(lo, .Machine$double.eps),
    hi = pmax(hi, .Machine$double.eps),
    log_lo = log(lo),
    log_hi = log(hi),
    left_span  = 0 - log_lo,     # from lo to 1 on log scale
```

```r
    right_span = log_hi - 0,      # from 1 to hi on log scale
    scale = pmax(
      left_span  / pmax(r_ref, .Machine$double.eps),
      right_span / pmax(r_max - r_ref, .Machine$double.eps),
      na.rm = TRUE
    ),
    scale = ifelse(!is.finite(scale) | scale <= 0, NA_real_, scale)
  ) %>%
  select(Component, lo, hi, scale)

# Turn into a fast lookup named vector for scale
comp_scale <- setNames(comp_limits_tbl$scale, comp_limits_tbl$Component)

scale_to_r <- function(v, component) {
  sc <- comp_scale[[component]]
  if (is.null(sc) || is.na(sc)) return(NA_real_)
  v <- pmax(v, .Machine$double.eps)
  r <- r_ref + log(v) / sc
  pmin(pmax(r, 0), r_max)
}

r_to_value <- function(r, component) {
  sc <- comp_scale[[component]]
  if (is.null(sc) || is.na(sc)) return(NA_real_)
  exp((r - r_ref) * sc)
}

#### 3) Triangle geometry, grids, labels ####

grid_breaks <- c(0.5, 1.0, 1.5, 2.0)

triangle_at_r <- function(r) {
  tibble(theta = axis_def$theta) %>%
    mutate(x = r * cos(theta), y = r * sin(theta)) %>%
    bind_rows(tibble(theta = axis_def$theta[1],
                     x = r * cos(axis_def$theta[1]),
                     y = r * sin(axis_def$theta[1])))
}

grid_df <- purrr::map_dfr(grid_breaks, ~triangle_at_r(.x) %>% mutate(r = .x))
base_df <- triangle_at_r(r_ref)  # v=1 baseline on each axis

axes_df <- axis_def %>%
  transmute(
    x = 0, y = 0,
    xend = r_max * cos(theta),
    yend = r_max * sin(theta)
  )

lab_df <- axis_def %>%
  mutate(
    x = (r_max * 1.35) * cos(theta),
    y = (r_max * 1.35) * sin(theta),
    lab = Component,
    x = case_when(
      Component == "Duration"  ~ x - 0.12,
      Component == "Spectrum"  ~ x + 0.12,
      TRUE ~ x
    ),
    y = case_when(
      Component == "Initiation" ~ y + 0.08,
      TRUE ~ y
    )
```

```
  )

#### 4) Long-format points + CI bands (precompute once for all hospitals) ####

dat_point <- HospitalIDRanefSummary2 %>%
  select(HospitalID, Initiation, Duration, Spectrum) %>%
  pivot_longer(cols = c(Initiation, Duration, Spectrum),
               names_to = "Component", values_to = "Value_raw") %>%
  left_join(axis_def, by = "Component") %>%
  mutate(
    r = purrr::map2_dbl(Value_raw, Component, scale_to_r),
    x = r * cos(theta),
    y = r * sin(theta)
  )

poly_point <- dat_point %>%
  group_by(HospitalID) %>%
  arrange(theta_deg, .by_group = TRUE) %>%
  reframe(x = c(x, x[1]), y = c(y, y[1]))

ci_cols <- c(
  "Initiation_L","Initiation_U",
  "Duration_L","Duration_U",
  "Spectrum_L","Spectrum_U"
)

dat_ci_long <- HospitalIDRanefSummary2 %>%
  select(HospitalID, all_of(ci_cols)) %>%
  pivot_longer(cols = all_of(ci_cols),
               names_to = "key", values_to = "val_raw") %>%
  separate(key, into = c("Component", "Bound"), sep = "_", extra = "merge") %>%
  left_join(axis_def, by = "Component") %>%
  mutate(
    r = purrr::map2_dbl(val_raw, Component, scale_to_r),
    x = r * cos(theta),
    y = r * sin(theta)
  )

ci_inner <- dat_ci_long %>%
  filter(Bound == "L") %>%
  select(HospitalID, Component, theta_deg, x, y)

ci_outer <- dat_ci_long %>%
  filter(Bound == "U") %>%
  select(HospitalID, Component, theta_deg, x, y)

poly_outer <- ci_outer %>%
  group_by(HospitalID) %>%
  arrange(theta_deg, .by_group = TRUE) %>%
  reframe(x = c(x, x[1]), y = c(y, y[1]))

poly_inner_rev <- ci_inner %>%
  group_by(HospitalID) %>%
  arrange(desc(theta_deg), .by_group = TRUE) %>%
  reframe(x = c(x, x[1]), y = c(y, y[1]))

poly_band <- poly_outer %>%
  group_by(HospitalID) %>%
  mutate(order = row_number()) %>%
  ungroup() %>%
  bind_rows(
    poly_inner_rev %>%
      group_by(HospitalID) %>%
```

```
      mutate(order = row_number() + 100) %>%
      ungroup()
  ) %>%
  group_by(HospitalID) %>%
  arrange(order, .by_group = TRUE) %>%
  ungroup()

#### 5) Plot function (one hospital) ####

make_one <- function(h) {
  poly_p <- poly_point %>% filter(HospitalID == h)
  pts_p  <- dat_point  %>% filter(HospitalID == h)
  band   <- poly_band  %>% filter(HospitalID == h)

  ggplot() +
    geom_path(data = grid_df, aes(x = x, y = y, group = r),
              color = "grey85", linewidth = 0.6) +
    geom_path(data = base_df, aes(x = x, y = y),
              color = "grey30", linewidth = 0.8, linetype = "dashed") +
    geom_segment(data = axes_df, aes(x = x, y = y, xend = xend, yend = yend),
                 color = "grey70", linewidth = 0.6) +
    geom_polygon(data = band, aes(x = x, y = y),
                 fill = "grey70", alpha = 0.25, color = NA) +
    geom_path(data = poly_p, aes(x = x, y = y),
              color = "black", linewidth = 1.0) +
    geom_point(data = pts_p, aes(x = x, y = y),
               size = 2.3, color = "black") +
    geom_text(data = lab_df, aes(x = x, y = y, label = lab),
              size = 5.2, fontface = "bold") +
    labs(title = paste0(h, " (HospitalID: ", h, ")")) +
    coord_equal(
      xlim = c(-r_max * 1.55, r_max * 1.55),
      ylim = c(-r_max * 1.35, r_max * 1.60),
      expand = FALSE
    ) +
    theme_void(base_size = 12) +
    theme(
      plot.title = element_text(hjust = 0.5, face = "bold", size = 14),
      plot.margin = margin(20, 25, 20, 25)
    )
}

#### 6) Save one hospital per file ####

safe_stub <- function(x) {
  # Make filenames safe across OSes
  x %>%
    str_replace_all("\\s+", "") %>%
    str_replace_all("[^A-Za-z0-9_\\-]", "")
}

for (h in unique(HospitalIDRanefSummary2$HospitalID)) {
  p <- make_one(h)

  fn_base <- paste0("Figure_", safe_stub(h), "_HospitalID", safe_stub(h),
     "_CItriangle_max2")
  out_svg <- paste0(fn_base, ".svg")

  ggsave(out_svg, p, width = 6.8, height = 6.2)

  print(p)
}
```

Bibliography for Technical Supplement

[1] Satoshi Kakiuchi et al. "Days of Antibiotic Spectrum Coverage: A Novel Metric for Inpatient Antibiotic Consumption ". In: *Clinical Infectious Diseases* 75.4 (Dec. 2021), pp. 567–576. ISSN: 1058-4838. DOI: 10.1093/cid/ciab1034. eprint: https://academic.oup.com/cid/article-pdf/75/4/567/45787147/ciab1034.pdf. URL: https://doi.org/10.1093/cid/ciab1034.

[2] Michihiko Goto et al. "Metrics Selection and Risk Adjustment Methods to Benchmark Inpatient Antibiotic Use". In: *JAMA Network Open* 8.6 (June 2025), e2514989–e2514989. ISSN: 2574-3805. DOI: 10.1001/jamanetworkopen.2025.14989. eprint: https://jamanetwork.com/journals/jamanetworkopen/articlepdf/2835172/goto_2025_oi_250488_1748985884.1237.pdf. URL: https://doi.org/10.1001/jamanetworkopen.2025.14989.

[3] Nicolai Meinshausen and Peter BÃ¼hlmann. "Stability selection". In: *Journal of the Royal Statistical Society Series B* 72.4 (Sept. 2010), pp. 417–473. DOI: 10.1111/j.1467-9868.2010.00740.x. URL: https://ideas.repec.org/a/bla/jorssb/v72y2010i4p417-473.html.

[4] Robert Tibshirani. "Regression Shrinkage and Selection Via the Lasso". In: *Journal of the Royal Statistical Society: Series B (Methodological)* 58.1 (Dec. 2018), pp. 267–288. ISSN: 0035-9246. DOI: 10.1111/j.2517-6161.1996.tb02080.x. eprint: https://academic.oup.com/jrsssb/article-pdf/58/1/267/49098631/jrsssb_58_1_267.pdf. URL: https://doi.org/10.1111/j.2517-6161.1996.tb02080.x.

[5] Cun-Hui Zhang. "NEARLY UNBIASED VARIABLE SELECTION UNDER MINIMAX CONCAVE PENALTY". In: *The Annals of Statistics* 38.2 (2010), pp. 894–942. ISSN: 00905364, 21688966. URL: http://www.jstor.org/stable/25662264 (visited on 02/22/2026).

[6] Joseph M. Hilbe. *Negative Binomial Regression.* 2nd ed. Cambridge University Press, 2011.

[7] Mollie E. Brooks et al. "glmmTMB Balances Speed and Flexibility Among Packages for Zero-inflated Generalized Linear Mixed Modeling". In: *The R Journal* 9 (2 2017). https://doi.org/10.32614/RJ-2017-066, pp. 378–400. ISSN: 2073-4859. DOI: 10.32614/RJ-2017-066.

[8] C. A. Field and A. H. Welsh. "Bootstrapping Clustered Data". In: *Journal of the Royal Statistical Society Series B: Statistical Methodology* 69.3 (May 2007), pp. 369–390. ISSN: 1369-7412. DOI: 10.1111/j.1467-9868.2007.00593.x. eprint: https://academic.oup.com/jrsssb/article-pdf/69/3/369/49795413/jrsssb_69_3_369.pdf. URL: https://doi.org/10.1111/j.1467-9868.2007.00593.x.

[9] James R. Carpenter, Harvey Goldstein, and Jon Rasbash. "A novel bootstrap procedure for assessing the relationship between class size and achievement". In: *Journal of the Royal Statistical Society Series C* 52.4 (Oct. 2003), pp. 431–443. DOI: 10.1111/1467-9876.00415. URL: https://ideas.repec.org/a/bla/jorssc/v52y2003i4p431-443.html.
